# Selective construction of dispiro[indoline-3,2'-quinoline-3',3''-indoline] and dispiro[indoline-3,2'-pyrrole-3',3''-indoline] via three-component reaction

**DOI:** 10.3762/bjoc.19.91

**Published:** 2023-08-22

**Authors:** Ziying Xiao, Fengshun Xu, Jing Sun, Chao-Guo Yan

**Affiliations:** 1 College of Chemistry & Chemical Engineering, Yangzhou University, Jiangsu, Yangzhou 225002, China

**Keywords:** cascade reaction, dimedone, isatin, 3-methyleneoxindole, multicomponent reaction, spirooxindole

## Abstract

A convenient synthetic procedure for the construction of novel dispirooxindole motifs was successfully developed by base-promoted three-component reaction of ammonium acetate, isatins and in situ-generated 3-isatyl-1,4-dicarbonyl compounds. The piperidine-promoted three-component reaction of ammonium acetate, isatins and the in situ-generated dimedone adducts of 3-ethoxycarbonylmethyleneoxindoles afforded mutlifunctionalized dispiro[indoline-3,2'-quinoline-3',3''-indoline] derivatives in good yields and with high diastereoselectivity. On the other hand, a similar reaction of the dimedone adducts of 3-phenacylideneoxindoles afforded unique dispiro[indoline-3,2'-pyrrole-3',3''-indoline] derivatives with a cyclohexanedione substituent. A plausible reaction mechanism is proposed to explain the formation of the different spirooxindoles.

## Introduction

**Scheme 1:**
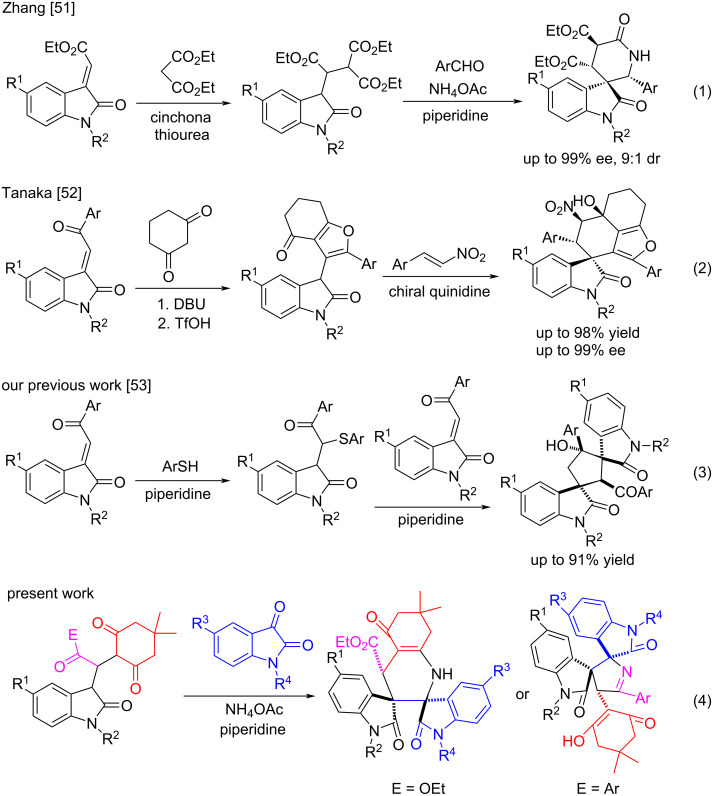
Representative cascade reactions of Michael adducts of 3-methyleneoxindoles.

Spirooxindole is one important privileged structural skeleton and is found in many bioactive natural and synthetic compounds [1–3]. It is known that many spirooxindole derivatives show important biological activities [4–6]. On the other hand, a wide range of differently substituted spirooxindoles exist [7–9]. Therefore, the development of efficient synthetic methodologies for diverse spirooxindoles have become one of the hottest research fields in organic and pharmaceutical chemistry [10–11]. In order to synthesize diverse spirooxindole derivatives, commercially available isatins and easily obtainable 3-methyleneoxindolines were the most employed building blocks [12–15]. On the other hand, Morita–Baylis–Hillman (MBH) carbonates of isatins, which could be easily prepared by the MBH reactions of isatins with acrylonitrile or alkyl acrylates also became valuable synthons [16–25]. The active 3-methyleneoxindoles could act as Michael acceptors, 1,3-dipolarophiles, 1,4-dienophiles and other multiple substrates. They have been widely employed as key component to finish many multicomponent and domino reactions [26–35]. Recently, the Michael adduct of 3-methyleneoxindoles with various nucleophiles, especially active methylene compounds, have attracted much attentions [36–41]. This kind of Michael adduct has more than one reactive site and could proceed domino reactions with various electrophiles [42–49]. In this respect, several elegant domino or multicomponent reactions have been successfully developed to construct multifunctionalized or polycyclic spirooxindoles. For example, Zhang successfully developed a recyclable bifunctional cinchona/thiourea-catalyzed four-component Michael/Mannich cyclization sequence for the asymmetric synthesis of spirooxindoles, in which the in situ-generated Michael adduct of 3-ethoxycarbonylmethyleneoxindole underwent a Mannich reaction and annulation reaction with in situ-generated aldimines (reaction 1 in Scheme 1) [50–51]. Tanaka reported chiral quinidine derivative-catalyzed Michael–Henry cascade reactions of nitrostyrenes with previously prepared oxindole-functionalized dihydrobenzofuranones (reaction 2 in Scheme 1) [52]. We reported a piperidine-promoted domino reaction of thiophenols and two molecules of 3-phenacylideneoxindoles to give multifunctionalized dispirocyclopentanebisoxindoles (reaction 3 in Scheme 1), in which the in situ-generated adduct of thiophenol and 3-phenacylideneoxindole was believed to be the key intermediate [53–55]. Inspired by these elegant synthetic protocols and in continuation of our aim to develop convenient reactions for the synthesis of diverse spiro compounds [56–62], we investigated the base-promoted annulation reaction of dimedone adducts of 3-methyleneoxindoles, with isatin and ammonium acetate. It was unexpectedly found that novel dispiro[indoline-3,2'-quinoline-3',3''-indoline] and dispiro[indoline-3,2'-pyrrole-3',3''-indoline] were selectively produced by using differently substituted 3-methyleneoxindoles (reaction 4 in Scheme 1). Herein, we wish to report these interesting results.

## Results and Discussion

At first, 3-isatyl-1,4-dicarbonyl compound **1** was prepared by DBU-catalyzed Michael addition reaction of dimedone and ethyl 2-(2-oxoindolin-3-ylidene)acetate in toluene according to the published method [52]. Then, the reaction conditions of the three-component reaction of isatyl adduct **1a** (0.20 mmol), isatin **2a** (0.20 mmol) and ammonium acetate (0.5 mmol) were examined according to Zhang and co-workers reported reaction (reaction 1 in Scheme 1) [12]. In the presence of piperidine, the reaction in methanol at room temperature did not yield the product (Table 1, entry 1). However, the reaction in methanol at elevated temperature gave the expected spiro compound **3a** in 51% and 48% yields, respectively (Table 1, entries 2 and 3). The reaction in other solvents such as acetonitrile, toluene and ethyl acetate at 50 °C gave the desired product **3a** in very low yields (Table 1, entries 4–6). When the reaction was carried out in a mixture of toluene and methanol (v/v = 2:1) in the presence of piperidine, the yield of **3a** increased to 60% (Table 1, entry 7). When DABCO or DBU was employed as base, the yield of **3a** decreased to 36% and 29% yield, respectively (Table 1, entries 8 and 9). When the loading of ammonium acetate was increased to 0.8 mmol and 1.0 mmol, the yield of **3a** increased to 85% and 82% (Table 1, entries 10 and 11). Prolonging the reaction time did not increase the yield of product **3a** (Table 1, entry 12). Therefore, the optimized reaction conditions found for this three-component reaction are the use of a mixture of methanol and toluene at 50 °C for seven hours in the presence of piperidine.

**Table 1:** Optimizing reaction conditions.^a^

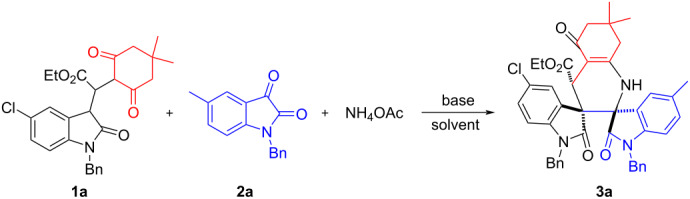					

Entry	Base	Solvent	*T* (°C)	Time (h)	Yield (%)^b^

1	piperidine	MeOH	25	7	nr
2	piperidine	MeOH	50	7	51
3	piperidine	MeOH	60	7	48
4	piperidine	CH_3_CN	50	7	20
5	piperidine	toluene	50	7	32
6	piperidine	EtOAc	50	7	26
7	piperidine	toluene/MeOH	50	7	60
8	DABCO	toluene/MeOH	50	7	36
9	DBU	toluene/MeOH	50	7	29
10	piperidine^c^	toluene/MeOH	50	7	85
11	piperidine^d^	toluene/MeOH	50	7	82
12	piperidine^c^	toluene/MeOH	50	12	84

^a^Reaction conditions: 3-isatyl-1,4-dicarbonyl compound **1** (0.20 mmol), isatin **2** (0.20 mmol), NH_4_OAc (0.50 mmol), base (0.30 mmol), solvent (4.0 mL). ^b^Isolated yields. ^c^NH_4_OAc (0.8 mmol) was used; ^d^NH_4_OAc (1.0 mmol) was used.

**Figure 1:**
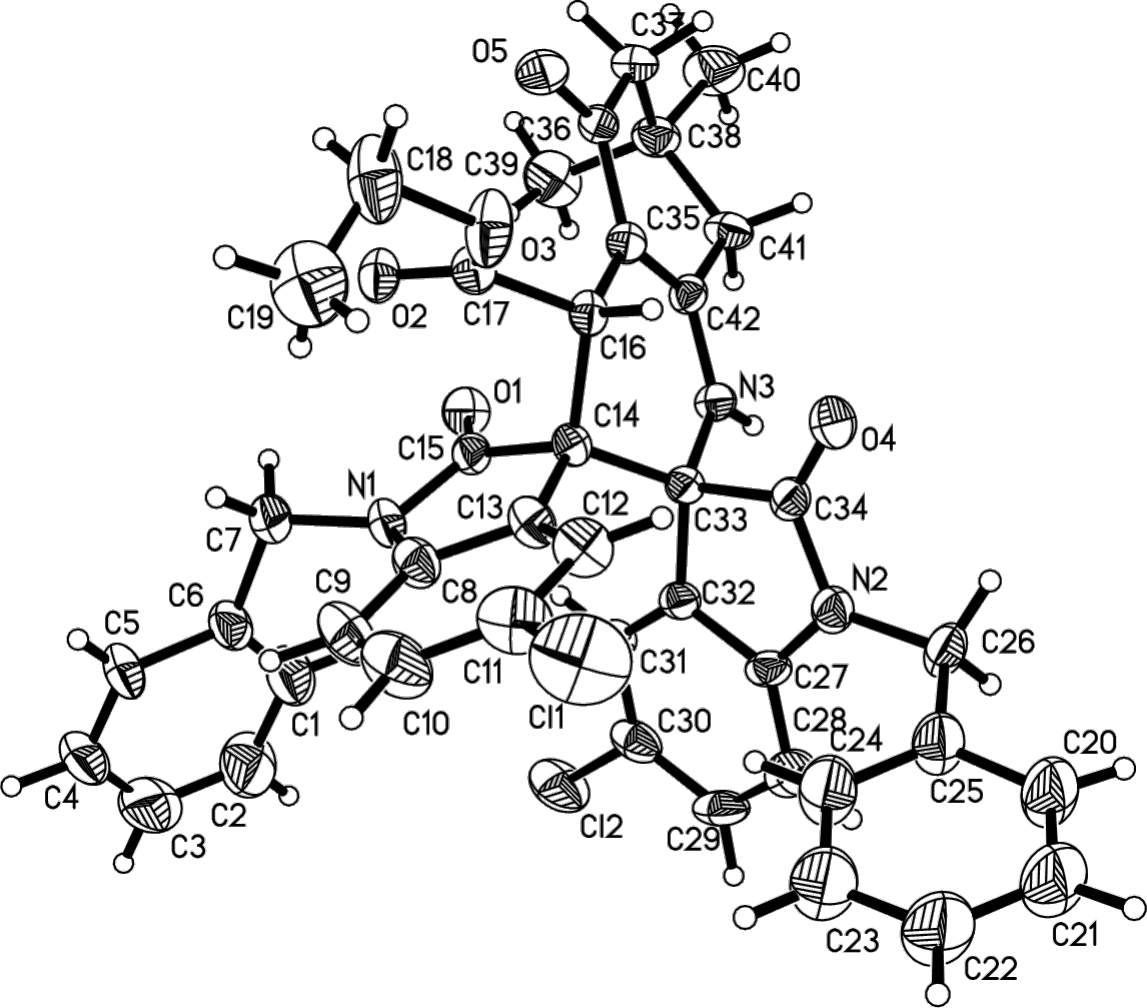
Crystal structure of dispiro compound **3a**.

With the optimized reaction conditions in hand, the scope of the reaction was investigated by using various substrates. The results are summarized in Table 2. All reactions proceeded smoothly to give the desired dispiro compounds **3a–m** in satisfactory yields. Various isatins with different substituents can be successfully used in the reaction. The substituents showed marginal effects on the yields. On the other hand, the dimedone adducts of alkyl 2-(2-oxoindolin-3-ylidene)acetate with various substituents were also successfully employed in the reaction to give the desired products. It can be seen that the dimedone adducts of alkyl 2-(2-oxoindolin-3-ylidene)acetate with 5-chloro and 5-fluoro substituent gave the spiro compounds **3a–k** in satisfactory yields. However, the dimedone adducts of ethyl 2-(2-oxoindolin-3-ylidene)acetate itself and its derivatives with 5-methyl group gave the products **3l** and **3m** in moderate yields. The chemical structures of the obtained dispiro compounds **3a–m** were fully characterized by IR, HRMS, ^1^H and ^13^C NMR spectroscopy. Because of the three chiral carbon atoms in the product, several diastereomers might be formed in the reaction. However, TLC monitoring and ^1^H NMR spectra of the crude products clearly indicated that only one diastereoisomer was predominately produced in the reaction, while the other possible diastereomers were not detected. This result shows that this reaction has a high diastereoselectivity due to the large steric effect of two oxindole scaffolds and the thermodynamically controlling effect. The single crystal structure of compound **3a** was determined by X-ray crystallographic diffraction (Figure 1). From Figure 1 it can be seen that the two scaffolds of oxindole at neighboring positions are in *trans*-configuration. The ethoxycarbonyl group is also in *trans*-position to the carbonyl group in the neighboring oxindole scaffold. Therefore, it can be concluded that the obtained dispiro compounds **3a–m** have this kind of relative configuration on the basis of ^1^H NMR spectra and crystal structure determination. It should be pointed out that ammonium acetate was employed as nitrogen source in this three-component reaction. For investigating the scope of this reaction, aniline was also used in the reaction, but in this case no expected dispirooxindoles could be obtained.

**Table 2:** Synthesis of the dispirooxindoles **3a–m**.^a^

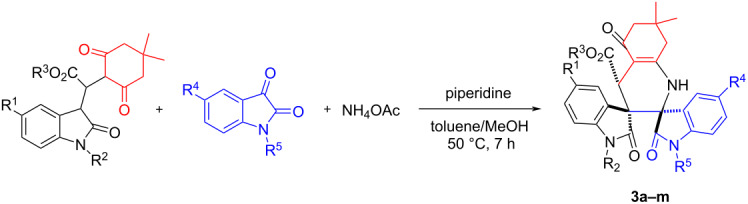							

Entry	Compd	R^1^	R^3^	R^2^	R^4^	R^5^	Yield (%)^b^

1	**3a**	Cl	C_2_H_5_	Bn	CH_3_	Bn	85
2	**3b**	Cl	C_2_H_5_	Bn	Cl	Bn	78
3	**3c**	Cl	C_2_H_5_	Bn	Cl	*n*-Bu	82
4	**3d**	Cl	C_2_H_5_	Bn	CH_3_	*n*-Bu	81
5	**3e**	Cl	C_2_H_5_	Bn	H	H	69
6	**3f**	Cl	C_2_H_5_	Bn	CH_3_	H	70
7	**3g**	Cl	C_2_H_5_	Bn	Cl	H	72
8	**3h**	Cl	C_2_H_5_	Bn	F	H	70
9	**3i**	Cl	C_2_H_5_	H	CH_3_	Bn	68
10	**3j**	Cl	CH_3_	Bn	CH_3_	Bn	72
11	**3k**	F	CH_3_	Bn	CH_3_	Bn	69
12	**3l**	H	C_2_H_5_	Bn	Cl	Bn	42
13	**3m**	CH_3_	C_2_H_5_	Bn	Cl	Bn	48

^a^Reaction conditions: 3-isatyl-1,4-dicarbonyl compound **1** (0.20 mmol), isatin **2** (0.20 mmol), NH_4_OAc (0.80 mmol), piperidine (0.30 mmol), toluene (2.0 mL), MeOH (2.0 mL), 50 °C, 7 h. ^b^Isolated yields.

**Figure 2:**
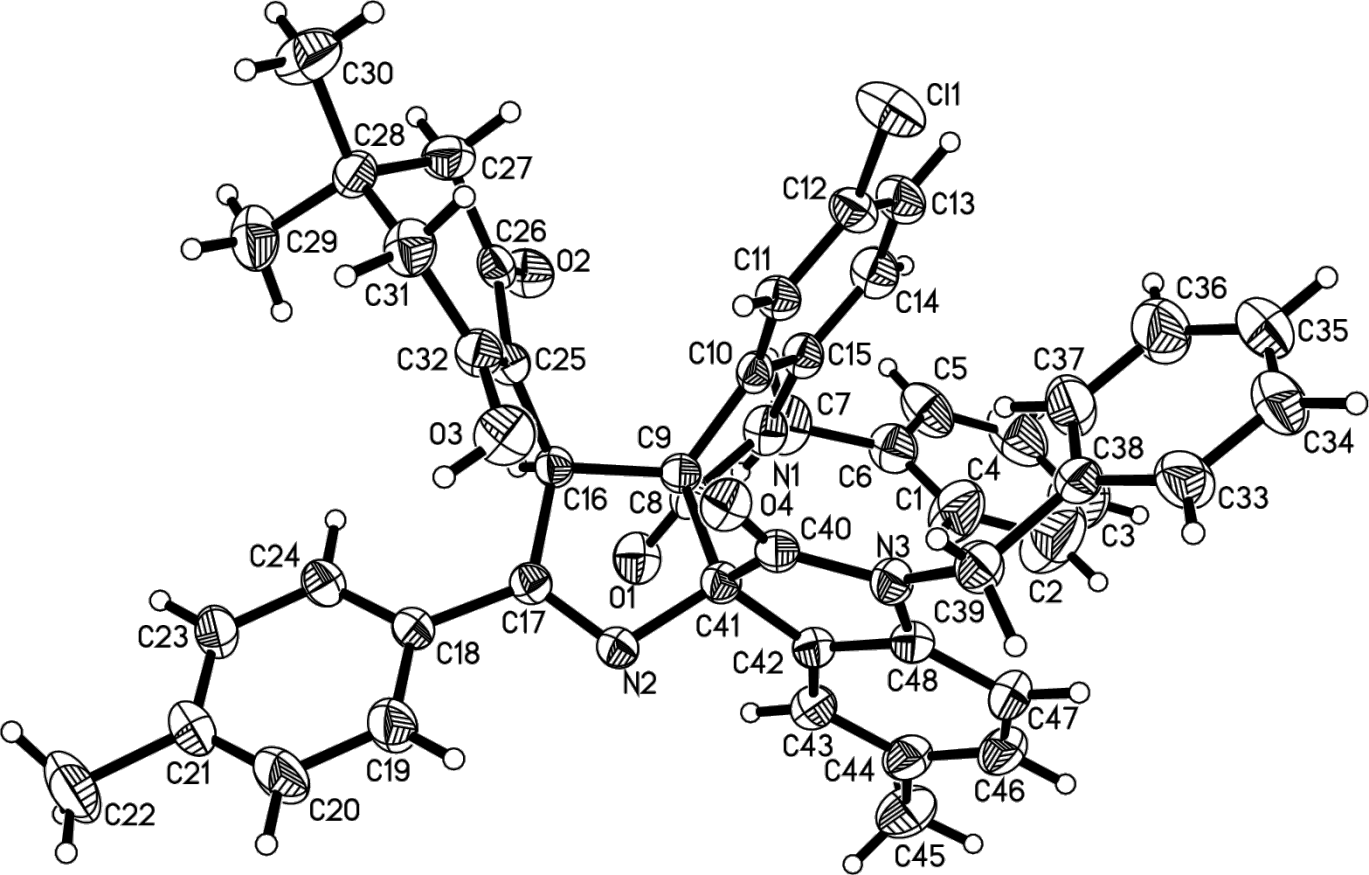
Crystal structure of compound **4a**.

In order to investigate the scope of this reaction, similar dimedone adducts of 3-phenacylideneoxindoles were used in the three-component reaction. To our surprise, instead of the above mentioned dispiro[indoline-3,2'-quinoline-3',3''-indolines] **3a–m**, the novel dispiro[indoline-3,2'-pyrrole-3',3''-indoline] derivatives **4a–i** were obtained in high yields. The results are summarized in Table 3. The structural analysis showed that the carbonyl group of the dimedone does not take part in the further cyclization reaction, while the carbonyl group of the benzoyl group participated in the annulation reaction to give the pyrrolidyl ring. This result clearly indicated that the adducts of 3-phenacylideneoxindoles showed different reactivity to that of the adducts of 3-ethoxycarbonylmethyleneoxindoles. For confirming the chemical structures of dispirooxindoles **4a–i**, the single crystal structure of compound **4a** was determined by X-ray diffraction (Figure 2). In Figure 2, the two oxindole scaffolds are in *trans*-position. The dimedone moiety is also in *trans*-position to the carbonyl group in the neighboring oxindole scaffold. To demonstrate the synthetic value of this three-component reaction, 3-phenacylideneoxindole adducts of 1,3-cyclohexanedione were also employed in the reaction. In the presence of piperidine, the three-component reaction proceeded smoothly at room temperature in twelve hours to give the expected dispiro[indoline-3,2'-pyrrole-3',3''-indoline] derivatives **4j–p** in satisfactory yields. The 1,3-cyclohexanedione moiety does not take part in the further cyclization process. These results show that this reaction is largely general. The ^1^H NMR spectra of the obtained compounds **4j–p** clearly show similar chemical shifts of the characteristic groups as the spiro compounds **4a–i**. Therefore, it can be concluded that the spiro compounds **4j–p** have the same relative configuration as the spiro compounds **4a–i**.

**Table 3:** Synthesis of dispirooxindoles **4a–p**.^a^

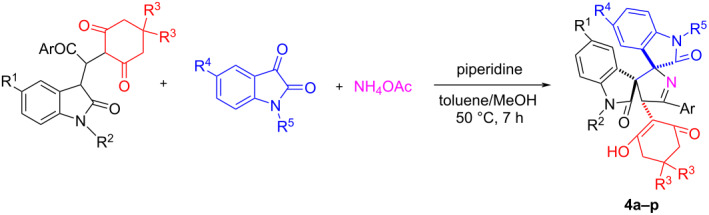								

Entry	Compd	R^1^	R^2^	Ar	R^3^	R^4^	R^5^	Yield (%)^b^

1	**4a**	Cl	Bn	*p*-CH_3_C_6_H_4_	CH_3_	CH_3_	Bn	85
2	**4b**	Cl	Bn	*p*-CH_3_C_6_H_4_	CH_3_	Cl	Bn	68
3	**4c**	Cl	Bn	*p*-CH_3_C_6_H_4_	CH_3_	H	Bn	75
4	**4d**	Cl	Bn	*p*-CH_3_C_6_H_4_	CH_3_	CH_3_	*n*-Bu	72
5	**4e**	Cl	Bn	*p*-ClC_6_H_4_	CH_3_	CH_3_	Bn	63
6	**4f**	CH_3_	Bn	*p*-ClC_6_H_4_	CH_3_	CH_3_	Bn	51
7	**4g**	Cl	Bn	*p*-CH_3_C_6_H_4_	CH_3_	H	H	34
8	**4h**	Cl	*n*-Bu	*p*-CH_3_OC_6_H_4_	CH_3_	CH_3_	Bn	65
9	**4i**	Cl	H	*p*-CH_3_OC_6_H_4_	CH_3_	CH_3_	Bn	62
10	**4j**	Cl	Bn	*p*-CH_3_C_6_H_4_	H	CH_3_	Bn	71
11	**4k**	Cl	Bn	*p*-CH_3_C_6_H_4_	H	Cl	Bn	68
12	**4l**	Cl	Bn	*p*-CH_3_C_6_H_4_	H	H	Bn	64
13	**4m**	Cl	Bn	*p*-CH_3_C_6_H_4_	H	Cl	*n*-Bu	65
14	**4n**	Cl	Bn	*p*-CH_3_C_6_H_4_	H	CH_3_	H	52
15	**4o**	Cl	Bn	*p*-CH_3_C_6_H_4_	H	CH_3_	*n*-Bu	63
16	**4p**	Cl	Bn	*p*-ClC_6_H_4_	H	CH_3_	Bn	73

^a^Reaction conditions: 3-isatyl-1,4-dicarbonyl compound **1** (0.20 mmol), isatin **2** (0.20 mmol), NH_4_OAc (0.80 mmol), piperidine (0.30 mmol), toluene (2.0 mL), MeOH (2.0 mL), 50 °C, 7 h. ^b^Isolated yields.

**Scheme 2:**
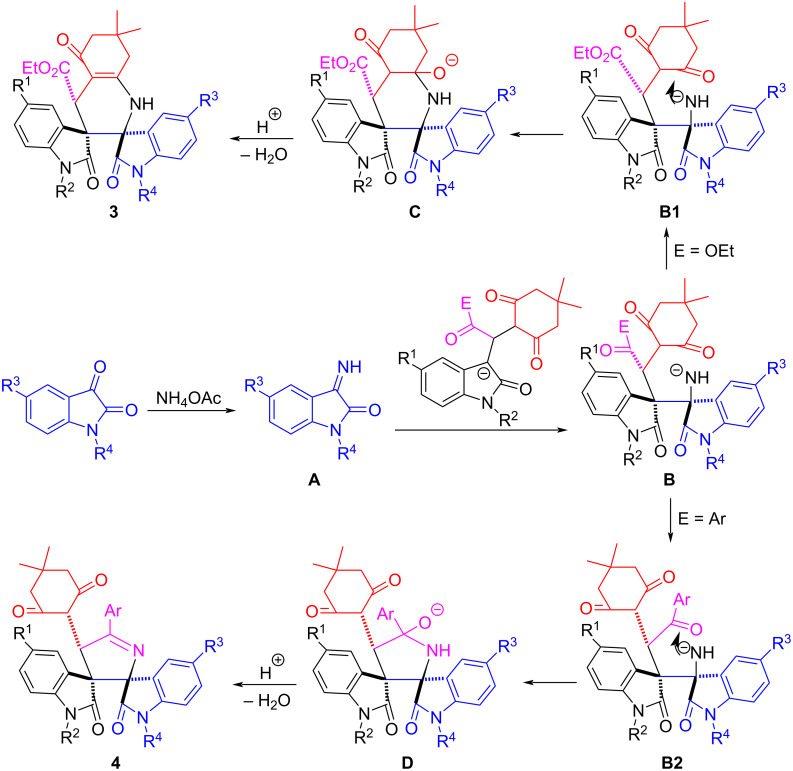
Proposed reaction mechanism.

In order to explain the formation of different cyclic compounds, a plausible reaction mechanism was proposed in Scheme 2 on the basis of the present experiments and the previous works [51–53]. Firstly, 3-isatyl-1,4-dicarbonyl compound **1** was converted to a carbanion in the presence of base. In the meantime, the condensation of isatin **2** with ammonium acetate gave the 3-iminoisatin intermediate **A**. Secondly, Michael addition of the in situ-generated carbanion of the 3-isatyl-1,4-dicarbonyl compound **1** to 3- iminoisatin **A** gave intermediate **B**. In the case of intermediate **B1** with an ethoxycarbonyl group, the nucleophilic addition of the amino anion to the carbonyl group in the of 1,3-cyclohexanedione scaffold resulted in cyclic intermediate **C**. Thirdly, the elimination of water from intermediate **C** gave the isolated product **3**. In the case of the intermediate **B2** with a benzoyl group, there are two kinds of reactive carbonyl groups in intermediate **B2**, one carbonyl group is in the benzoyl moiety and another carbonyl group is in the 1,3-cyclohexanedione part. In this case the amino group selectively attacked the benzoyl group to give cyclic intermediate **D**, while the two carbonyl groups in the 1,3-cyclohexanedione moiety remained unreacted. Then, the final product **4** was formed by elimination of water. Thus, the spiro compounds **3** and **4** were selectively produced due to the different reaction process. Thus, the 3-isatyl-1,4-dicarbonyl compounds derived from 3-ethoxycarbonylmethyloxindoles and 3-phenacylideneoxindoles resulted in the two different novel dispirooxindole skeletons.

## Conclusion

In summary, we have investigated the base-promoted multicomponent reaction of 3-methyleneoxindoles, dimedone, isatins and ammonium acetate. The reaction showed very interesting molecular diversity and diastereoselectivity. This reaction provided efficient synthetic protocols for the synthesis of dispiro[indoline-3,2'-quinoline-3',3''-indoline] and dispiro[indoline-3,2'-pyrrole-3',3''-indoline] derivatives. A plausible reaction mechanism was proposed to explain the selective formation of the different polycyclic compounds. This reaction has the advantages of using readily available materials, simple reaction conditions, satisfactory yields, high diastereoselectivity and atomic economy, which enable this reaction potential synthetic applications in heterocyclic chemistry and medicinal chemistry.

## Experimental

### General procedure for the preparation of compounds **3a–m**

To a round flask was added 3-isatyl-1,4-dicarbonyl compound **1** (0.20 mmol), isatin **2** (0.20 mmol), ammonium acetate (0.80 mmol), piperidine (0.30 mmol), toluene (2.0 mL) and methanol (2.0 mL). The mixture was heated at 50 °C for seven hours. After removing the solvent by rotatory evaporation at reduced pressure, the residue was subjected to column chromatography (silicon gel, 300–400 mesh) with petroleum ether and ethyl acetate (v/v = 1:1) to give the pure product for analysis.

**Ethyl *rel*-(3*R*,3'*S*,4'*R*)-1,1''-dibenzyl-5,5''-dichloro-7',7'-dimethyl-2,2'',5'-trioxo-1',4',5',6',7',8'-hexahydrodispiro[indoline-3,2'-quinoline-3',3''-indoline]-4'-carboxylate (3a):** White solid, 78%, mp 280–282 °C; ^1^H NMR (400 MHz, CDCl_3_) δ 7.43 (d, *J* = 2.0 Hz, 1H, ArH), 7.34–7.28 (m, 6H, ArH), 7.51 (s, 1H, ArH), 7.24 (s, 2H, ArH), 7.23 (s, 1H, ArH), 7.20–7.17 (m, 3H, ArH), 7.11–7.09 (m, 1H, ArH), 7.0–6.98 (m, 1H, ArH), 6.40 (d, *J* = 8.4 Hz, 1H, ArH), 6.26 (d, *J* = 8.4 Hz, 1H, ArH), 5.01 (d, *J* = 16.4, 1H, CH_2_), 4.89 (d, *J* = 15.6 Hz, 1H, CH_2_), 4.78 (d, *J* = 15.6 Hz, 1H, CH_2_), 4.76 (s, 1H, NH), 4.74 (s, 1H, CH), 4.66 (d, *J* = 16.4 Hz, 1H, CH_2_), 3.94–3.87 (m, 1H, CH_2_), 3.84–3.77 (m, 1H, CH_2_), 2.46 (d, *J* = 16.0 Hz, 1H, CH_2_), 2.39 (d, *J* = 16.0 Hz, 1H, CH_2_), 2.37–2.34 (m, 2H, CH_2_), 1.30 (s, 3H, CH_3_), 1.15 (s, 3H, CH_3_), 0.76 (t, *J* = 7.2 Hz, 3H, CH_3_) ppm; ^13^C NMR (101 MHz, CDCl_3_) δ 193.0, 173.8, 171.9, 171.2, 155.6, 142.0, 141.8, 134.8, 134.1, 131.0, 129.3, 129.1, 128.8, 128.7, 128.6, 127.9, 127.5, 127.5, 127.1, 127.1, 127.0, 126.2, 125.9, 125.6, 124.9, 110.9, 110.3, 102.3, 62.3, 60.3, 50.0, 49.4, 44.4, 44.2, 42.5, 42.4, 32.9, 29.0, 27.6, 13.5 ppm; IR (KBr) ν: 3504, 3024, 3010, 2995, 2985, 1847, 1711, 1603, 1517, 1400, 1299, 1250, 1053, 953, 841 cm^−1^; HRMS (ESI-TOF): [M + Na]^+^ calcd. for C_42_H_37_Cl_2_N_3_O_5_, 756.2002; found, 756.1989.

### General procedure for the preparation of compounds **4a–p**

To a round flask was added 3-isatyl-1,4-dicarbonyl compound **1** (0.20 mmol), isatin **2** (0.20 mmol), ammonium acetate (0.80 mmol), piperidine (0.30 mmol), toluene (2.0 mL) and methanol (2.0 mL). The mixture was heated at 50 °C for seven hours. After removing the solvent by rotatory evaporation at reduced pressure, the residue was subjected to column chromatography (silicon gel, 300–400 mesh) with petroleum ether and ethyl acetate (v/v = 4:1) to give the pure product for analysis.

***rel-*(3*R*,3'*S*,4'*R*)-1,1''-Dibenzyl-5''-chloro-4'-(2-hydroxy-4,4-dimethyl-6-oxocyclohex-1-en-1-yl)-5-methyl-5'-(*p*-tolyl)-4'*H*-dispiro[indoline-3,2'-pyrrole-3',3''-indoline]-2,2''-dione (4a):** White solid, 60%, mp 250–251 °C; ^1^H NMR (400 MHz, CDCl_3_) δ 10.86 (s, 1H, OH), 7.80 (d, *J* = 8.0 Hz, 2H, ArH), 7.51 (s, 1H, ArH), 7.21 (d, *J* = 8.4 Hz, 2H, ArH), 7.18–7.11 (m, 4H, ArH), 7.10–7.05 (m, 3H, ArH), 7.00–6.97 (m, 2H, ArH), 6.76 (d, *J* = 7.2 Hz, 2H, ArH), 6.65 (d, *J* = 7.6 Hz, 2H, ArH), 6.46 (d, *J* = 8.0 Hz, 1H, ArH), 6.28 (d, *J* = 8.4 Hz, 1H, ArH), 5.65 (s, 1H, CH), 5.18 (d, *J* = 16.4 Hz, 1H, CH_2_), 5.09 (d, *J* = 16.0, 1H, CH_2_), 4.47 (d, *J* = 4.8 Hz, 1H, CH_2_), 4.43 (d, *J* = 5.2 Hz, 1H, CH_2_), 2.41 (d, *J* = 26.8 Hz, 1H, CH_2_), 2.40 (s, 3H, CH_3_), 2.21 (d, *J* = 18.4 Hz, 1H, CH_2_), 2.10 (s, 1H, CH_3_), 2.05 (s, 1H, CH_2_), 1.87 (d, *J* = 16.0 Hz, 1H, CH_2_), 1.00 (s, 3H, CH_3_), 0.99 (s, 3H, CH_3_) ppm; ^13^C NMR (101 MHz, CDCl_3_) δ 197.5, 181.6, 177.4, 177.2, 173.0, 142.8, 142.2, 140.6, 134.7, 134.3, 133.9, 130.2, 130.0, 129.1, 128.8, 128.7, 128.5, 128.4, 127.5, 127.3, 127.1, 127.0, 126.4, 126.2, 126.1, 126.0, 112.1, 109.8, 109.7, 87.2, 61.8, 53.7, 50.0, 44.5, 44.4, 43.6, 30.7, 29.9, 26.3, 21.6, 20.9 ppm; IR (KBr) ν: 3756, 3056, 3023, 2984, 2988, 1832, 1792, 1526, 1545, 1368, 1285, 1145, 1025, 956, 882 cm^−1^; HRMS (ESI-TOF): [M + H]^+^ calcd. for C_48_H_44_ClN_3_O_5_, 760.2937; found, 760.2921.

## Supporting Information

The crystallographic data of compounds **3a** (CCDC 2223538) and **4a** (CCDC 2223539) have been deposited at the Cambridge Crystallographic Database Center (http://www.ccdc.cam.ac.uk).

File 1 Characterization data, ^1^H NMR, ^13^C NMR, and HRMS spectra of the compounds.
