# Supplementary material for: Selective construction of dispiro[indoline-3,2'-quinoline-3',3''-indoline] and dispiro[indoline-3,2'-pyrrole-3',3''-indoline] via three-component reaction
Source: Beilstein J Org Chem. 2023 Aug 22;19:1234–42. doi: 10.3762/bjoc.19.91 (PMC10478003; doi:10.3762/bjoc.19.91)
Supplement: File 1 — Characterization data, 1H NMR, 13C NMR, and HRMS spectra of the compounds. [file Beilstein_J_Org_Chem-19-1234-s001.pdf]

BEILSTEIN JOURNAL OF ORGANIC CHEMISTRY

Supporting Information

for

Selective construction of dispiro[indoline-3,2'-quinoline-3',3''-indoline] and dispiro[indoline-3,2'-pyrrole-3',3''-indoline] via three-component reaction

Ziying Xiao, Fengshun Xu, Jing Sun and Chao-Guo Yan

Beilstein J. Org. Chem. **2023,** *19,* 1234–1242. doi:10.3762/bjoc.19.91

Characterization data, ^{1}H NMR, ^{13}C NMR, and HRMS spectra of the compounds

License and Terms: This is a supporting information file under the terms of the Creative Commons Attribution License (https://creativecommons.org/licenses/by/4.0). Please note that the reuse, redistribution and reproduction in particular requires that the author(s) and source are credited and that individual graphics may be subject to special legal provisions.
The license is subject to the *Beilstein Journal of Organic Chemistry* terms and conditions: (https://www.beilstein-journals.org/bjoc/terms)

Ethyl *rel*-(3*R*,3'*S*,4'*R*)-1,1''-dibenzyl-5''-chloro-5,7',7'-trimethyl-2,2'',5'-trioxo-1',4',5',6',7',8'-hexahydrodispiro[indoline-3,2'-quinoline-3',3''-indoline]-4'-carboxylate (3a): White solid, 85%, m.p. 259-260°C; ^{1}H NMR (400 MHz,$CDCl_3$) δ: 7.34 - 7.28 (m, 3H, ArH), 7.24 - 7.19 (m, 7H, ArH), 7.17 - 7.15 (m, 2H, ArH), 6.99 - 6.96 (m, 1H, ArH), 6.92 (d, J = 7.6 Hz, 1H, ArH), 6.38 (d, J = 8.0 Hz, 1H, ArH), 6.23 (d, J = 8.0 Hz, 1H, ArH), 4.94 (d, J = 16.0, 1H, CH_2), 4.87 (d, J = 15.6 Hz, 1H, CH_2), 4.80 (d, J = 15.2 Hz, 1H, CH_2), 4.79 (s, 1H, NH), 4.72 (d, J = 16.0 Hz, 1H, CH_2), 4.72 (s, 1H, CH), 3.93 - 3.85 (m, 1H, CH_2), 3.84 -3.76 (m, 1H, CH_2), 2.41 (s, 2H, CH_2), 2.35 (s, 2H, CH_2), 2.14 (s, 3H, CH_3), 1.30 (s, 3H, CH_3), 1.15 (s, 3H, CH_3), 0.77 (t, J = 7.2 Hz, 3H, CH_3) ppm; ^{13}C NMR (400 MHz, $CDCl_3$) δ: 197.5, 180.8, 177.1, 173.2, 142.8, 140.5, 137.9, 134.7, 134.4, 133.8, 131.2, 130.4, 129.7, 128.7, 128.7, 128.7, 128.6, 128.6, 127.6, 127.4, 127.1, 126.7, 126.5, 126.1, 125.9, 111.8, 110.0, 109.8, 87.3, 61.8, 53.7, 50.0, 44.6, 44.4, 43.6, 30.8, 29.8, 26.3, 21.0 ppm; IR (KBr) υ: 3526, 3052, 3025, 2997, 2983, 1843, 1765, 1623, 1524, 1422, 1283, 1246, 1055, 964, 841 cm^{-1}; HRMS (ESI-TOF) Calcd. for $C_{43}H_{38}ClN_3O_5$ ($[M+H]^+$): 714.2729, Found: 714.2718.

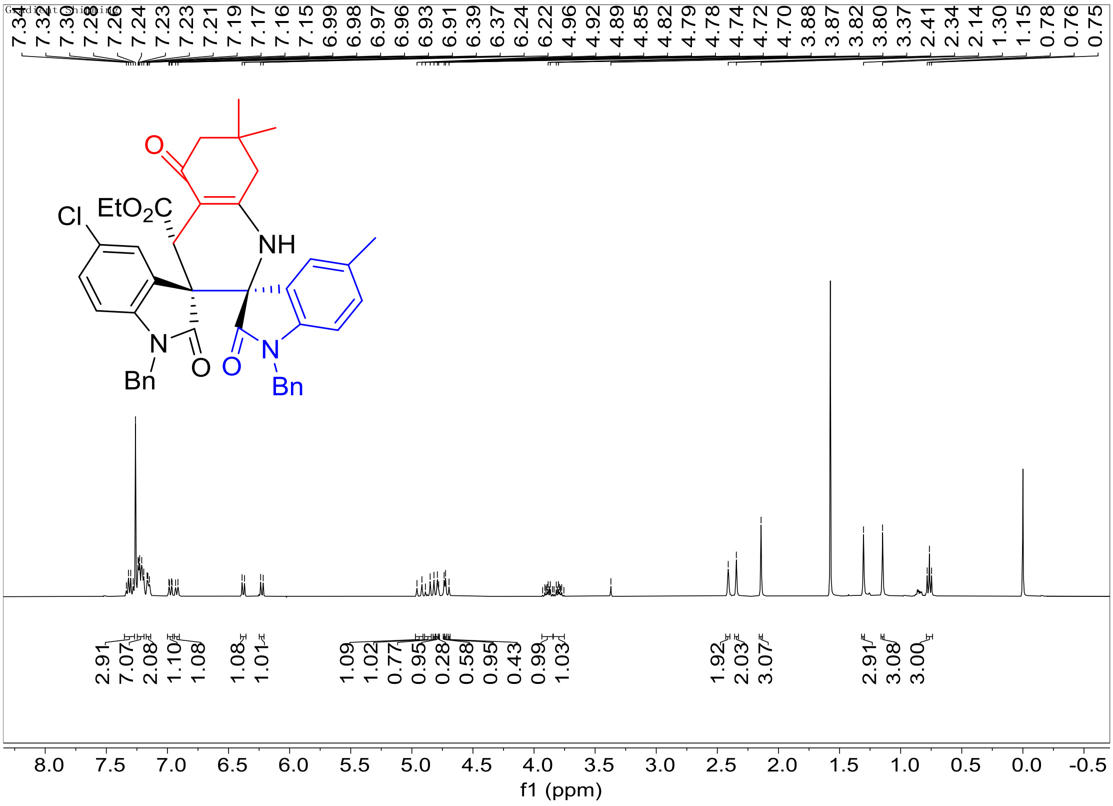

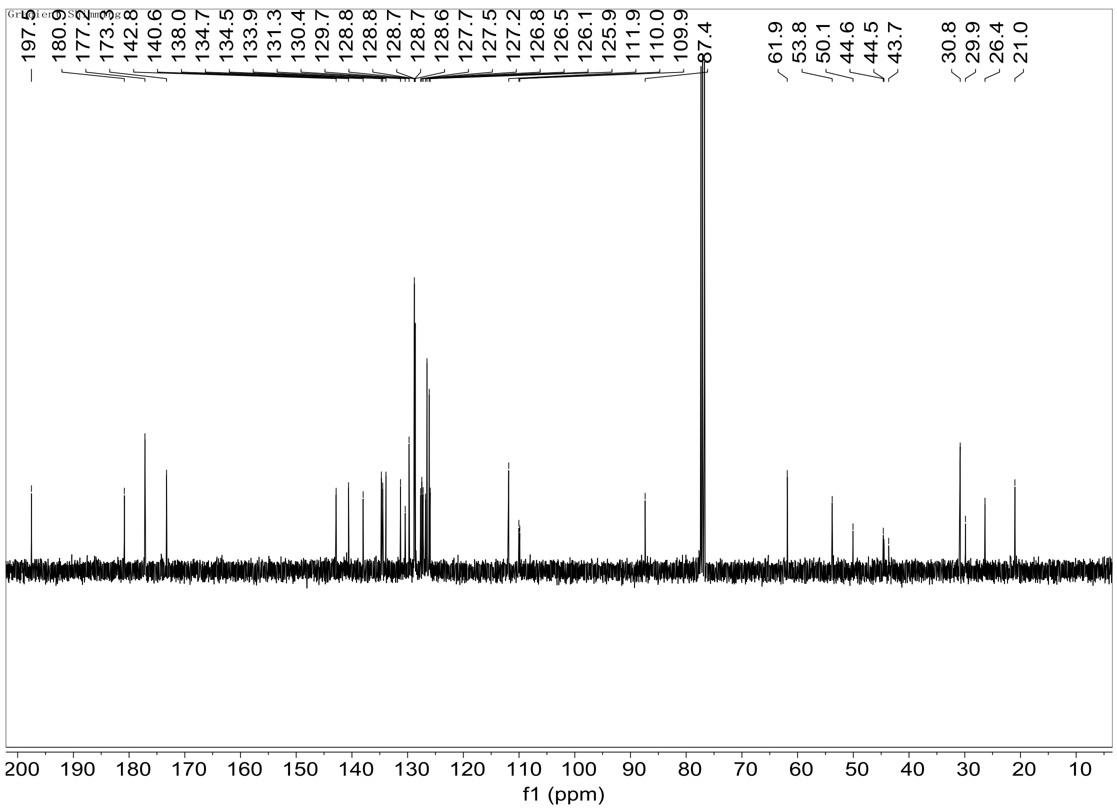
197.5
180.9
177.2
173.3
142.8
140.6
138.0
134.7
134.5
133.9
131.3
130.4
129.7
128.8
128.8
128.7
128.7
128.6
127.7
127.5
127.2
126.8
126.5
126.1
125.9
111.9
110.0
109.9
87.4
61.9
53.8
50.1
44.6
44.5
43.7
30.8
29.9
26.4
21.0
200 190 180 170 160 150 140 130 120 110 100 90 80 70 60 50 40 30 20 10
f1 (ppm)

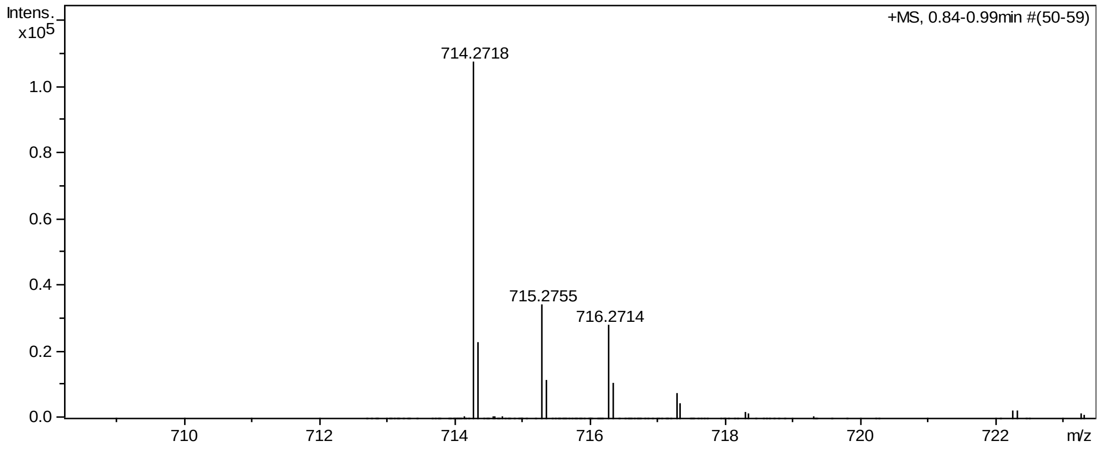
Intens.
x10^5
+MS, 0.84-0.99min #(50-59)
714.2718
715.2755
716.2714
1.0
0.8
0.6
0.4
0.2
0.0
710
712
714
716
718
720
722
m/z

Ethyl *rel*-(3*R*,3'*S*,4'*R*)-1,1''-dibenzyl-5,5''-dichloro-7',7'-dimethyl-2,2'',5'-trioxo-1',4',5',6',7',8'-hexahydrodispiro[indoline-3,2'-quinoline-3',3''-indoline]-4'-carboxylate (3b): White solid, 78%, m.p. 280–282°C; ^{1}H NMR (400 MHz,$CDCl_3$) δ: 7.43 (d, *J* = 2.0 Hz, 1H, ArH), 7.34 - 7.28 (m, 6H, ArH), 7.51 (s, 1H, ArH), 7.24 (s, 2H, ArH), 7.23 (s, 1H, ArH), 7.20 - 7.17 (m, 3H, ArH), 7.11 - 7.09 (m, 1H, ArH), 7.0 - 6.98 (m, 1H, ArH), 6.40 (d, *J* = 8.4 Hz, 1H, ArH), 6.26 (d, *J* = 8.4 Hz, 1H, ArH), 5.01 (d, *J* = 16.4 Hz, 1H, CH_2), 4.89 (d, *J* = 15.6 Hz, 1H, CH_2), 4.78 (d, *J* = 15.6 Hz, 1H, CH_2), 4.76 (s, 1H, NH), 4.74 (s, 1H, CH), 4.66 (d, *J* = 16.4 Hz, 1H, CH_2), 3.94 - 3.87 (m, 1H, CH_2), 3.84 -3.77 (m, 1H, CH_2), 2.46 (d, *J* = 16.0 Hz, 1H, CH_2), 2.39 (d, *J* = 16.0 Hz,1H, CH_2), 2.37 - 2.34 (m, 2H, CH_2), 1.30 (s, 3H, CH_3), 1.15 (s, 3H, CH_3), 0.76 (t, *J* = 7.2 Hz, 3H, CH_3) ppm; ^{13}C NMR (400 MHz, $CDCl_3$) δ: 193.0, 173.8, 171.9, 171.2, 155.6, 142.0, 141.8, 134.8, 134.1, 131.0, 129.3, 129.1, 128.8, 128.7, 128.6, 127.9, 127.5, 127.5, 127.1, 127.1, 127.0, 126.2, 125.9, 125.6, 124.9, 110.9, 110.3, 102.3, 62.3, 60.3, 50.0, 49.4, 44.4, 44.2, 42.5, 42.4, 32.9, 29.0, 27.6, 13.5 ppm; IR (KBr) υ: 3504, 3024, 3010, 2995, 2985, 1847, 1711, 1603, 1517, 1400, 1299, 1250, 1053, 953, 841 cm^{-1}; HRMS (ESI-TOF) Calcd. for $C_{42}H_{37}Cl_2N_3O_5$ ([M+Na]$^+$): 756.2002, Found: 756.1989.

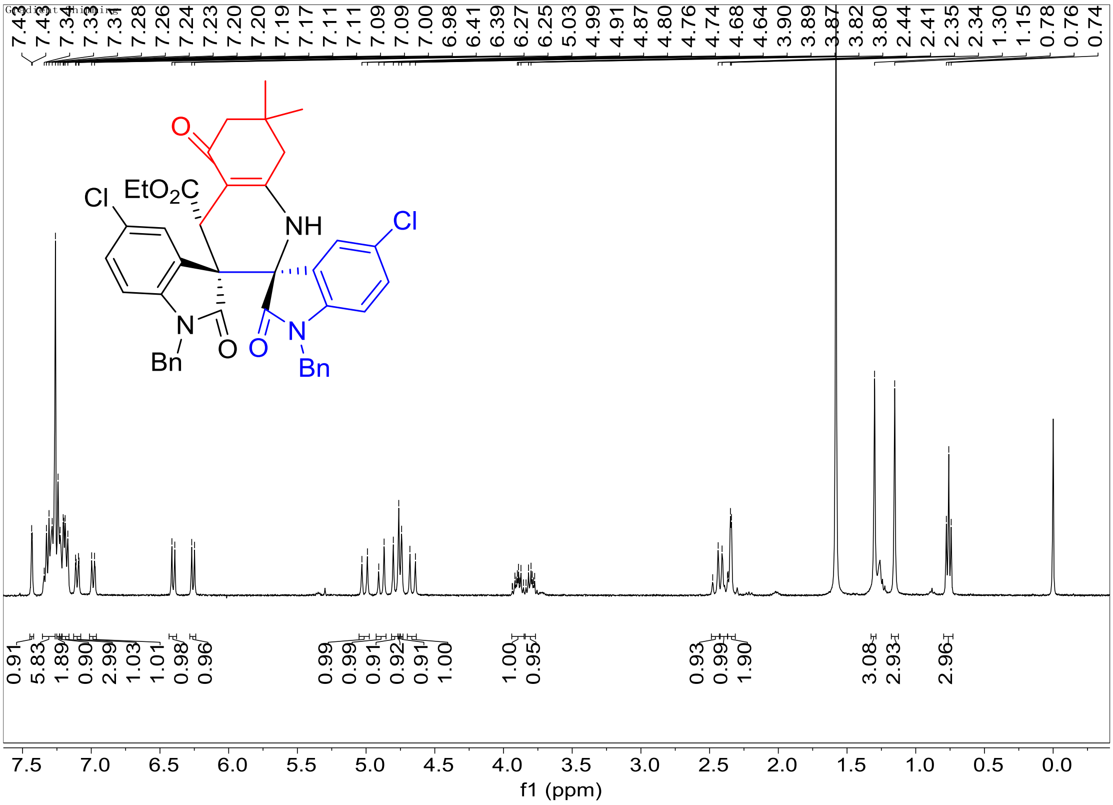

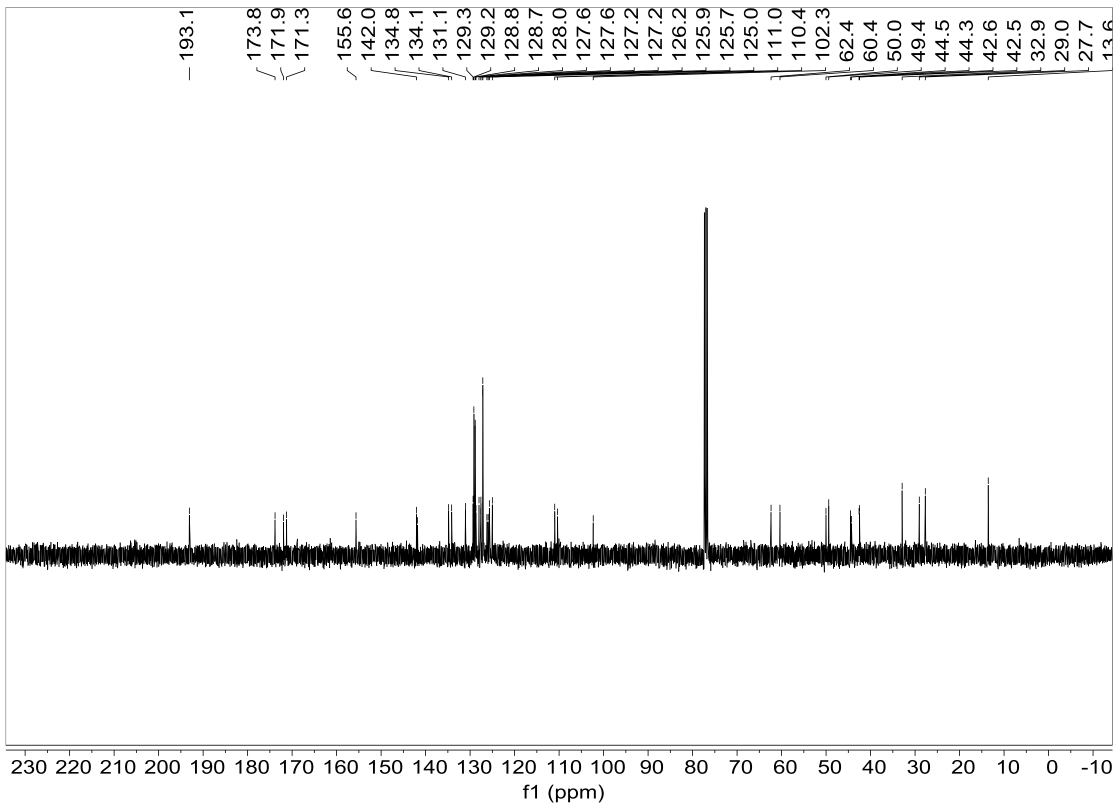
193.1
173.8
171.9
171.3
155.6
142.0
134.8
134.1
131.1
129.3
129.2
128.8
128.7
128.0
127.6
127.6
127.2
127.2
126.2
125.9
125.7
125.0
111.0
110.4
102.3
62.4
60.4
50.0
49.4
44.5
44.3
42.6
42.5
32.9
29.0
27.7
230 220 210 200 190 180 170 160 150 140 130 120 110 100 90 80 70 60 50 40 30 20 10 0 -10
f1 (ppm)

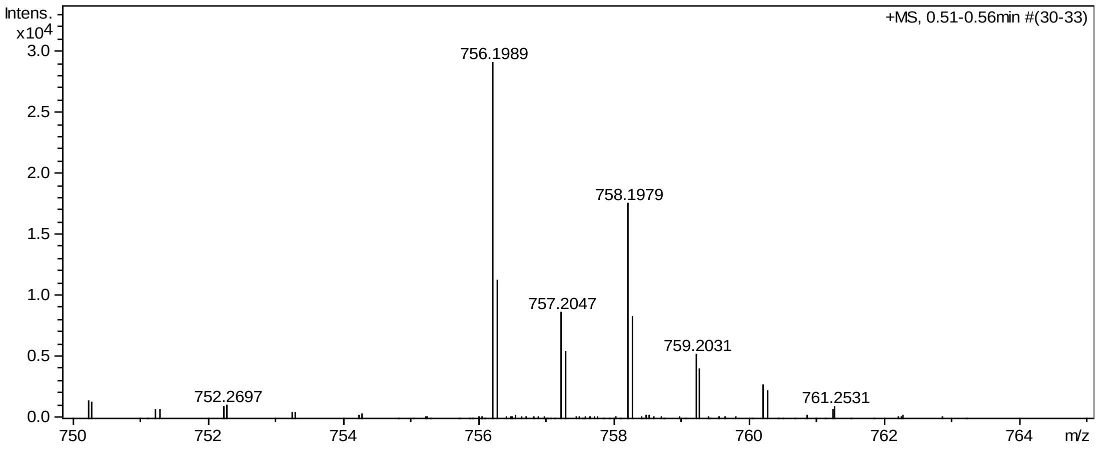
Intens.
x10^4
+MS, 0.51-0.56min #(30-33)
756.1989
758.1979
757.2047
759.2031
752.2697
761.2531
3.0
2.5
2.0
1.5
1.0
0.5
0.0
750 752 754 756 758 760 762 764 m/z

Ethyl *rel*-(3*R*,3'*S*,4'*R*)-1''-benzyl-1-butyl-5,5''-dichloro-7',7'-dimethyl-2,2'',5'-trioxo-1',4',5',6',7',8'-hexahydrodispiro[indoline-3,2'-quinoline-3',3''-indoline]-4'-carboxylate (3c): White solid, 82%, m.p. 220-221°C; ^{1}H NMR (400 MHz,$CDCl_3$) δ: 7.41 (d, *J* = 2.0 Hz, 1H, ArH), 7.33 - 7.28 (m, 2H, ArH), 7.23 - 7.21 (m, 4H, ArH), 7.12 (d, *J* = 2.0 Hz, 1H, 2H, ArH), 6.95 - 6.92 (m, 1H, ArH), 6.56 (d, *J* = 8.4 Hz, 1H, ArH), 6.21 (d, *J* = 8.4 Hz, 1H, ArH), 5.02 (d, *J* = 16.0 Hz, 1H, CH_2), 4.71 (s, 1H, NH), 4.68 (s, 1H, CH), 4.62 (d, *J* = 16.0 Hz, 1H, CH_2), 3.93 - 3.83 (m, 1H, CH_2), 3.81 - 3.72 (m, 2H, CH_2), 3.45 - 3.38 (m, 1H, CH_2), 2.38 - 2.28 (m, 3H, CH_2), 1.70 - 1.63 (m, 1H, CH_2), 1.42 - 1.35 (m, 2H, CH_2), 1.54 - 1.48 (m, 1H, CH_2), 1.28 (s, 3H, CH_3), 1.14 (s, 3H, CH_3), 0.99 (t, *J* = 7.2 Hz, 3H, CH_3), 0.74 (t, *J* = 6.8 Hz, 3H, CH_3) ppm; ^{13}C NMR (400 MHz, $CDCl_3$) δ: 234.3, 192.9, 173.3, 171.8, 171.2, 155.5, 142.0, 141.8, 134.8, 131.0, 129.1, 128.7, 128.3, 127.5, 127.3, 127.1, 125.9, 125.6, 124.9, 110.1, 109.7, 102.2, 62.1, 60.3, 50.0, 49.4, 44.1, 42.5, 42.3, 40.4, 40.4, 32.9, 29.3, 28.9, 27.7, 20.3, 13.7, 13.5 ppm; IR (KBr) υ: 3533, 3069, 3012, 2993, 2869, 1862, 1726, 1608, 1516, 1463, 1526, 1276, 1224, 1053, 962, 856 cm^{-1}; HRMS (ESI-TOF) Calcd. for $C_{39}H_{39}ClN_3O_5$ ($[M+Na]^+$): 722.2159, Found: 722.2152.

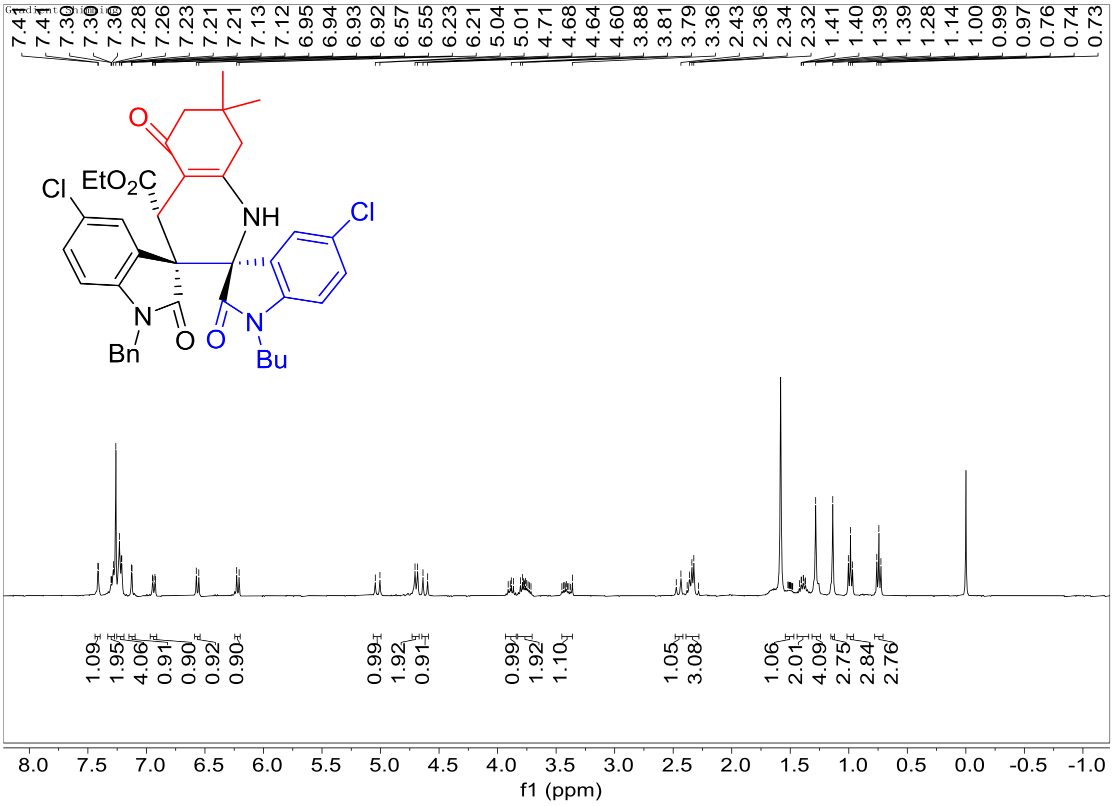

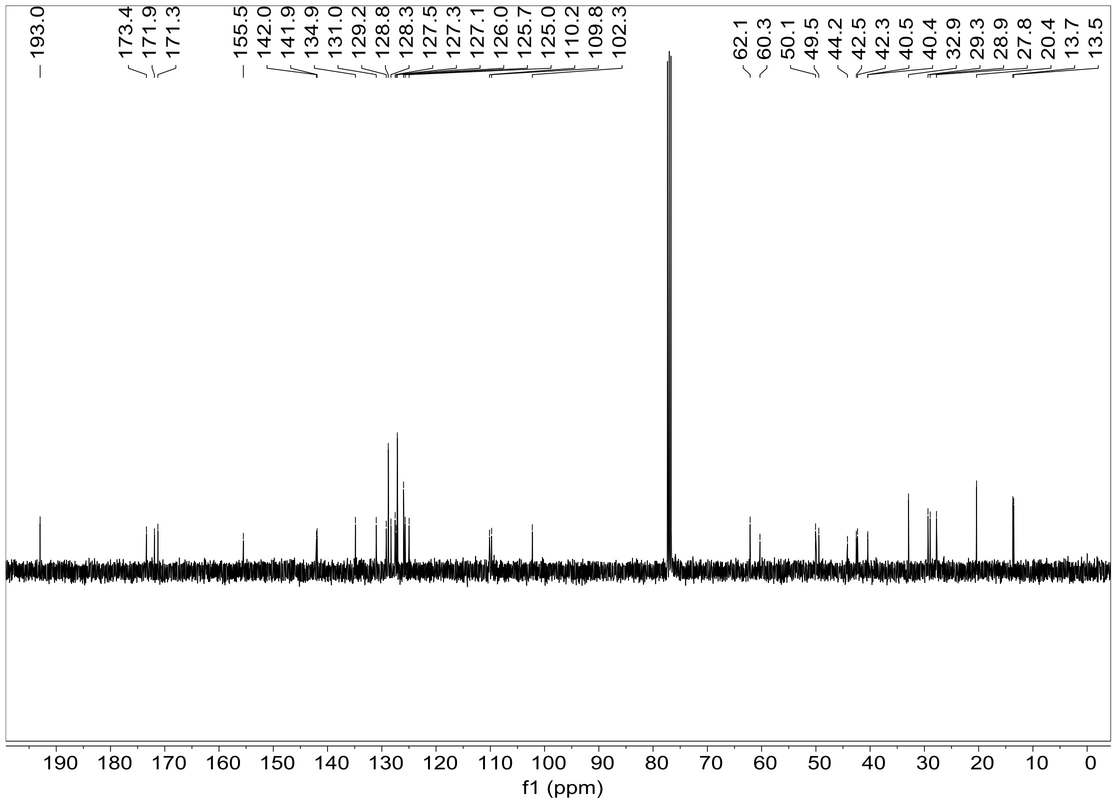
193.0
173.4
171.9
171.3
155.5
142.0
141.9
134.9
131.0
129.2
128.8
128.3
127.5
127.3
127.1
126.0
125.7
125.0
110.2
109.8
102.3
62.1
60.3
50.1
49.5
44.2
42.5
42.3
40.5
40.4
32.9
29.3
28.9
27.8
20.4
13.7
13.5
190 180 170 160 150 140 130 120 110 100 90 80 70 60 50 40 30 20 10 0
f1 (ppm)

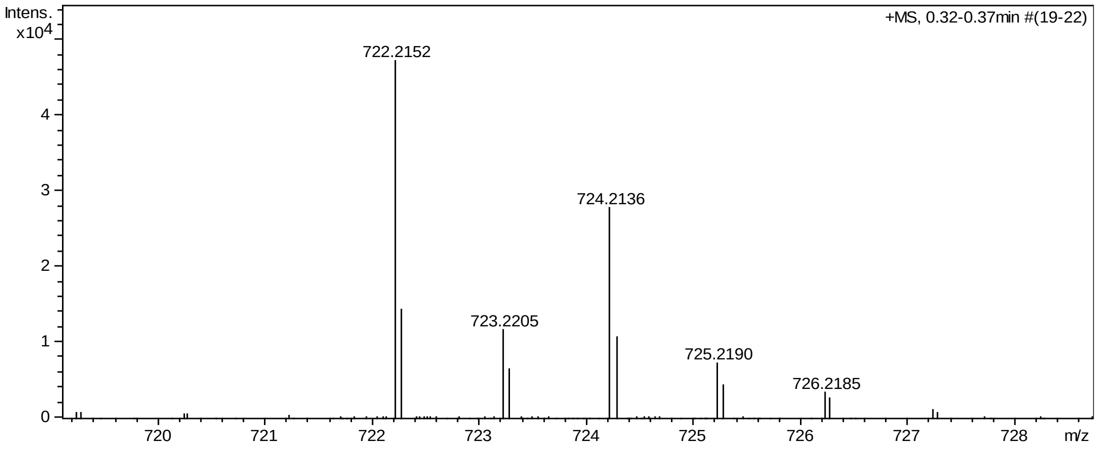
Intens.
x10^4
+MS, 0.32-0.37min #(19-22)
722.2152
723.2205
724.2136
725.2190
726.2185
720 721 722 723 724 725 726 727 728 m/z

Ethyl *rel*-(3*R*,3'*S*,4'*R*)-1''-benzyl-1-butyl-5''-chloro-5,7',7'-trimethyl-2,2'',5'-trioxo-1',4',5',6',7',8'-hexahydrodispiro[indoline-3,2'-quinoline-3',3''-indoline]-4'-carboxylate (3d): White solid, 81%, m.p. 263-264°C; ^{1}H NMR (400 MHz,$CDCl_3$) δ: 7.23 (s, 3H, ArH), 7.19 - 7.15 (m, 4H, ArH), 7.04 (d, *J* = 7.6 Hz, 1H, ArH), 6.93 (d, *J* = 8.0 Hz, 1H, ArH), 6.53 (d, *J* = 8.0 Hz, 1H, ArH), 6.18 (d, *J* = 8.0 Hz, 1H, ArH), 4.94 (d, *J* = 16.0 Hz, 1H, CH_2), 4.74 (s, 1H, NH), 4.70 (s, 1H, CH), 4.18 (d, *J* = 16.4 Hz, 1H, CH_2), 3.92 - 3.84 (m, 1H, CH_2), 3.82 - 3.72 (m, 2H, CH_2), 3.45 - 3.38 (m, 1H, CH_2), 2.42 (d, *J* = 15.2 Hz, 1H, CH_2), 2.37 - 2.33 (m, 3H, CH_2), 2.18 (s, 3H, CH_3), 1.68 - 1.65 (m, 1H, CH_2), 0.99 (t, *J* = 7.2 Hz, 3H, CH_3), 0.75 (t, *J* = 7.2 Hz, 3H, CH_3) ppm; ^{13}C NMR (400 MHz, $CDCl_3$) δ: 192.7, 173.6, 172.1, 171.4, 155.5, 141.9, 141.0, 135.0, 132.6, 131.2, 128.9, 128.6, 127.3, 127.2, 126.9, 126.4, 125.9, 125.0, 124.3, 109.9, 109.9, 109.8, 108.5, 108.5, 102.1, 62.2, 60.1, 50.0, 49.5, 43.9, 42.6, 42.5, 42.4, 40.3, 32.8, 29.4, 29.0, 27.7, 20.9, 20.9, 20.4, 13.7, 13.5 ppm; IR (KBr) υ: 3504, 3063, 3002, 2990, 2986, 1822, 1763, 1646, 1521, 1402, 1282, 1254, 1023, 989, 852 cm^{-1}; HRMS (ESI-TOF) Calcd. for $C_{40}H_{43}ClN_3O_5$ ($[M+H]^+$): 680.2886, Found: 680.2878.

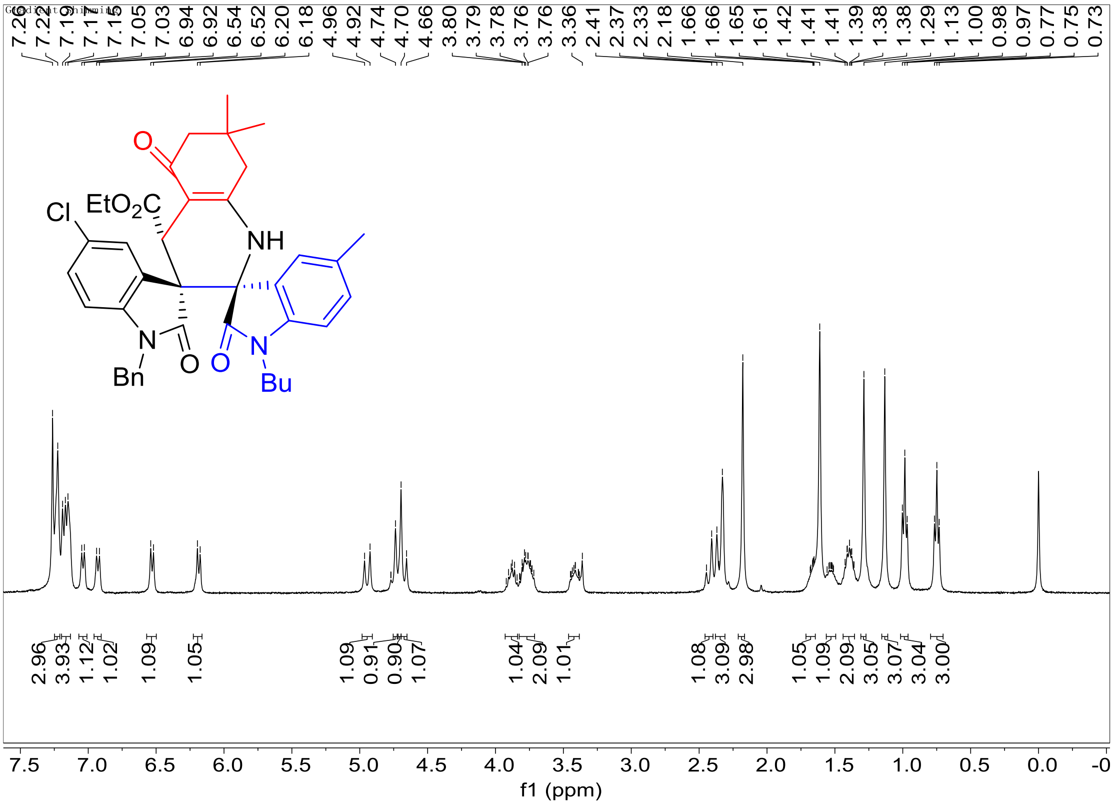

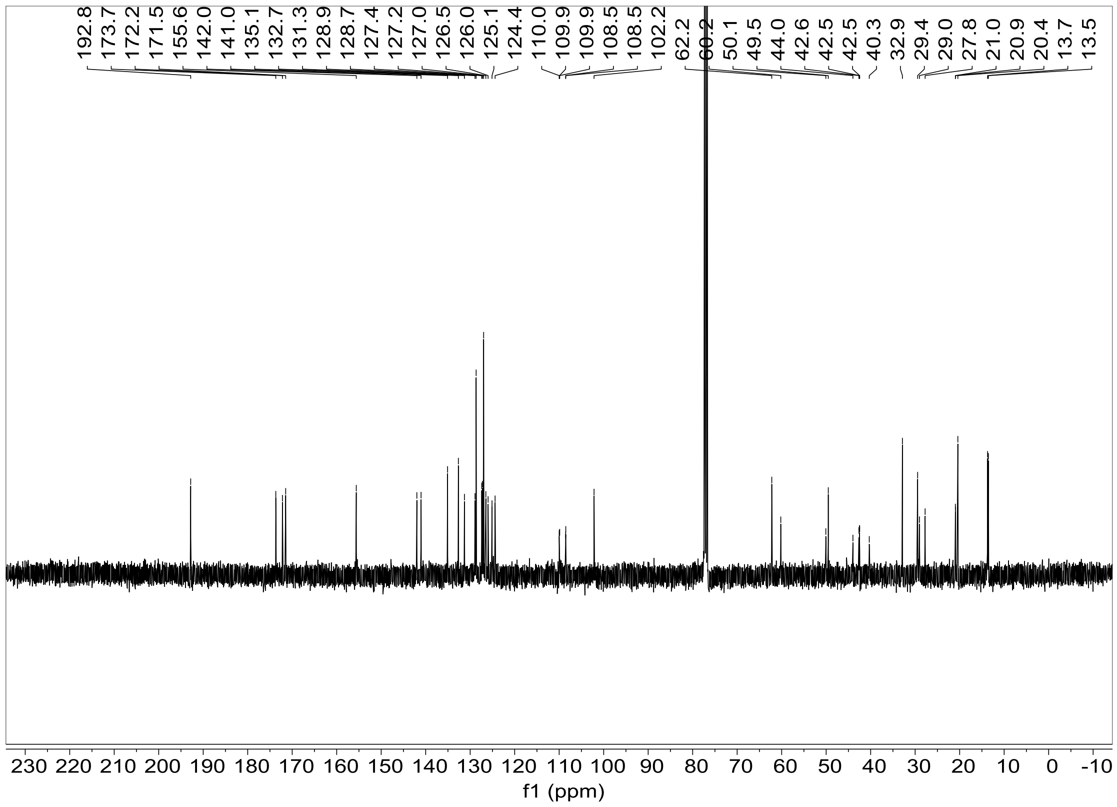
192.8 173.7 172.2 171.5 155.6 142.0 141.0 135.1 132.7 131.3 128.9 128.7 127.4 127.2 127.0 126.5 126.0 125.1 124.4 110.0 109.9 109.9 108.5 108.5 102.2 62.2 60.2 50.1 49.5 44.0 42.6 42.5 42.5 40.3 32.9 29.4 29.0 27.8 21.0 20.9 20.4 13.7 13.5
230 220 210 200 190 180 170 160 150 140 130 120 110 100 90 80 70 60 50 40 30 20 10 0 -10
f1 (ppm)

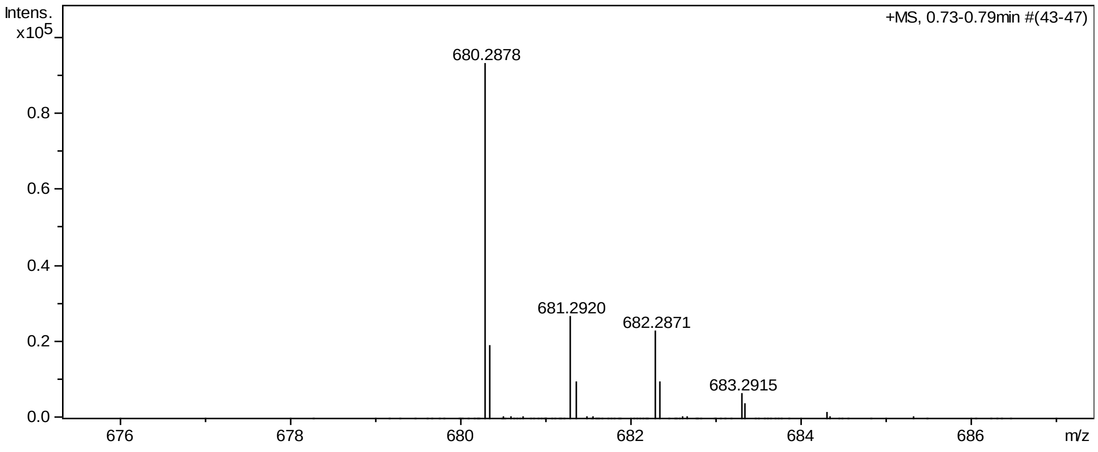
Intens.
x10^5
+MS, 0.73-0.79min #(43-47)
680.2878
681.2920
682.2871
683.2915
0.8
0.6
0.4
0.2
0.0
676 678 680 682 684 686 m/z

Ethyl ***rel*-(3*R*,3'*S*,4'*R*)-1''-benzyl-5''-chloro-7',7'-dimethyl-2,2'',5'-trioxo-1',4',5',6',7',8'-hexahydrodispiro[indoline-3,2'-quinoline-3',3''-indoline]-4'-carboxylate (3e):** White solid, 69%, m.p. 221-222°C; ^{1}H NMR (400 MHz,CDCl$_3$) δ: 7.73 (s, 1H, NH), 7.42 (s, 2H, ArH), 7.25 (s, 2H, ArH), 7.23 (s, 1H, ArH), 7.21 - 7.19 (m, 2H, ArH), 7.15 (d, *J* = 7.6 Hz, 1H, ArH), 6.98 - 6.96 (m, 1H, ArH), 6.80 (t, *J* = 7.6 Hz, 1H, ArH), 6.67 (d, *J* = 7.6 Hz, 1H, ArH), 6.28 (d, *J* = 8.4 Hz, 1H, ArH), 4.96 (d, *J* = 15.6 Hz, 1H, CH$_2$), 4.81 (s, 1H, NH), 4.66 (s, 1H, CH), 4.61 (d, *J* = 16.0 Hz, 1H, CH$_2$), 3.92 - 3.84 (m, 1H, CH$_2$), 3.82 - 3.74 (m, 1H, CH$_2$), 2.49 (d, *J* = 16.4Hz, 1H, CH$_2$), 2.37 (d, *J* = 16.4 Hz, 1H, CH$_2$), 2.32 - 2.28 (m, 2H, CH$_2$), 1.26 (s, 3H, CH$_3$), 1.13 (s, 3H, CH$_3$), 0.75 (t, *J* = 7.2 Hz, 3H, CH$_3$) ppm; ^{13}C NMR (400 MHz, CDCl$_3$) δ: 190.7, 176.0, 171.4, 171.1, 157.3, 142.5, 142.3, 136.1, 130.9, 129.1, 128.7, 128.0, 127.7, 127.1, 125.9, 125.8, 124.8, 124.3, 122.2, 99.9, 62.4, 59.4, 50.3, 49.1, 42.2, 32.7, 29.1, 27.8, 13.7 ppm; IR (KBr) υ: 3526, 3052, 3012, 2996, 2981, 2853, 1868, 1766, 1609, 1499, 1426, 1258, 1225, 1006, 982, 841 cm^{-1}; HRMS (ESI-TOF) Calcd. for $C_{35}H_{33}ClN_3O_5$ ([M+H]$^+$): 610.2103, Found: 610.2105.

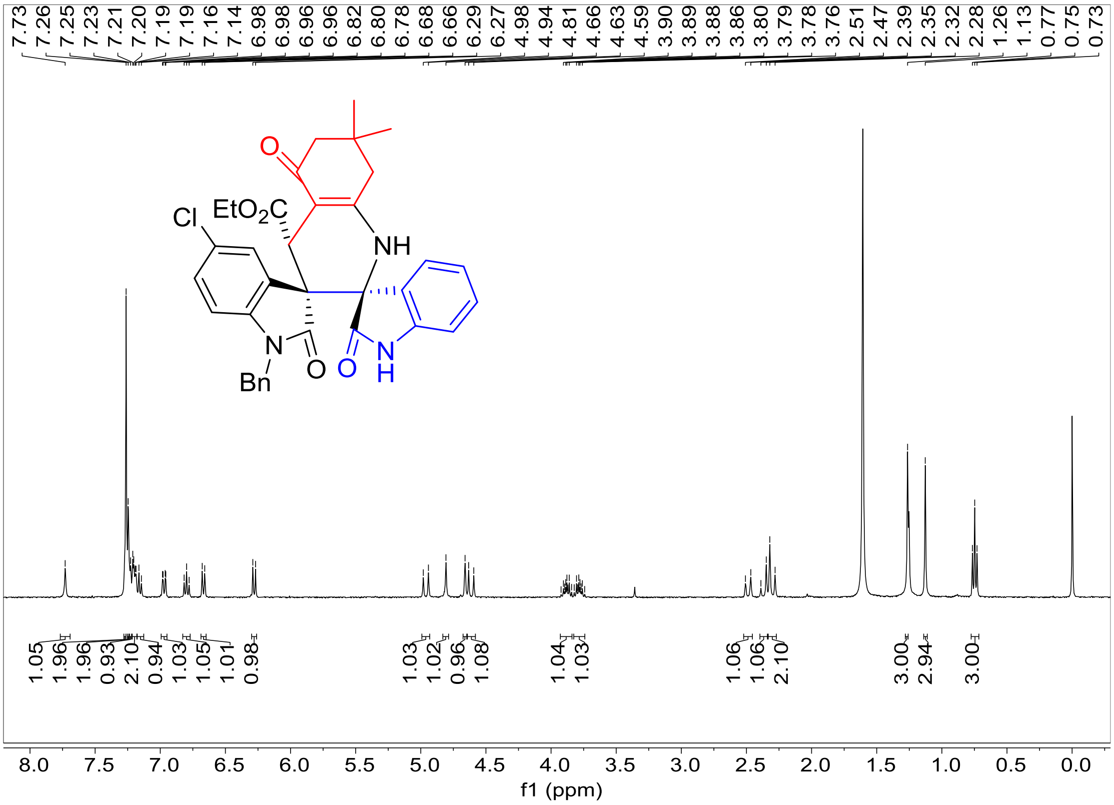

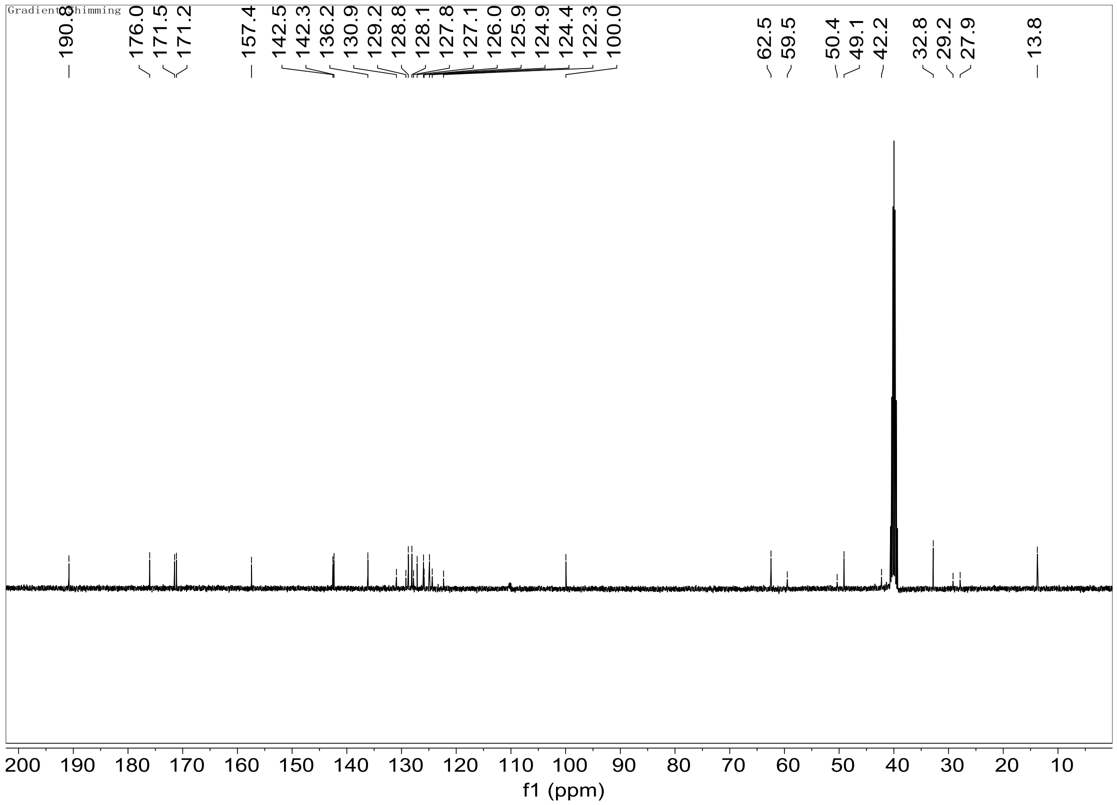

Gradient Shimming
190.8
176.0
171.5
171.2
157.4
142.5
142.3
136.2
130.9
129.2
128.8
128.1
127.8
127.1
126.0
125.9
124.9
124.4
122.3
100.0
62.5
59.5
50.4
49.1
42.2
32.8
29.2
27.9
13.8
200 190 180 170 160 150 140 130 120 110 100 90 80 70 60 50 40 30 20 10
f1 (ppm)

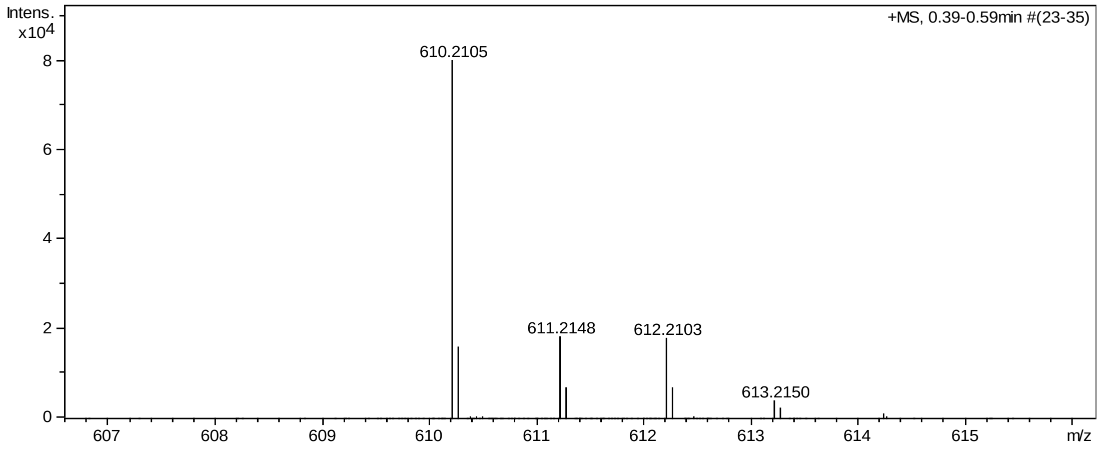

Intens.
x10^4
+MS, 0.39-0.59min #(23-35)
610.2105
611.2148
612.2103
613.2150
8
6
4
2
0
607 608 609 610 611 612 613 614 615 m/z

Ethyl *rel*-(3*R*,3'*S*,4'*R*)-1''-benzyl-5''-chloro-5,7',7'-trimethyl-2,2'',5'-trioxo-1',4',5',6',7',8'-hexahydrodispiro[indoline-3,2'-quinoline-3',3''-indoline]-4'-carboxylate (3f): White solid, 70%, m.p. 230–231°C; ^{1}H NMR (400 MHz,$CDCl_3$) δ: 7.49 (s, 1H, NH), 7.28 (d, *J* = 2.0 Hz, 1H, ArH), 7.24 (s, 1H, ArH), 7.23 (s, 2H, ArH), 7.17 - 7.14 (m, 3H, ArH), 7.01 - 6.99 (m, 1H, ArH), 6.97 - 6.94 (m, 1H, ArH), 6.58 (d, *J* = 7.6 Hz, 1H, ArH), 6.21 (d, *J* = 8.4 Hz, 1H, ArH), 4.95 (d, *J* = 16.0 Hz, 1H, CH_2), 4.74 (s, 1H, NH), 4.68 (d, *J* = 15.2 Hz, 1H, CH_2), 4.67 (s, 1H, CH), 4.76 (s, 1H, NH), 4.74 (s, 1H, CH), 4.66 (d, *J* = 16.4 Hz, 1H, CH_2), 3.94 - 3.87 (m, 1H, CH), 3.93 -3.84 (m, 1H, CH_2), 3.82 - 3.76 (m, 1H, CH_2), 2.46 (d, *J* = 16.0 Hz,1H, CH_2), 2.39 - 2.29 (m, 3H, CH_2), 2.17 (s, 3H, CH_3), 1.28 (s, 3H, CH_3), 1.13 (s, 3H, CH_3),0.75 (t, *J* = 7.2 Hz, 3H, CH_3) ppm; ^{13}C NMR (400 MHz, $CDCl_3$) δ: 190.7, 176.0, 171.5, 171.1, 157.4, 142.5, 139.8, 136.1, 131.3, 129.1, 128.8, 128.7, 127.6, 127.1, 126.3, 126.0, 125.1, 124.4, 99.8, 62.5, 59.4, 49.2, 42.3, 32.7, 29.2, 27.8, 21.1, 21.0, 13.7 ppm; IR (KBr) υ: 3608, 3589, 3052, 3007, 2991, 2982, 1866, 1739, 1613, 1509, 1425, 1276, 1242, 1024, 987, 856 cm^{-1}; HRMS (ESI-TOF) Calcd. for $C_{36}H_{35}ClN_3O_5$ ($[M+H]^+$): 624.2260, Found: 624.2262.

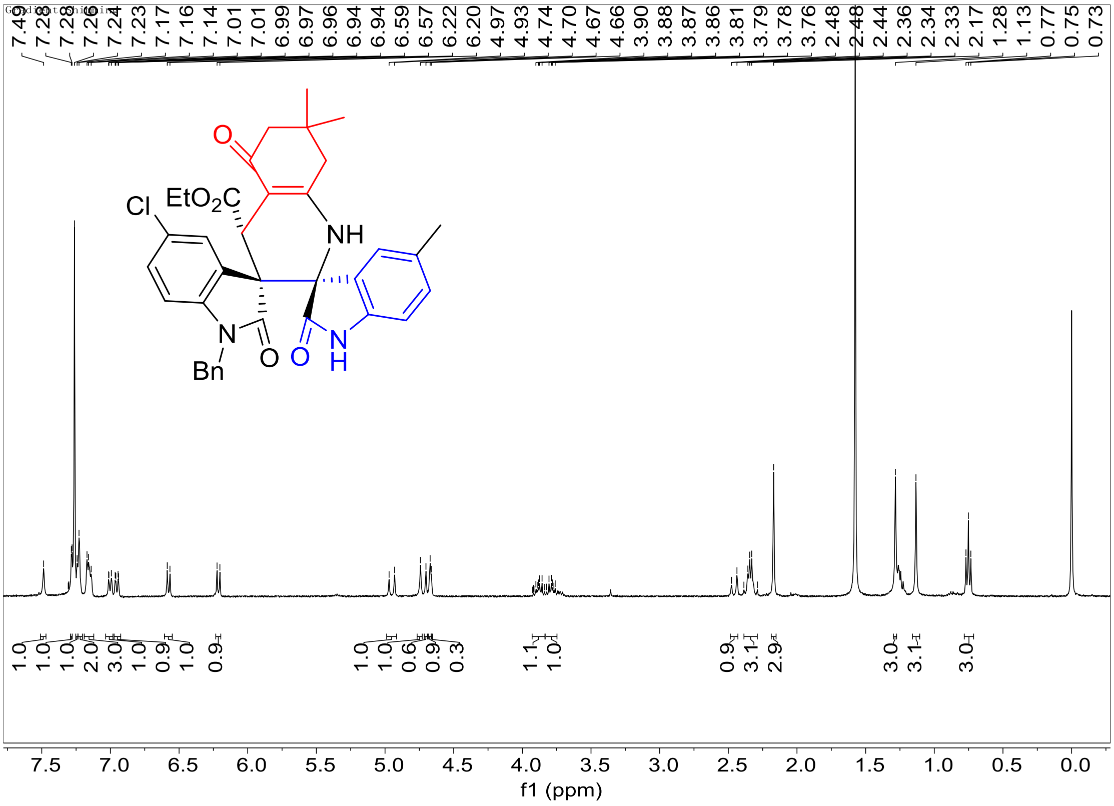

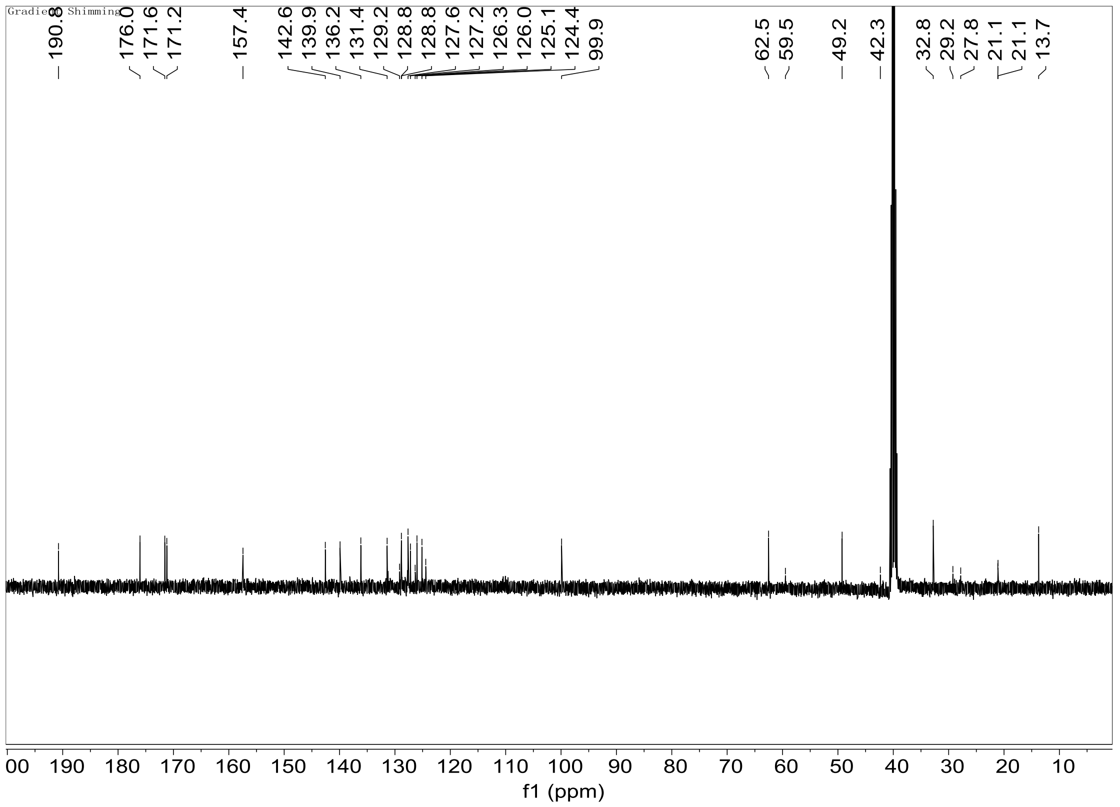
Gradient Shimming
190.8
176.0
171.6
171.2
157.4
142.6
139.9
136.2
131.4
129.2
128.8
128.8
127.6
127.2
126.3
126.0
125.1
124.4
99.9
62.5
59.5
49.2
42.3
32.8
29.2
27.8
21.1
21.1
13.7
00 190 180 170 160 150 140 130 120 110 100 90 80 70 60 50 40 30 20 10
f1 (ppm)

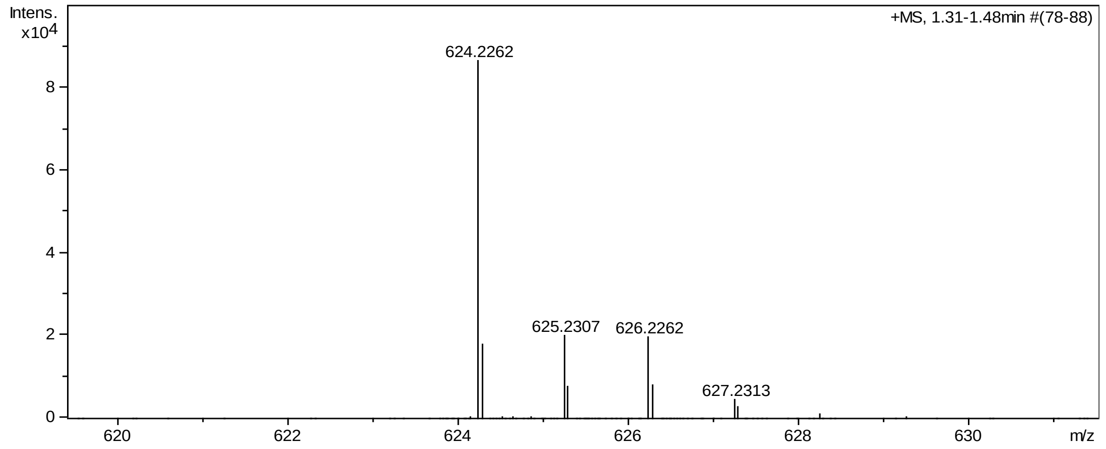
Intens.
x10^4
+MS, 1.31-1.48min #(78-88)
624.2262
625.2307
626.2262
627.2313
8
6
4
2
0
620
622
624
626
628
630
m/z

Ethyl *rel*-(3*R*,3'*S*,4'*R*)-1''-benzyl-5,5''-dichloro-7',7'-dimethyl-2,2'',5'-trioxo-1',4',5',6',7',8'-hexahydrodispiro[indoline-3,2'-quinoline-3',3''-indoline]-4'-carboxylate (3g): White solid, 72%, m.p. 234-235°C; ^{1}H NMR (400 MHz,$CDCl_3$) δ: 7.61 (s, 1H, NH),7.40 (s, 1H, ArH), 7.31 - 7.28 (m, 2H, ArH), 7.24 (d, *J* = 5.2 Hz, 3H, ArH), 7.21 - 7.18 (m, 2H, ArH), 6.96 (d, *J* = 9.2 Hz, 1H, ArH), 6.63 (d, *J* = 8.4 Hz, 1H, ArH), 6.25 (d, *J* = 8.0 Hz, 1H, ArH), 5.02 (d, *J* = 16.0 Hz, 1H, CH_2), 4.78 (s, 1H, NH), 4.63 (d, *J* = 15.6 Hz, 1H, CH_2), 4.62 (s, 1H, CH), 3.91 - 3.85 (m, 1H, CH_2), 3.82 - 3.76 (m, 1H, CH_2), 2.49 (d, *J* = 16.0 Hz, 1H, CH_2), 2.39 - 2.31 (m, 3H, CH_2), 1.28 (s, 3H, CH_3), 1.14 (s, 3H, CH_3), 0.75 (t, *J* = 7.2 Hz, 3H, CH_3) ppm; ^{13}C NMR (400 MHz, $CDCl_3$) δ: 234.3, 180.5, 171.5, 140.9, 134.6, 129.8, 129.0, 128.8, 128.7, 128.2, 127.9, 127.4, 127.3, 124.8, 113.0, 110.5, 61.5, 49.5, 44.6, 39.2, 31.4, 31.0, 13.9 ppm; R (KBr) υ: 3612, 3522, 3061, 3015, 2998, 2986, 1852, 1776, 1609, 1529, 1416, 1256, 1221, 1045, 985, 823, 752 cm^{-1}; HRMS (ESI-TOF) Calcd. for $C_{35}H_{32}Cl_2N_3O_5$ ([M+H]$^+$): 644.1714, Found: 644.1738.

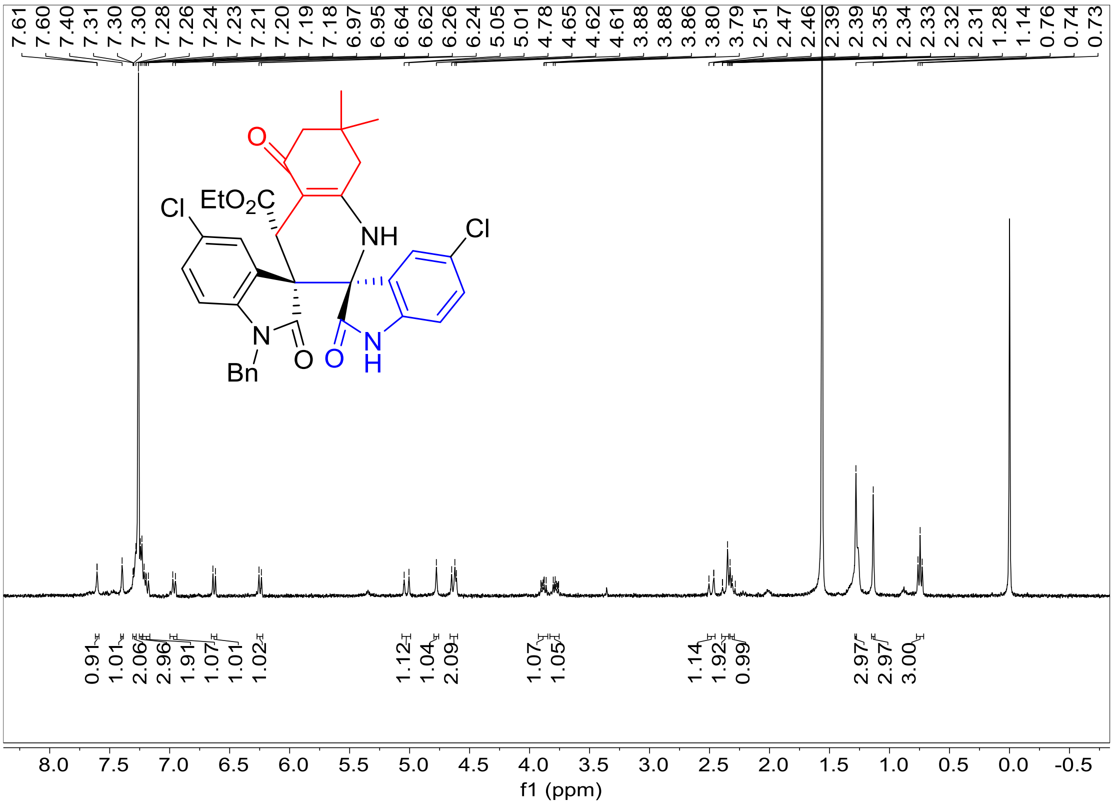

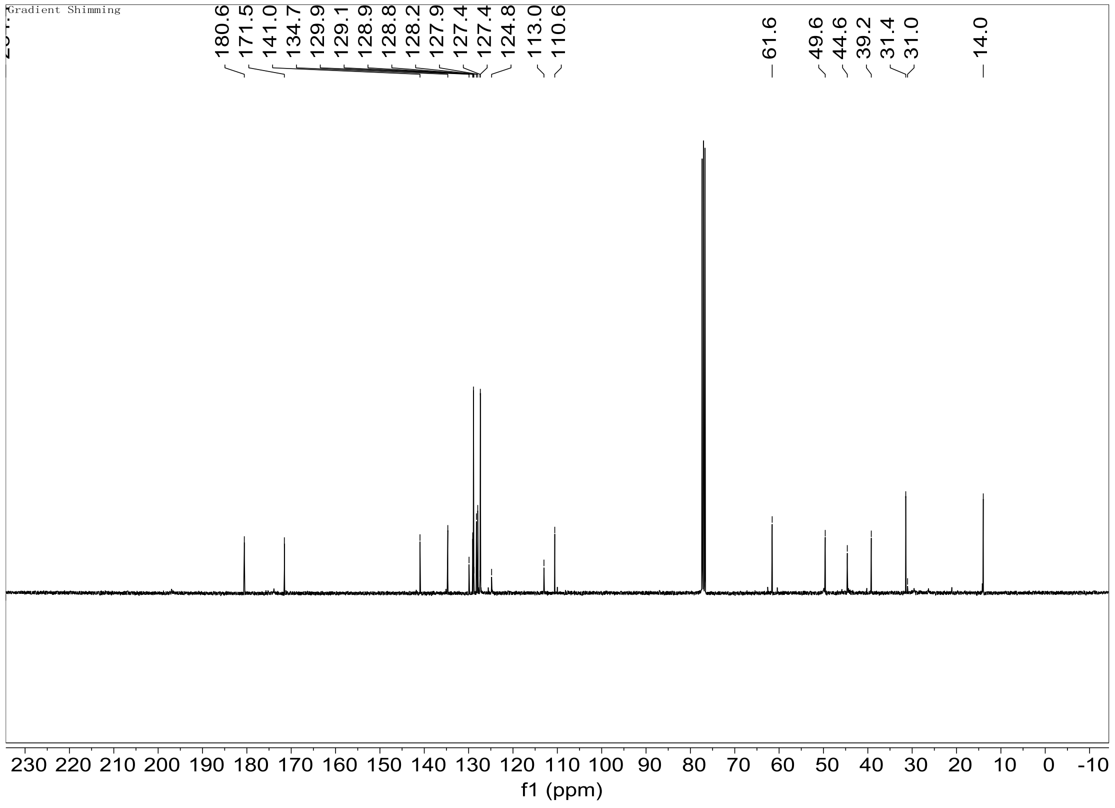
Gradient Shimming
180.6
171.5
141.0
134.7
129.9
129.1
128.9
128.8
128.2
127.9
127.4
127.4
124.8
113.0
110.6
61.6
49.6
44.6
39.2
31.4
31.0
14.0
230 220 210 200 190 180 170 160 150 140 130 120 110 100 90 80 70 60 50 40 30 20 10 0 -10
f1 (ppm)

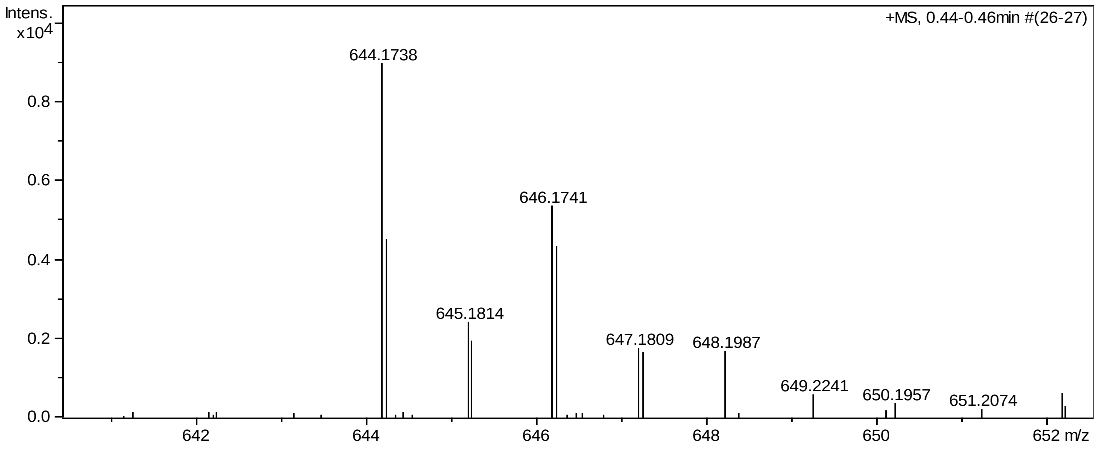
Intens.
x10^4
+MS, 0.44-0.46min #(26-27)
644.1738
646.1741
645.1814
647.1809
648.1987
649.2241
650.1957
651.2074
0.8
0.6
0.4
0.2
0.0
642
644
646
648
650
652 m/z

Ethyl *rel*-(3*R*,3'*S*,4'*R*)-1''-benzyl-5''-chloro-5-fluoro-7',7'-dimethyl-2,2'',5'-trioxo-1',4',5',6',7',8'-hexahydrodispiro[indoline-3,2'-quinoline-3',3''-indoline]-4'-carboxylate (3h): White solid, 70%, m.p. 240-241ºC; ^{1}H NMR (400 MHz,$CDCl_3$) δ: 7.72 (s, 1H, NH), 7.26 (s, 3H, ArH), 7.23 - 7.21 (m, 3H, ArH), 7.12 -7.10 (m, 1H, ArH), 7.12 - 7.10 (m, 1H, ArH), 6.99 - 6.97 (m, 1H, ArH), 6.93 - 6.88 (m, 1H, ArH), 6.65 - 6.62 (m, 1H, ArH), 6.29 (d, *J* = 8.4 Hz, 1H, ArH), 4.98 (d, *J* = 15.2 Hz, 1H, CH_2), 4.81 (s, 1H, NH), 4.65 (d, *J* = 14.4 Hz, 1H, CH_2), 4.63 (s, 1H, CH), 3.90 - 3.84 (m, 1H, CH_2), 3.82 - 3.76 (m, 1H, CH_2), 2.49 (d, *J* = 15.6 Hz, 1H, CH_2), 2.35 - 2.28 (m, 3H, CH_2), 1.27 (s, 3H, CH_3), 1.13 (s, 3H, CH_3), 0.75 (t, *J* = 6.8 Hz, 3H, CH_3) ppm; ^{13}C NMR (400 MHz, $CDCl_3$) δ: 190.9, 176.0, 171.3, 170.9, 159.2, 157.1, 156.8, 142.4, 138.6, 138.6, 135.9, 129.4, 128.7, 127.7, 127.7, 126.7, 126.6, 126.5, 126.2, 124.4, 99.9, 62.5, 59.5, 49.1, 42.3, 42.3, 32.8, 29.1, 27.8, 13.7 ppm; IR (KBr) υ: 3613, 3526, 3023, 3011, 1856, 1726, 1612, 1544, 1462, 1275, 1223, 1062, 956, 893 cm^{-1}; HRMS (ESI-TOF) Calcd. for $C_{35}H_{32}ClFN_3O_5$ ($[M+H]^+$): 628.2009, Found: 628.2011.

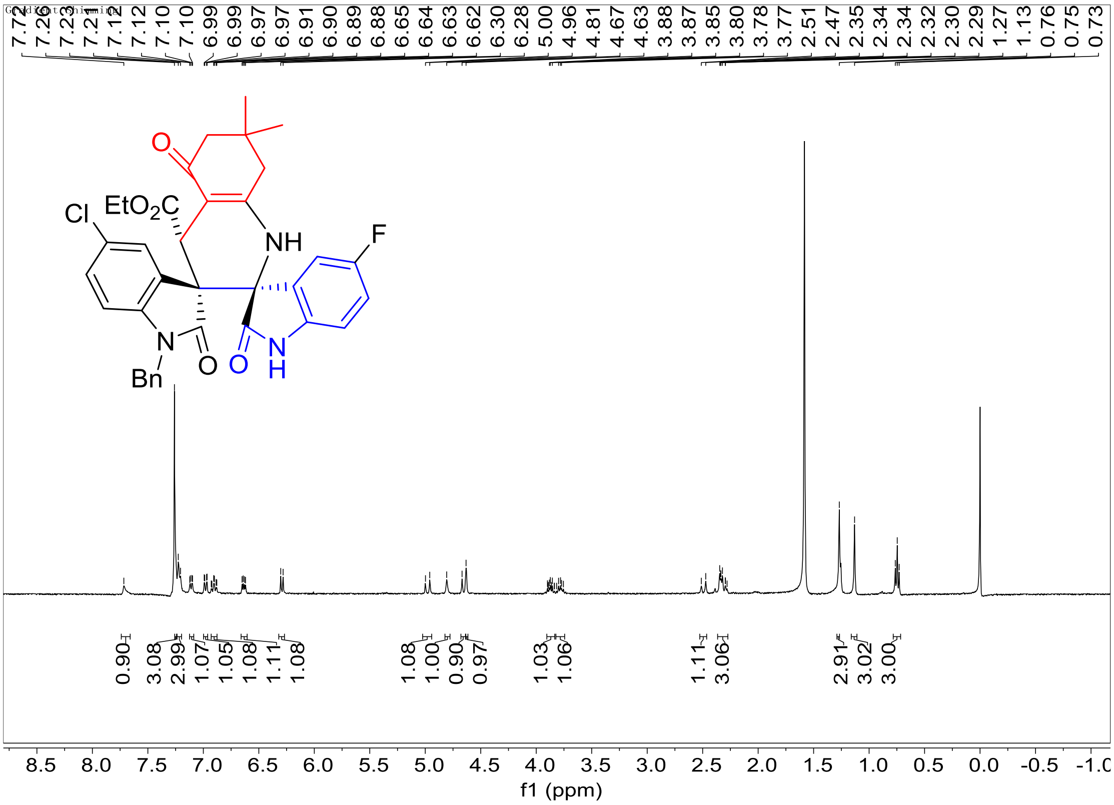

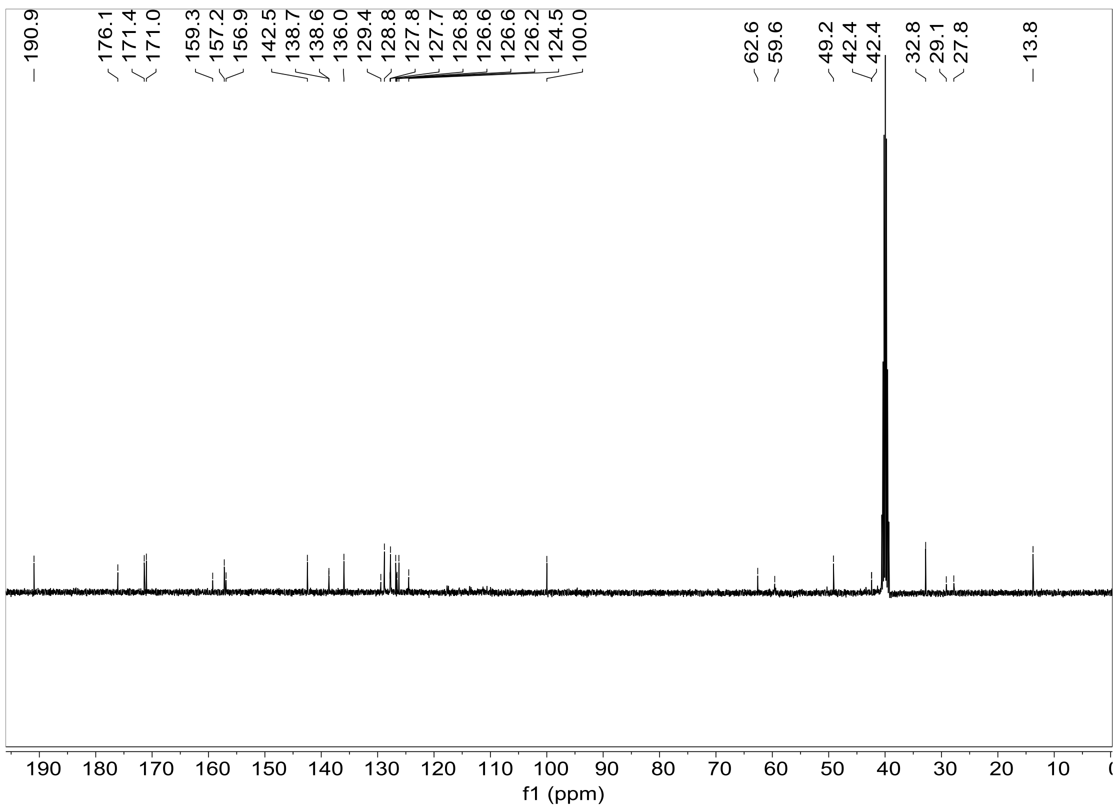
190.9
176.1
171.4
171.0
159.3
157.2
156.9
142.5
138.7
138.6
136.0
129.4
128.8
127.8
127.7
126.8
126.6
126.6
126.2
124.5
100.0
62.6
59.6
49.2
42.4
42.4
32.8
29.1
27.8
13.8
190 180 170 160 150 140 130 120 110 100 90 80 70 60 50 40 30 20 10
f1 (ppm)

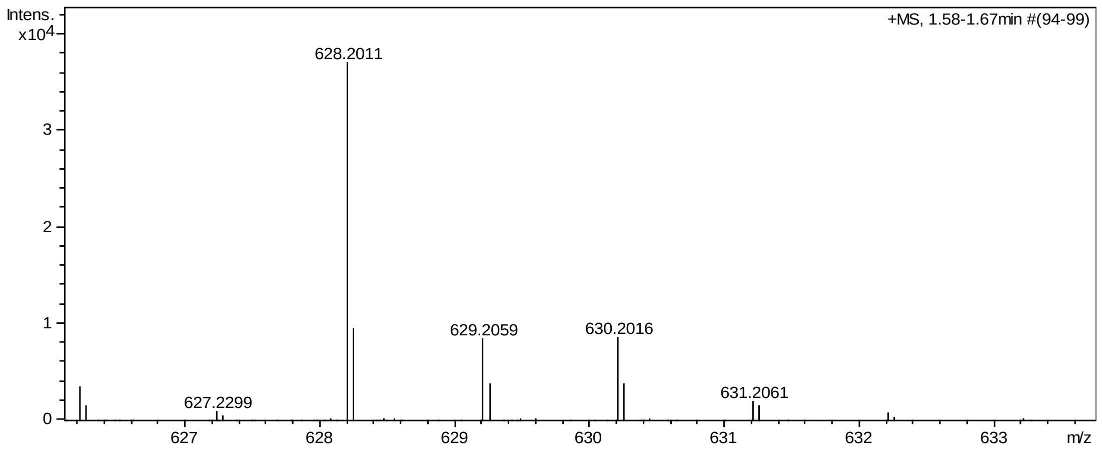
Intens.
x10^4
+MS, 1.58-1.67min #(94-99)
628.2011
629.2059
630.2016
631.2061
627.2299
627 628 629 630 631 632 633
m/z

Ethyl *rel*-(3*R*,3'*S*,4'*R*)-1-benzyl-5''-chloro-5,7',7'-trimethyl-2,2'',5'-trioxo-1',4',5',6',7',8'-hexahydrodispiro[indoline-3,2'-quinoline-3',3''-indoline]-4'-carboxylate (3i): White solid, 68%, m.p. >300ºC; ^{1}H NMR (400 MHz,CDCl$_3$) δ: 7.66 (s, 1H, NH), 7.33 - 7.30 (m, 4H, ArH), 7.20 - 7.18 (m, 3H, ArH), 7.07 - 7.04 (m, 1H, ArH), 6.89 (d, *J* = 8.4 Hz, 1H, ArH), 6.53 (d, *J* = 8.0 Hz, 1H, ArH), 6.36 (d, *J* = 8.0 Hz, 1H, ArH), 4.87 (d, *J* = 16.0 Hz, 1H, CH$_2$), 4.78 (d, *J* = 12.4, 1H, CH$_2$), 4.77 (s, 1H, NH), 4.69 (s, 1H, CH), 3.91 - 3.85 (m, 2H, CH$_2$),2.39 - 2.38 (s, 2H, CH$_2$), 2.33 (s, 2H, CH$_2$), 3.21 (s, 3H, CH$_3$), 1.28 (s, 3H, CH$_3$), 1.14 (s, 3H, CH$_3$), 0.87 (t, *J* = 7.2 Hz, 3H, CH$_3$) ppm; ^{13}C NMR (600 MHz, DMSO) δ: 190.3, 174.1, 172.6, 170.5, 156.8, 141.4, 140.2, 135.4, 131.7, 130.5, 128.7, 128.6, 127.4, 127.2, 125.9, 124.6, 124.1, 124.0, 110.3, 109.2, 99.5, 61.7, 58.8, 49.9, 48.9, 42.9, 42.0, 40.8, 32.3, 28.8, 27.2, 20.7, 13.3 ppm; IR (KBr) υ: 3610, 3526, 3026, 3002, 2985, 2953, 1823, 1756, 1625, 1521, 1465, 1228, 1214, 1023, 991, 863 cm^{-1}; HRMS (ESI-TOF) Calcd. for $C_{36}H_{35}ClN_3O_5$ ([M+H]$^+$): 624.2260, Found: 624.2256.

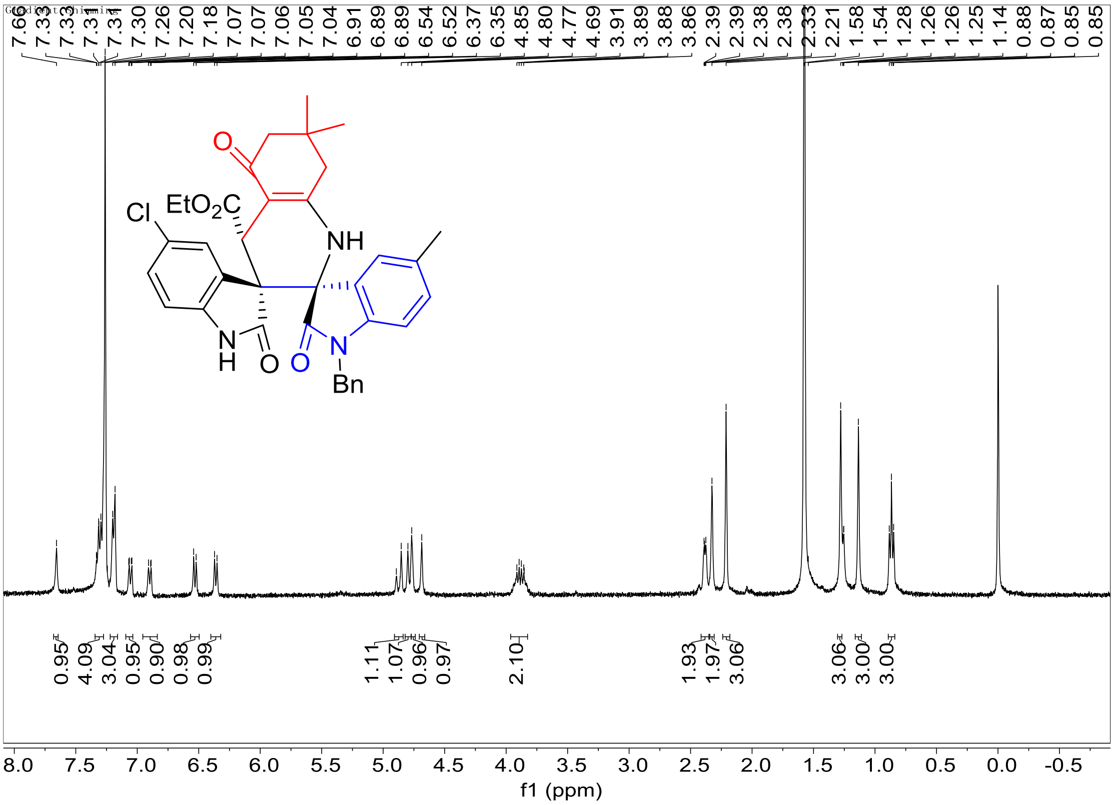

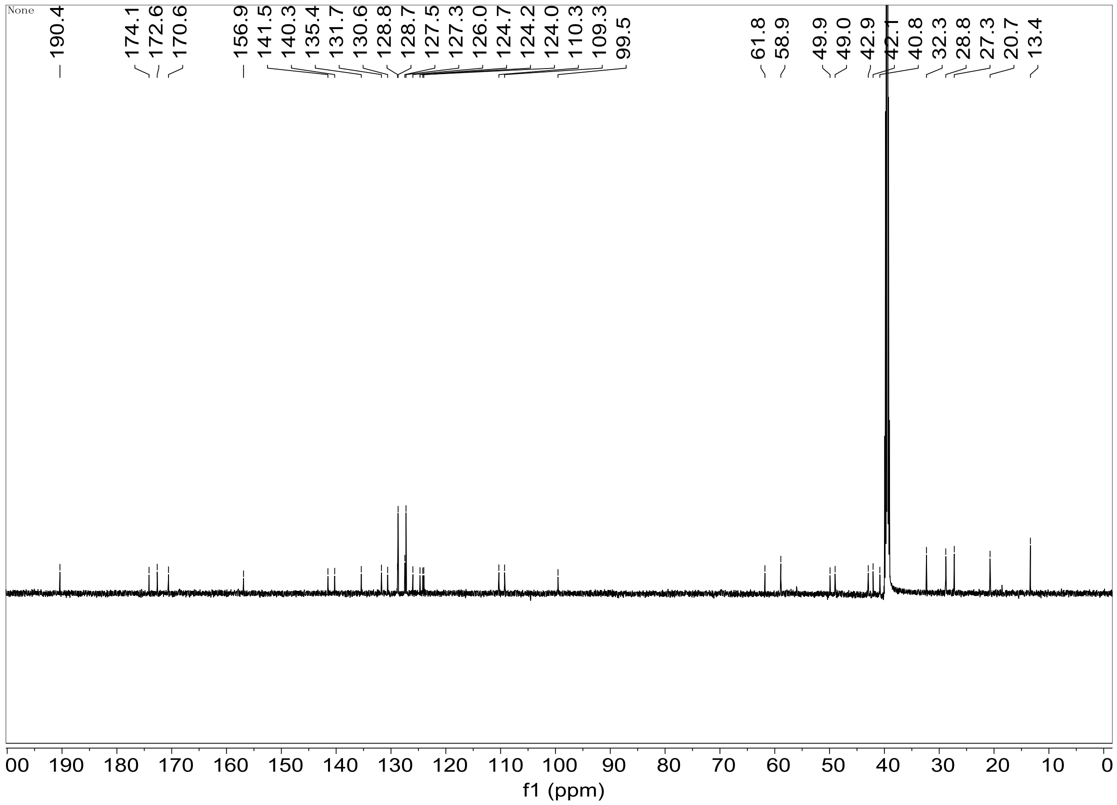
None
190.4
174.1
172.6
170.6
156.9
141.5
140.3
135.4
131.7
130.6
128.8
128.7
127.5
127.3
126.0
124.7
124.2
124.0
110.3
109.3
99.5
61.8
58.9
49.9
49.0
42.9
42.1
40.8
32.3
28.8
27.3
20.7
13.4
f1 (ppm)

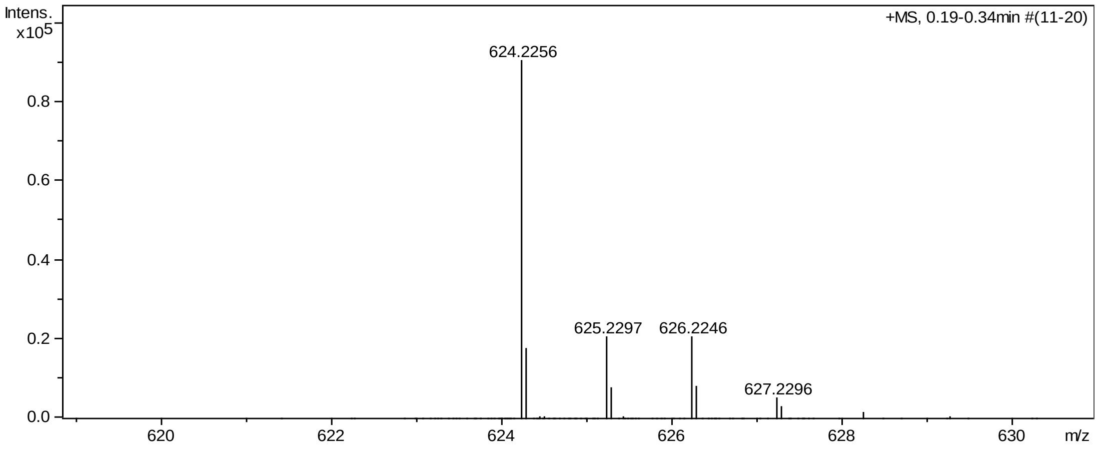
Intens.
x10^5
+MS, 0.19-0.34min #(11-20)
624.2256
625.2297
626.2246
627.2296
m/z

Methyl *rel*-(3*R*,3'*S*,4'*R*)-1,1''-dibenzyl-5''-chloro-5,7',7'-trimethyl-2,2'',5'-trioxo-1',4',5',6',7',8'-hexahydrodispiro[indoline-3,2'-quinoline-3',3''-indoline]-4'-carboxylate (3j): White solid, 72%, m.p. 270-272°C; ^{1}H NMR (400 MHz,CDCl$_3$) δ: 7.31 (q, J_1 = 6.8 Hz, J_2 = 14.0 Hz, 2H, ArH), 7.25 - 7.19 (m, 8H, ArH), 7.16 - 7.14 (m, 2H, ArH), 6.98 (d, *J* = 7.6 Hz, 1H, ArH), 6.92 (d, *J* = 8.8 Hz, 1H, ArH), 6.38 (d, *J* = 8.0 Hz, 1H, ArH), 6.24 (d, *J* = 8.4 Hz, 1H, ArH), 4.94 (d, *J* = 16.4 Hz,1H, CH$_2$), 4.89 (d, *J* = 16.4 Hz, 1H, ArH),4.82 (s, 1H, NH), 4.76 (d, *J* = 15.6 Hz, 1H, CH$_2$), 4.76 (s, 1H, CH), 4.70 (d, *J* = 16.0 Hz, 1H, CH$_2$), 3.37 (s, 3H, OCH$_3$), 2.41 (s, 2H, CH$_2$), 2.34 (s, 2H, CH$_2$), 2.14 (s, 3H, CH$_3$), 1.30 (s, 3H, CH$_3$), 1.15 (s, 3H, CH$_3$) ppm; ^{13}C NMR (400 MHz, CDCl$_3$) δ: 192.9, 174.1, 172.1, 172.0, 155.5, 141.9, 140.7, 135.0, 134.7, 132.9, 131.3, 129.1, 129.0, 128.6, 127.7, 127.5, 127.4, 127.2, 126.9, 126.5, 125.8, 124.8, 124.2, 109.6, 102.1, 62.4, 51.8, 51.8, 50.1, 50.0, 49.2, 44.3, 42.6, 42.6, 42.6, 42.5, 42.5, 32.8, 29.1, 27.6, 20.9, 20.9 ppm; IR (KBr) υ: 3513, 3031, 3001, 2995, 2986, 1875, 1721, 1612, 1521, 1429, 1242, 1214, 1029, 987, 853 cm^{-1}; HRMS (ESI-TOF) Calcd. for $C_{42}H_{39}ClN_3O_5$ ([M+H]$^+$): 700.2573, Found: 700.2567.

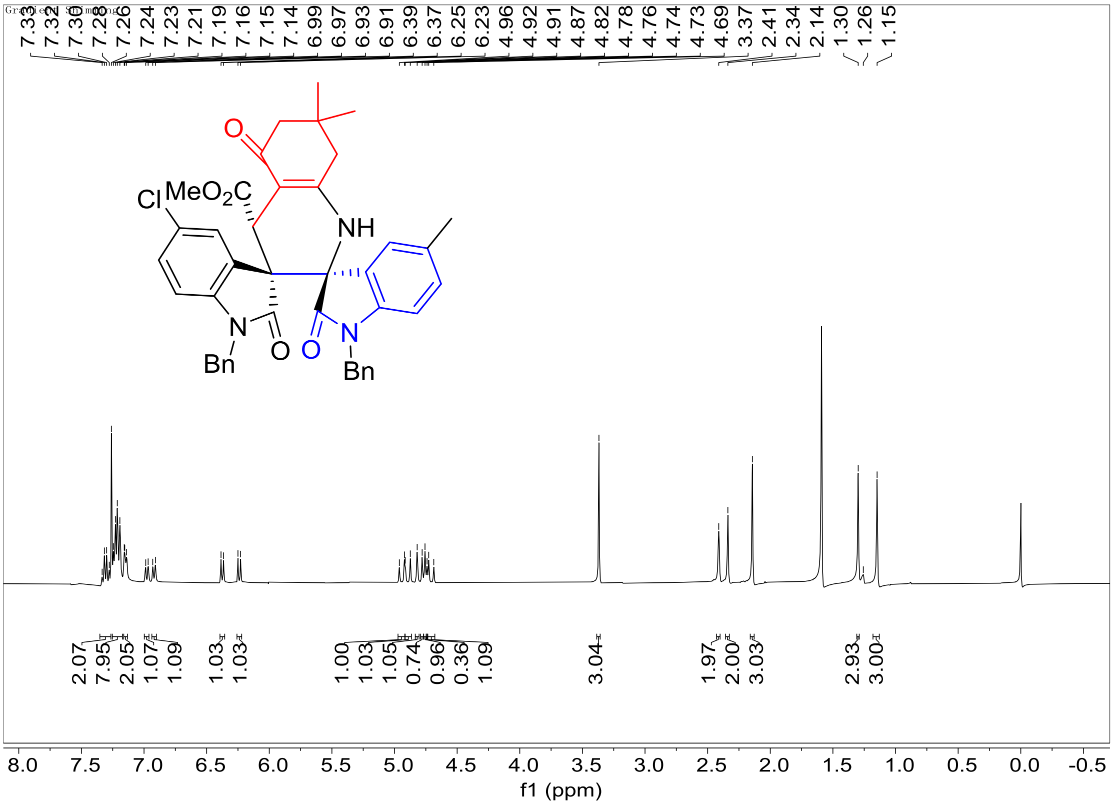

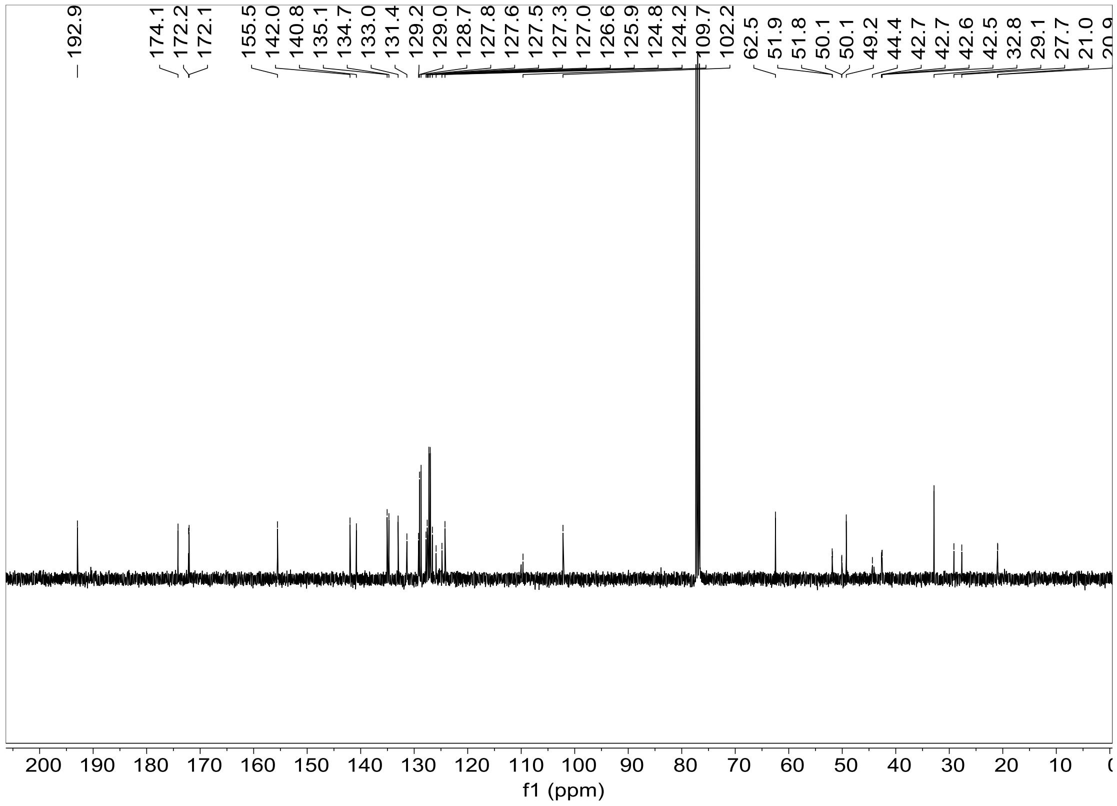
192.9
174.1
172.2
172.1
155.5
142.0
140.8
135.1
134.7
133.0
131.4
129.2
129.0
128.7
127.8
127.6
127.5
127.3
127.0
126.6
125.9
124.8
124.2
109.7
102.2
62.5
51.9
51.8
50.1
50.1
49.2
44.4
42.7
42.7
42.6
42.5
32.8
29.1
27.7
21.0
f1 (ppm)

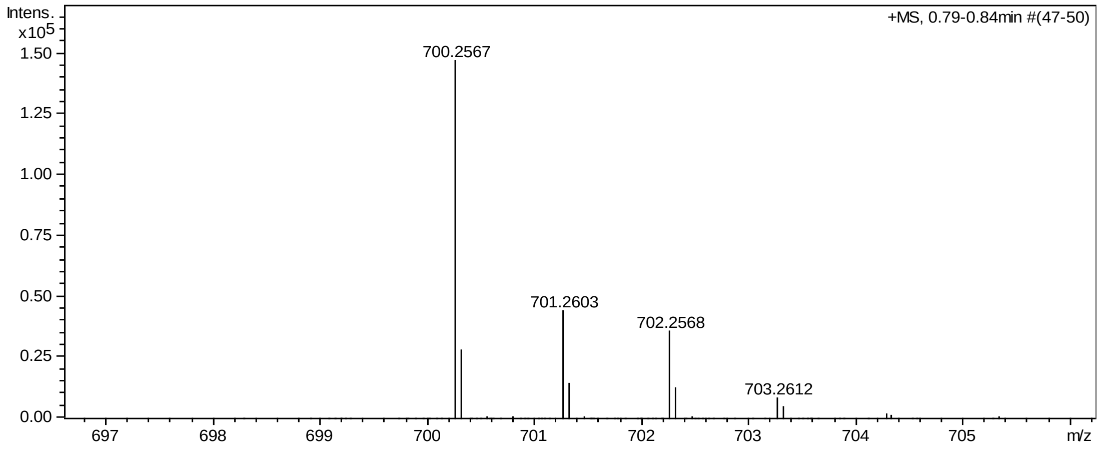
Intens.
x10^5
+MS, 0.79-0.84min #(47-50)
700.2567
701.2603
702.2568
703.2612
m/z

Methyl *rel*-(3*R*,3'*S*,4'*R*)-1,1''-dibenzyl-5''-fluoro-5,7',7'-trimethyl-2,2'',5'-trioxo-1',4',5',6',7',8'-hexahydrodispiro[indoline-3,2'-quinoline-3',3''-indoline]-4'-carboxylate (3k): White solid, 69%, m.p. 289-290°C; ^{1}H NMR (400 MHz,CDCl$_3$) δ: 7.33 - 7.29 (m, 3H, ArH), 7.25 - 7.20 (m, 6H, ArH), 7.17 - 7.15 (m, 2H, ArH), 6.95 - 6.91 (m, 2H, ArH), 6.72 - 6.67 (m, 1H, ArH), 6.43 (d, J = 8.0 Hz, 1H, ArH), 6.25 - 6.22 (m, 1H, ArH), 4.94 (d, J = 16.4 Hz, 1H, CH$_2$), 4.85 (d, J = 15.6, 1H, CH$_2$), 4.81 (s, 1H, NH), 4.79 (d, J = 16.0 Hz, 1H, CH$_2$), 4.76 (s, 1H, CH), 4.73 (d, J = 16.0 Hz, 1H, CH$_2$),3.35 (s, 3H, OCH$_3$), 2.46 - 2.40 (m, 2H, CH$_2$), 2.34 (s, 2H, CH$_2$), 2.16 (s, 3H, CH$_3$), 1.30 (s, 3H, CH$_3$), 1.15 (s, 3H, CH$_3$) ppm; ^{13}C NMR (400 MHz, CDCl$_3$) δ: 192.9, 174.1, 172.3, 172.0, 159.7, 157.3, 155.4, 140.8, 139.3, 135.2, 134.7, 133.0, 131.4, 128.9, 128.7, 127.9, 127.4, 127.4, 127.0, 126.0, 124.3, 115.4, 112.8, 112.5, 109.4, 102.3, 62.4, 51.8, 51.8, 50.1, 49.4, 49.3, 42.7, 42.6, 42.6, 32.9, 29.1, 27.7, 20.9 ppm; IR (KBr) υ: 3521, 3041, 3009, 2991, 2986, 1879, 1716, 1623, 1519, 1445, 1229, 1282, 1092, 994, 852 cm^{-1}; HRMS (ESI-TOF) Calcd. for $C_{42}H_{39}FN_3O_5$ ([M+H]$^+$): 684.2868, Found: 684.2864.

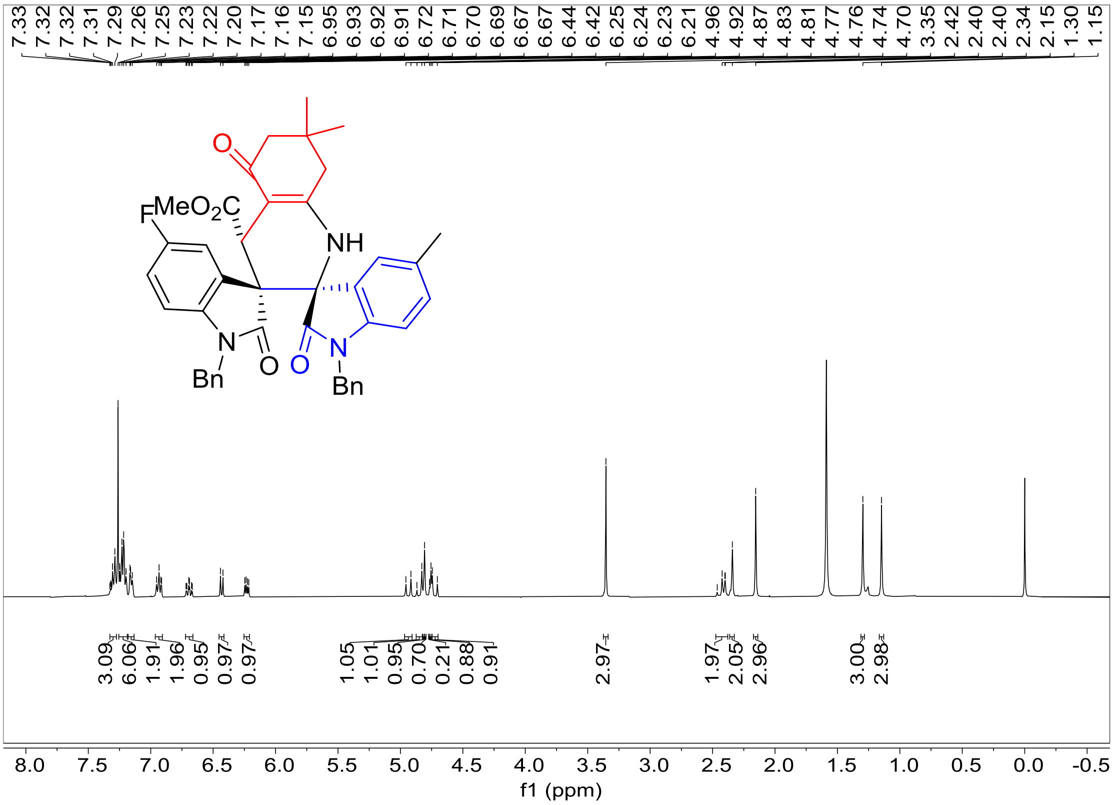

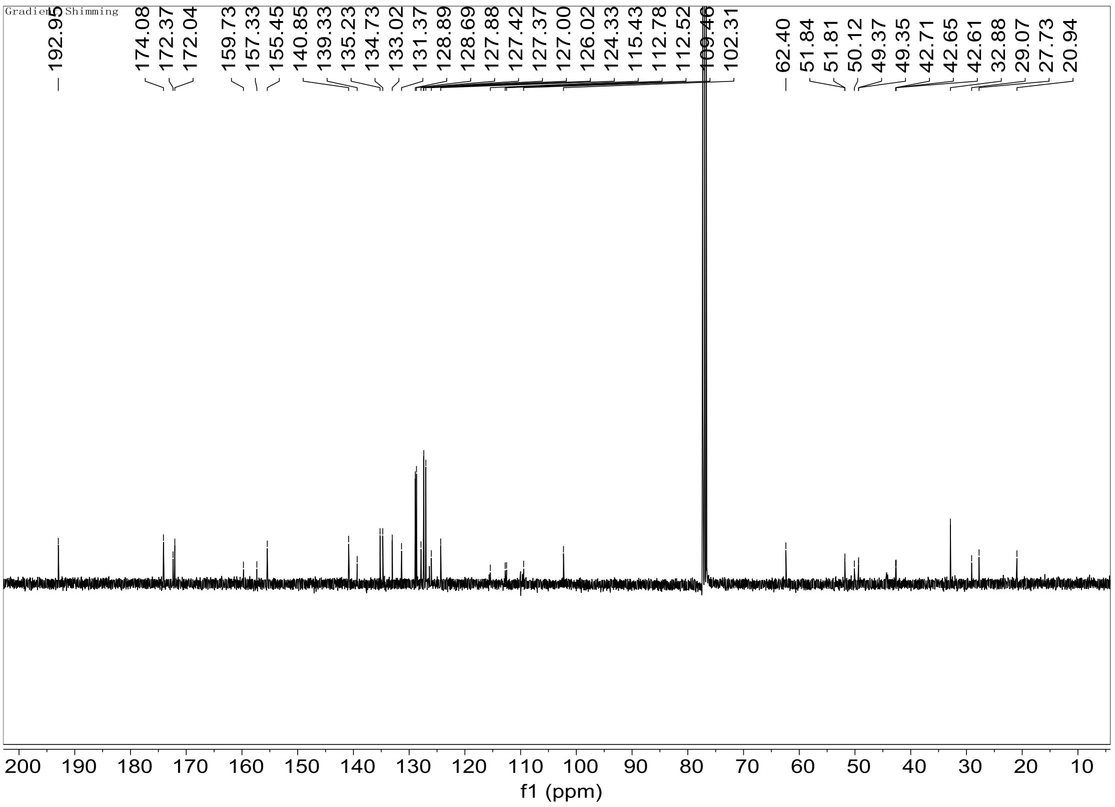
Gradient Shimming
192.95
174.08
172.37
172.04
159.73
157.33
155.45
140.85
139.33
135.23
134.73
133.02
131.37
128.89
128.69
127.88
127.42
127.37
127.00
126.02
124.33
115.43
112.78
112.52
109.46
102.31
62.40
51.84
51.81
50.12
49.37
49.35
42.71
42.65
42.61
32.88
29.07
27.73
20.94
200 190 180 170 160 150 140 130 120 110 100 90 80 70 60 50 40 30 20 10
f1 (ppm)

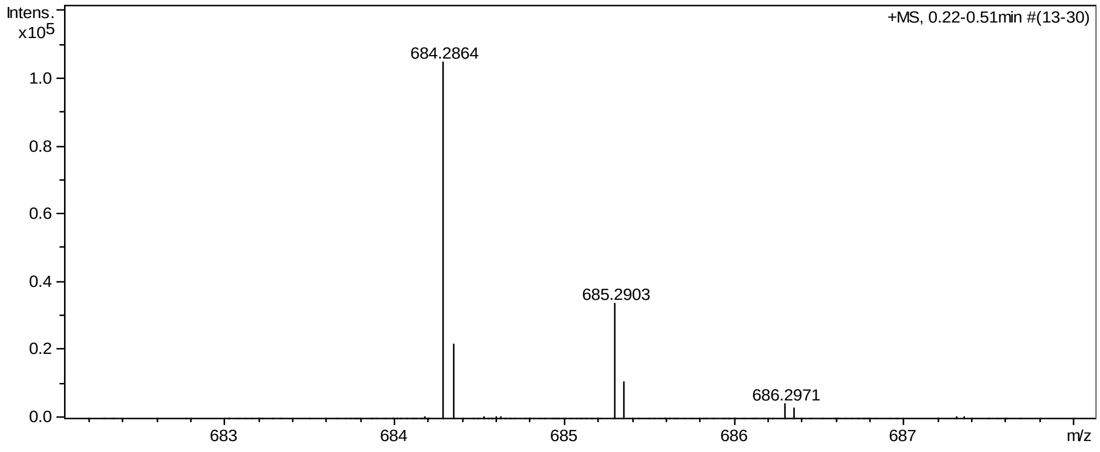
Intens.
x10^5
+MS, 0.22-0.51min #(13-30)
684.2864
685.2903
686.2971
1.0
0.8
0.6
0.4
0.2
0.0
683
684
685
686
687
m/z

Ethyl *rel*-(3*R*,3'*S*,4'*R*)-1,1''-dibenzyl-5''-chloro-7',7'-dimethyl-2,2'',5'-trioxo-1',4',5',6',7',8'-hexahydrodispiro[indoline-3,2'-quinoline-3',3''-indoline]-4'-carboxylate (3l): White solid, 42%, m.p. 275–277°C; ^{1}H NMR (400 MHz,$CDCl_3$) δ: 7.45 (d, *J* = 2.0 Hz, 1H, ArH), 7.31 - 7.28 (m, 4H, ArH), 7.24 - 7.10 (m, 7H, ArH), 7.03 (d, *J* = 8.0 Hz, 1H, ArH), 6.95 (t, *J* = 7.6 Hz, 1H, ArH), 6.69 (t, *J* = 7.6 Hz, 1H, ArH), 6.44 (d, *J* = 8.4 Hz, 1H, ArH), 6.31 (d, *J* = 8.0 Hz, 1H, ArH), 5.03 (d, *J* = 16.0 Hz, 1H, CH_2), 4.82 (d, *J* = 16.0 Hz, 2H, CH_2), 4.75 (d, *J* = 14.0 Hz, 2H, CH_2), 4.06 (d, *J* = 16.0 Hz, 1H, CH_2), 3.88 - 3.80 (m, 1H, CH_2), 3.78 -3.70 (m, 1H, CH_2), 2.49 (d, *J* = 16.0 Hz, 1H, CH_2), 2.40 - 2.30 (m, 3H, CH_2), 1.30 (s, 3H, CH_3), 1.15 (s, 3H, CH_3), 0.68 (t, *J* = 7.2 Hz, 3H, CH_3) ppm; ^{13}C NMR (400 MHz, $CDCl_3$) δ: 193.2, 174.1, 172.3, 171.4, 155.5, 143.3, 141.9, 135.3, 134.4, 130.9, 129.2, 128.9, 128.7, 128.5, 128.0, 127.7, 127.4, 127.2, 126.2, 125.7, 124.6, 124.1, 122.1, 110.3, 109.2, 102.8, 62.4, 60.3, 49.3, 42.8, 42.5, 32.9, 28.9, 27.8, 13.5 ppm; IR (KBr) υ: 3429, 1723, 1611, 1489, 1175, 953, 841 cm^{-1}; HRMS (ESI-TOF) Calcd. for $C_{42}H_{39}ClN_3O_5$ ($[M+H]^+$): 700.2573, Found: 700.2557.

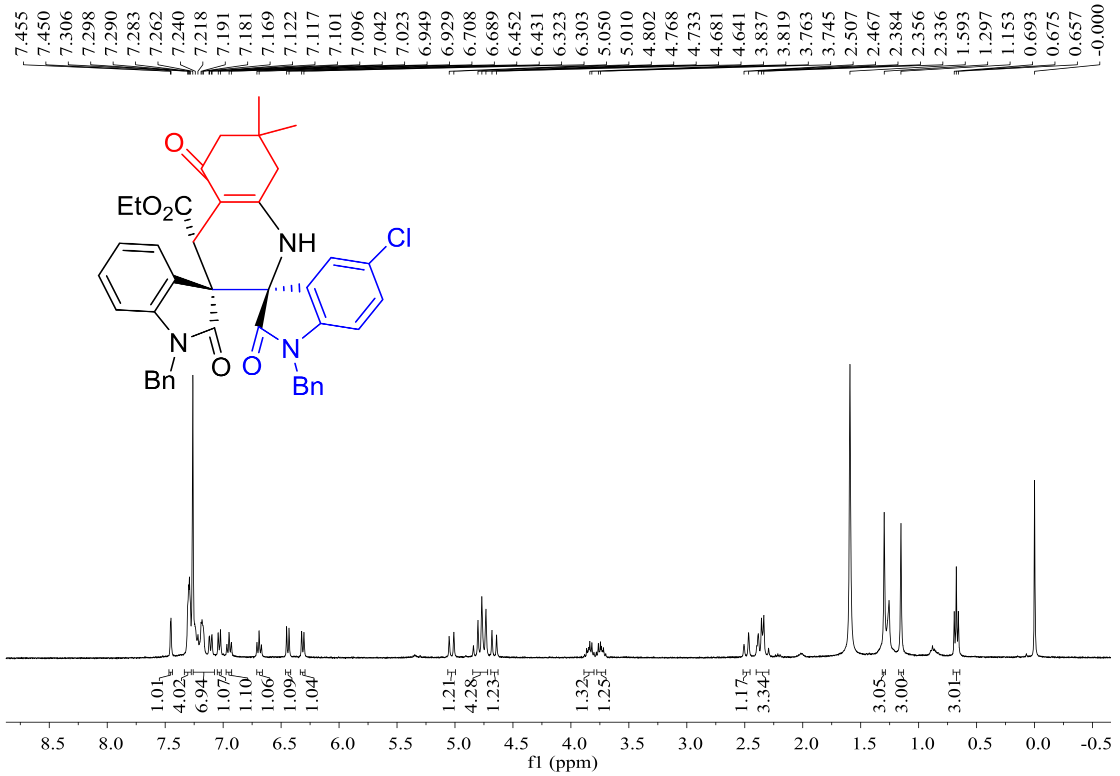

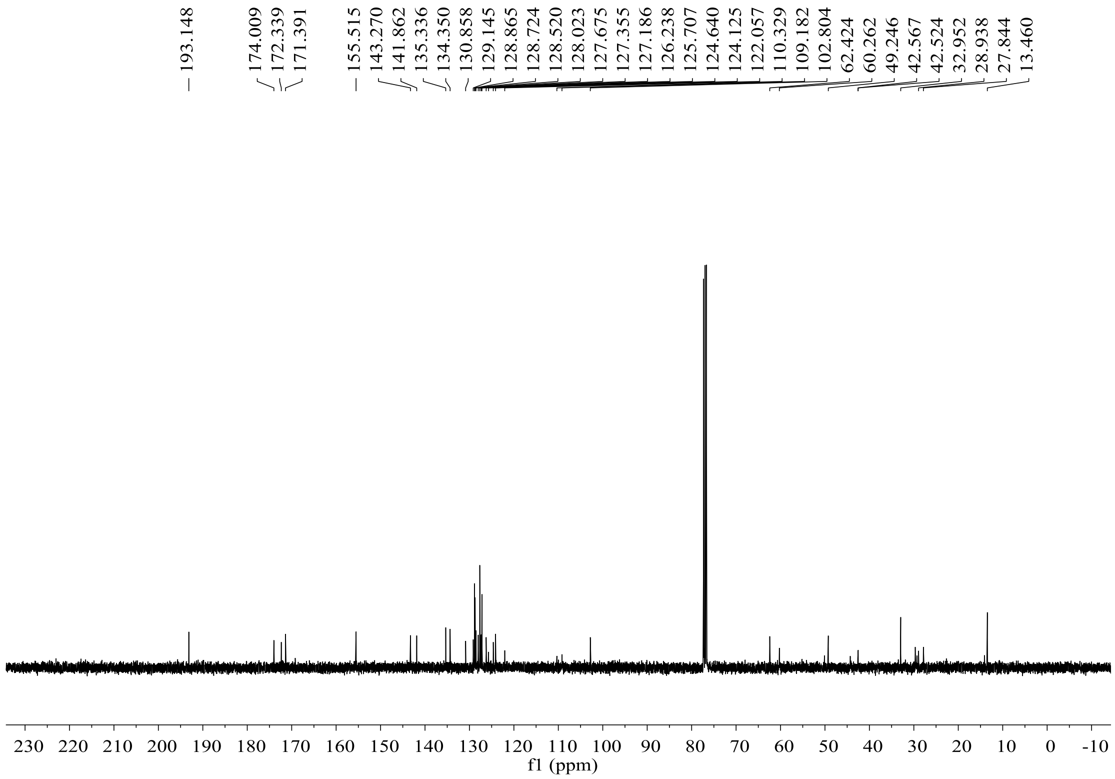
193.148
174.009
172.339
171.391
155.515
143.270
141.862
135.336
134.350
130.858
129.145
128.865
128.724
128.520
128.023
127.675
127.355
127.186
126.238
125.707
124.640
124.125
122.057
110.329
109.182
102.804
62.424
60.262
49.246
42.567
42.524
32.952
28.938
27.844
13.460
230 220 210 200 190 180 170 160 150 140 130 120 110 100 90 80 70 60 50 40 30 20 10 0 -10
f1 (ppm)

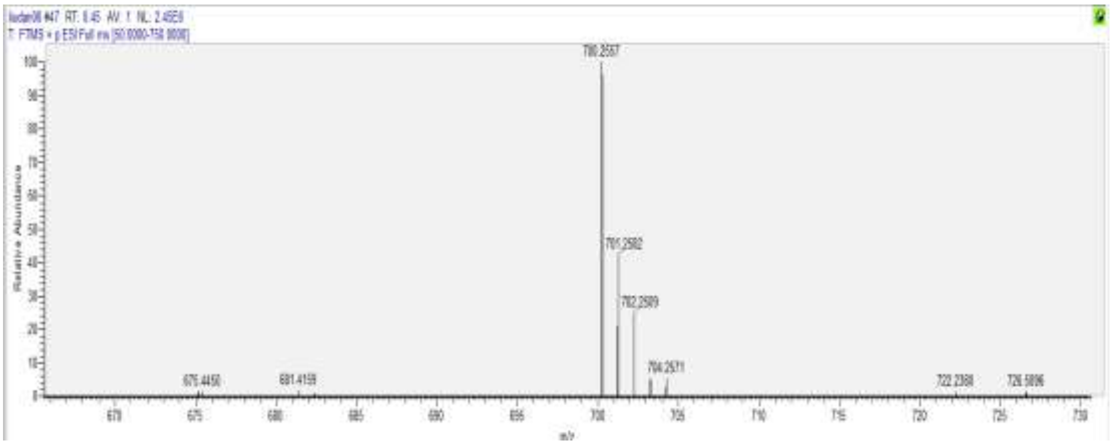
700.2557
701.2582
702.2589
704.2571
Relative Abundance

Ethyl *rel*-(3*R*,3'*S*,4'*R*)-1,1''-dibenzyl-5-chloro-5'',7',7'-trimethyl-2,2'',5'-trioxo-1',4',5',6',7',8'-hexahydrodispiro[indoline-3,2'-quinoline-3',3''-indoline]-4'-carboxylate (3m): White solid, 48%, m.p. 287–289°C; ^{1}H NMR (400 MHz,CDCl$_3$) δ: 7.45 (d, *J* = 2.0 Hz, 1H, ArH), 7.29 - 7.26 (m, 3H, ArH), 7.25 - 7.21 (m, 5H, ArH), 7.13 - 7.06 (m, 3H, ArH), 6.98 (s, 1H, ArH), 6.80 (dd, J_1= 8.0 Hz, J_2= 1.6 Hz, 1H, ArH), 6.37 (d, *J* = 8.4 Hz, 1H, ArH), 6.22 (d, *J* = 8.0 Hz, 1H, ArH),4.97 (d, *J* = 16.0 Hz, 1H, CH), 4.86 (d, *J* = 16.0 Hz, 1H, CH), 4.79 (d, *J* = 16.0 Hz, 1H, CH), 4.70 - 4.66 (m, 2H, CH$_2$), 3.88 - 3.80 (m, 1H, CH), 3.71 -3.66 (m, 1H, CH), 2.47 (d, *J* = 16.0 Hz, 1H, CH), 2.36 (d, *J* = 15.6 Hz, 1H, CH), 2.28 - 2.22 (m, 2H, CH$_2$), 2.12 (s, 3H, CH$_3$), 1.27 (s, 3H, CH$_3$), 1.12 (s, 3H, CH$_3$), 0.65 (t, *J* = 7.2 Hz, 3H, CH$_3$) ppm; ^{13}C NMR (400 MHz, CDCl$_3$) δ: 193.1, 174.2, 172.4, 171.4, 156.1, 156.0, 142.0, 141.0, 135.5, 134.4, 131.6, 130.8, 129.6, 129.0, 128.7, 128.6, 128.5, 127.8, 127.3, 127.2, 127.1, 126.4, 125.8, 125.4, 124.3, 110.7, 109.1, 102.5, 102.4, 62.5, 60.1, 50.0, 49.3, 42.7, 42.6, 42.4, 32.9, 29.0, 27.8, 21.1, 13.5 ppm; IR (KBr) υ: 3428, 1721, 1613, 1486, 1171, 951, 840 cm^{-1}; HRMS (ESI-TOF) Calcd. for $C_{43}H_{41}ClN_3O_5$ ([M+H]$^+$): 714.2729, Found: 714.2729.

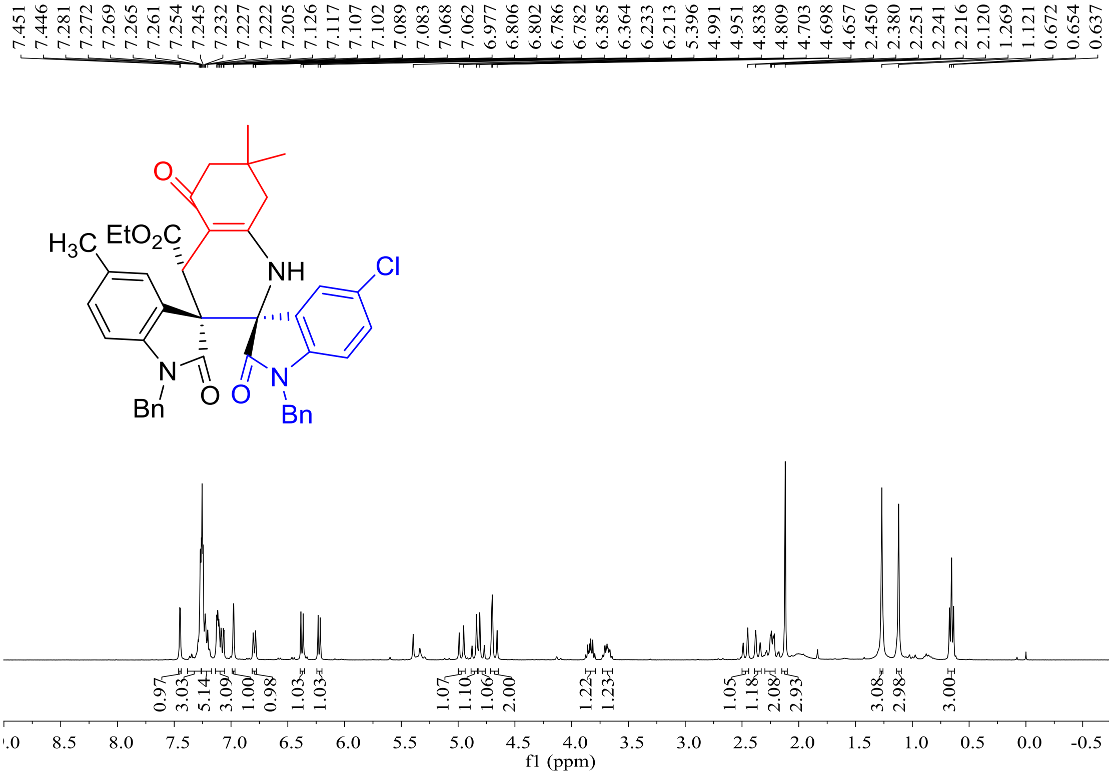

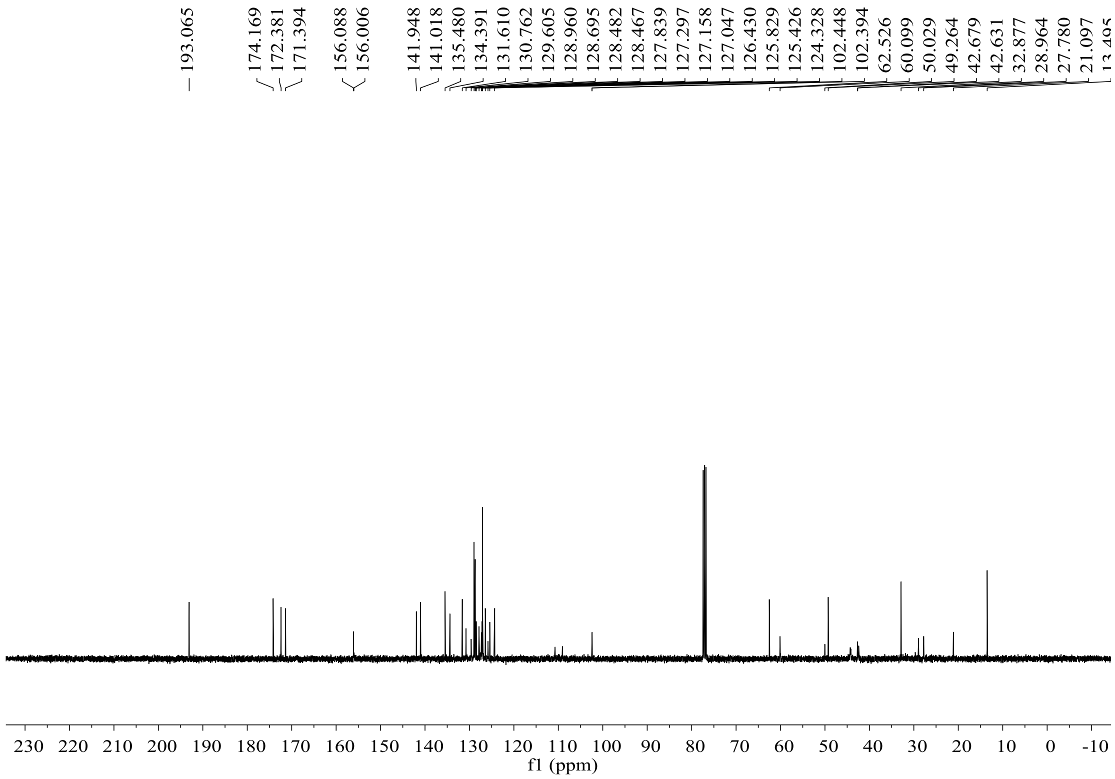
193.065
174.169
172.381
171.394
156.088
156.006
141.948
141.018
135.480
134.391
131.610
130.762
129.605
128.960
128.695
128.482
128.467
127.839
127.297
127.158
127.047
126.430
125.829
125.426
124.328
102.448
102.394
62.526
60.099
50.029
49.264
42.679
42.631
32.877
28.964
27.780
21.097
230 220 210 200 190 180 170 160 150 140 130 120 110 100 90 80 70 60 50 40 30 20 10 0 -10
f1 (ppm)

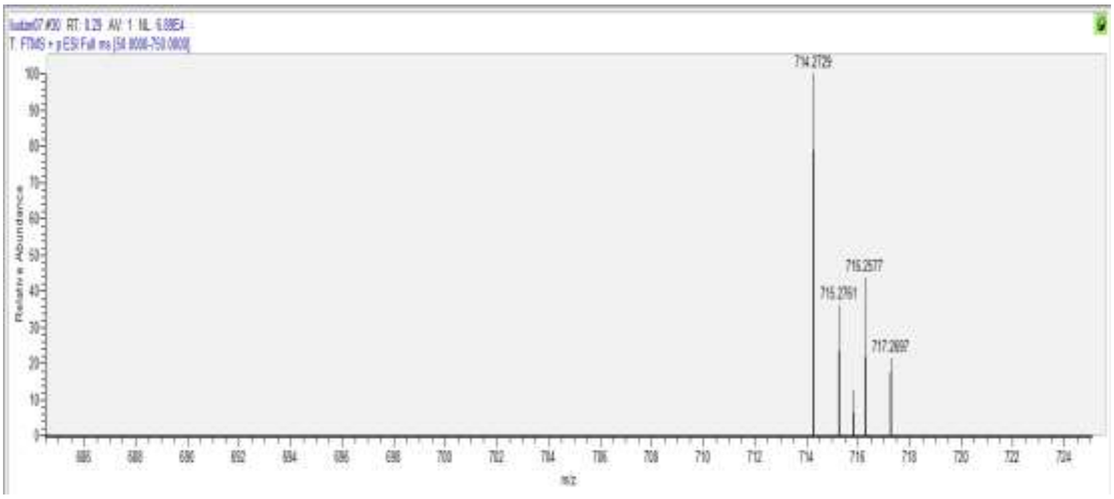
714.2729
715.2761
716.2577
717.2697
Relative Abundance

***rel*-(3*R*,3'*S*,4'*R*)-1,1''-Dibenzyl-5''-chloro-4'-(2-hydroxy-4,4-dimethyl-6-oxocyclohex-1-en-1-yl)-5-methyl-5'-(*p*-tolyl)-4'*H*-dispiro[indoline-3,2'-pyrrole-3',3''-indoline]-2,2''-dione (4a):**

White solid, 85%, m.p. 250-251°C; ^{1}H NMR (400 MHz,$CDCl_3$) δ: 10.86 (s, 1H, OH), 7.80 (d, *J* = 8.0 Hz, 2H, ArH), 7.51 (s, 1H, ArH), 7.21 (d, *J* = 8.4 Hz, 2H, ArH), 7.18 - 7.11 (m, 4H, ArH), 7.10 - 7.05 (m, 3H, ArH), 7.00 - 6.97 (m, 2H, ArH), 6.76 (d, *J* = 7.2 Hz, 2H, ArH), 6.65 (d, *J* = 7.6 Hz, 2H, ArH), 6.46 (d, *J* = 8.0 Hz, 1H, ArH), 6.28 (d, *J* = 8.4 Hz, 1H, ArH), 5.65 (s, 1H, CH), 5.18 (d, *J* = 16.4 Hz, 1H, CH_2), 5.09 (d, *J* = 16.0, 1H, CH_2), 4.47 (d, *J* = 4.8 Hz, 1H, CH_2), 4.43 (d, *J* = 5.2 Hz, 1H, CH_2), 2.41 (d, *J* = 26.8 Hz, 1H, CH_2), 2.40 (s, 3H, CH_3), 2.21 (d, *J* = 18.4 Hz, 1H, CH_2), 2.10 (s, 1H, CH_3), 2.05 (s, 1H, CH_2),1.87 (d, *J* = 16.0 Hz, 1H, CH_2), 1.00 (s, 3H, CH_3), 0.99 (s, 3H, CH_3) ppm; ^{13}C NMR (400 MHz, $CDCl_3$) δ: 197.5, 181.6, 177.4, 177.2, 173.0, 142.8, 142.2, 140.6, 134.7, 134.3, 133.9, 130.2, 130.0, 129.1, 128.7, 128.7, 128.5, 128.4, 127.5, 127.3, 127.1, 127.0, 126.4, 126.1, 126.1, 126.0, 112.1, 109.8, 109.7, 87.2, 61.8, 53.7, 50.0, 44.5, 44.4, 43.6, 30.7, 29.9, 26.3, 21.6, 20.9 ppm; IR (KBr) υ: 3756, 3056, 3023, 2984, 2988, 1832, 1792, 1526, 1545, 1368, 1285, 1145, 1025, 956, 882 cm^{-1}; HRMS (ESI-TOF) Calcd. for $C_{48}H_{43}ClN_3O_5$ ($[M+H]^+$): 760.2937, Found:760.2921.

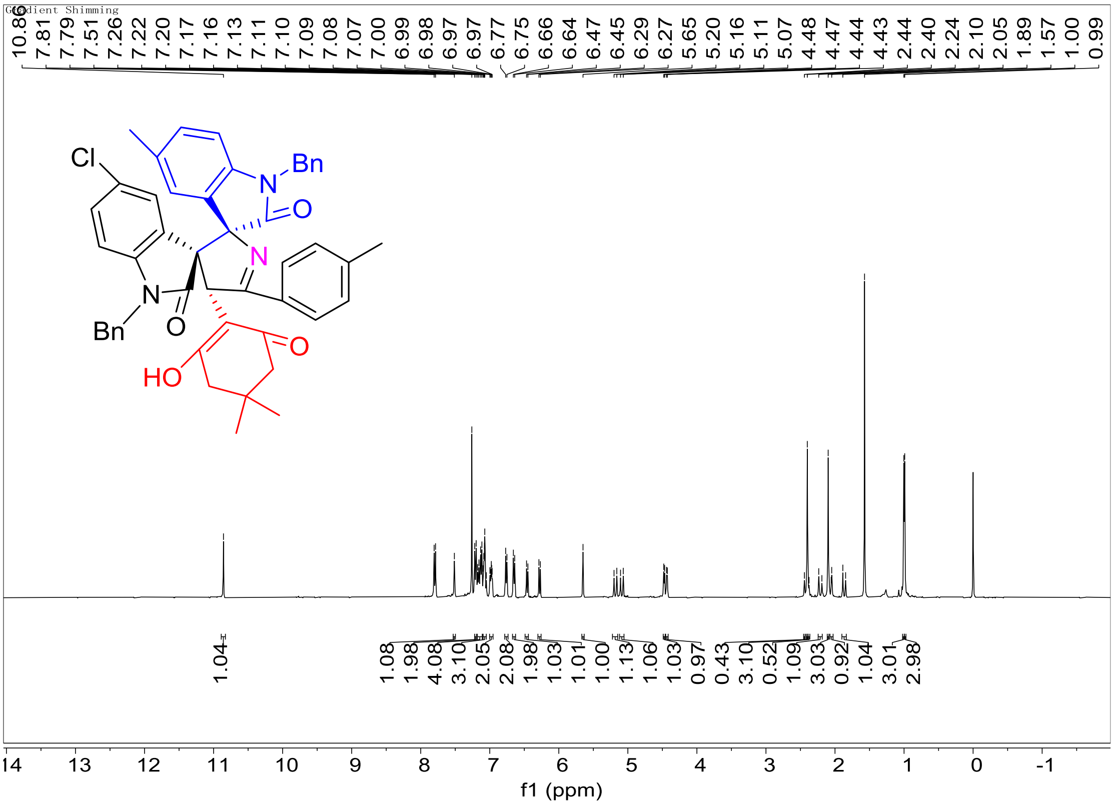

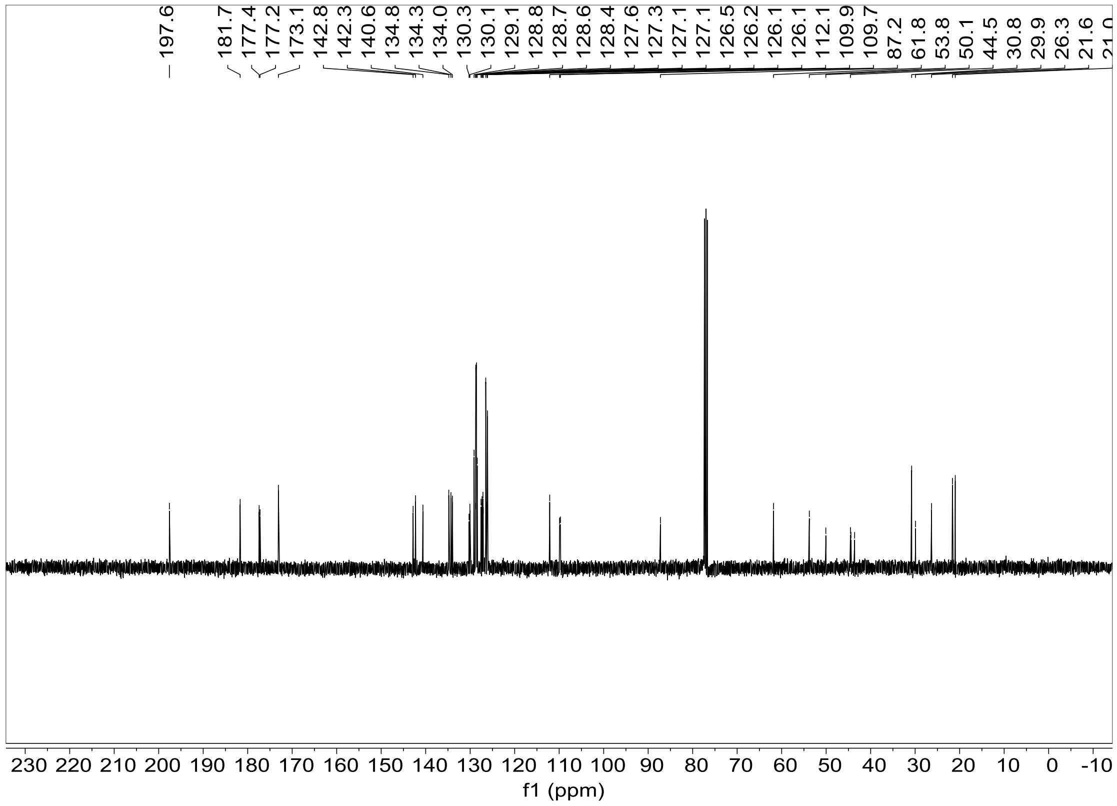
197.6
181.7
177.4
177.2
173.1
142.8
142.3
140.6
134.8
134.3
134.0
130.3
130.1
129.1
128.8
128.7
128.6
128.4
127.6
127.3
127.1
127.1
126.5
126.2
126.1
126.1
112.1
109.9
109.7
87.2
61.8
53.8
50.1
44.5
30.8
29.9
26.3
21.6
230 220 210 200 190 180 170 160 150 140 130 120 110 100 90 80 70 60 50 40 30 20 10 0 -10
f1 (ppm)

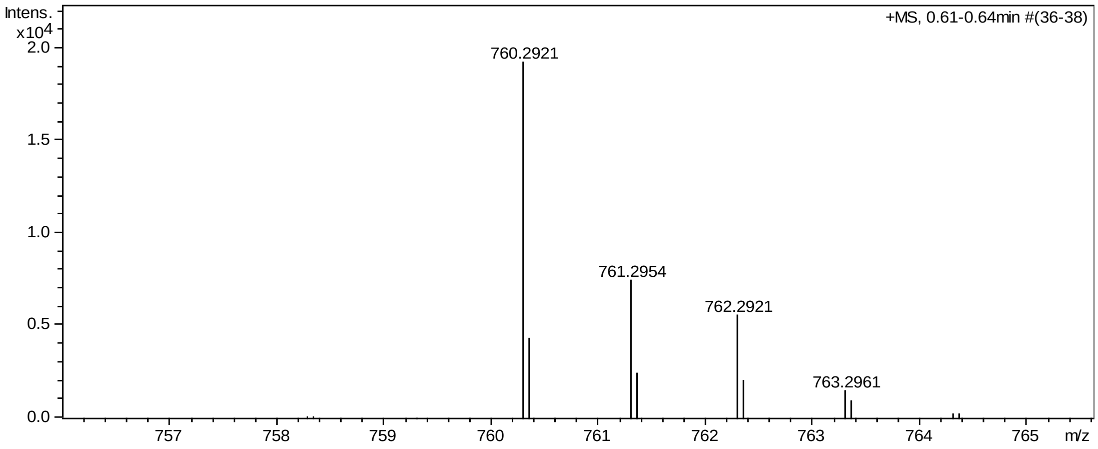
Intens.
x10^4
+MS, 0.61-0.64min #(36-38)
760.2921
761.2954
762.2921
763.2961
m/z

***rel*-(3*R*,3'*S*,4'*R*)-1,1''-Dibenzyl-5,5''-dichloro-4'-(2-hydroxy-4,4-dimethyl-6-oxocyclohex-1-en-1-yl)-5'-(*p*-tolyl)-4'*H*-dispiro[indoline-3,2'-pyrrole-3',3''-indoline]-2,2''-dione (4b):** White solid, 68%, m.p. 172-173°C; ^{1}H NMR (400 MHz,$CDCl_3$) δ: 10.58 (s, 1H, OH), 7.78 (d, *J* = 8.4Hz, 2H, ArH), 7.74 (d, *J* = 2.0 Hz, 1H, ArH), 7.21 (d, *J* = 7.6 Hz, 2H, ArH), 7.19 (d, *J* = 7.6 Hz, 1H, ArH), 7.16 - 7.12 (m, 6H, ArH), 7.06 (d, d, *J* = 2.0 Hz, 1H, ArH), 7.03 (d, *J* = 8.4 Hz, 1H, ArH), 6.83 (d, *J* = 6.0 Hz, 2H, ArH), 6.75 (d, *J* = 7.2Hz, 2H, ArH), 6.48 (d, *J* = 8.4 Hz, 1H, ArH), 6.36 (d, *J* = 8.4 Hz, 1H, CH), 5.65 (s, 1H, CH), 5.13 (d, *J* = 6.4, 1H, CH_2), 5.09 (d, *J* = 6.4 Hz, 1H, CH_2), 4.53 (d, *J* = 16.0 Hz, 1H, CH_2), 4.44 (d, *J* = 16.0 Hz, 1H, CH_2), 2.43 (d, *J* = 16.0 Hz, 1H, CH_2), 2.41 (s, 3H, CH_3), 2.22 (d, *J* = 18.8 Hz, 1H, CH_2), 2.08 (d, *J* = 15.6 Hz, 1H, CH_2),1.86 (d, *J* = 16.0 Hz, 1H, CH_2), 1.01 (s, 3H, CH_3), 0.99 (s, 3H, CH_3) ppm; ^{13}C NMR (400 MHz, $CDCl_3$) δ: 197.4, 182.2, 177.2, 176.9, 173.0, 142.8, 142.5, 141.5, 134.7, 133.4, 130.1, 129.9, 129.8, 129.2, 128.8, 128.8, 128.8, 128.7, 128.6, 128.5, 128.5, 128.4, 127.8, 127.4, 127.3, 126.5, 126.4, 126.1, 125.8, 112.0, 110.8, 86.8, 61.7, 54.0, 54.0, 50.0, 44.5, 44.5, 44.5, 30.8, 29.9, 26.3, 21.6, 21.6 ppm; IR (KBr) υ: 3763, 3027, 3017, 2956, 2865, 1829, 1756, 1701, 1565, 1365, 1329, 1152, 1029, 926, 806 cm^{-1}; HRMS (ESI-TOF) Calcd. for $C_{47}H_{40}Cl_2N_3O_4$ ($[M+H]^+$): 780.2390, Found: 780.2384.

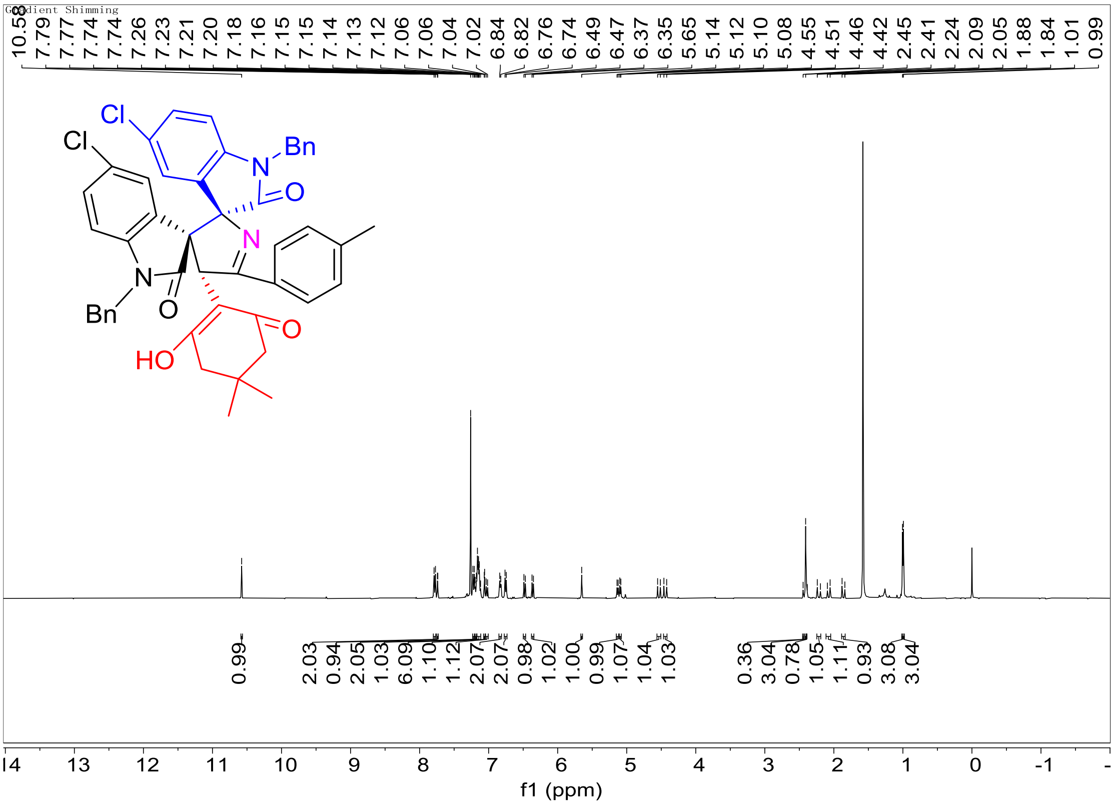

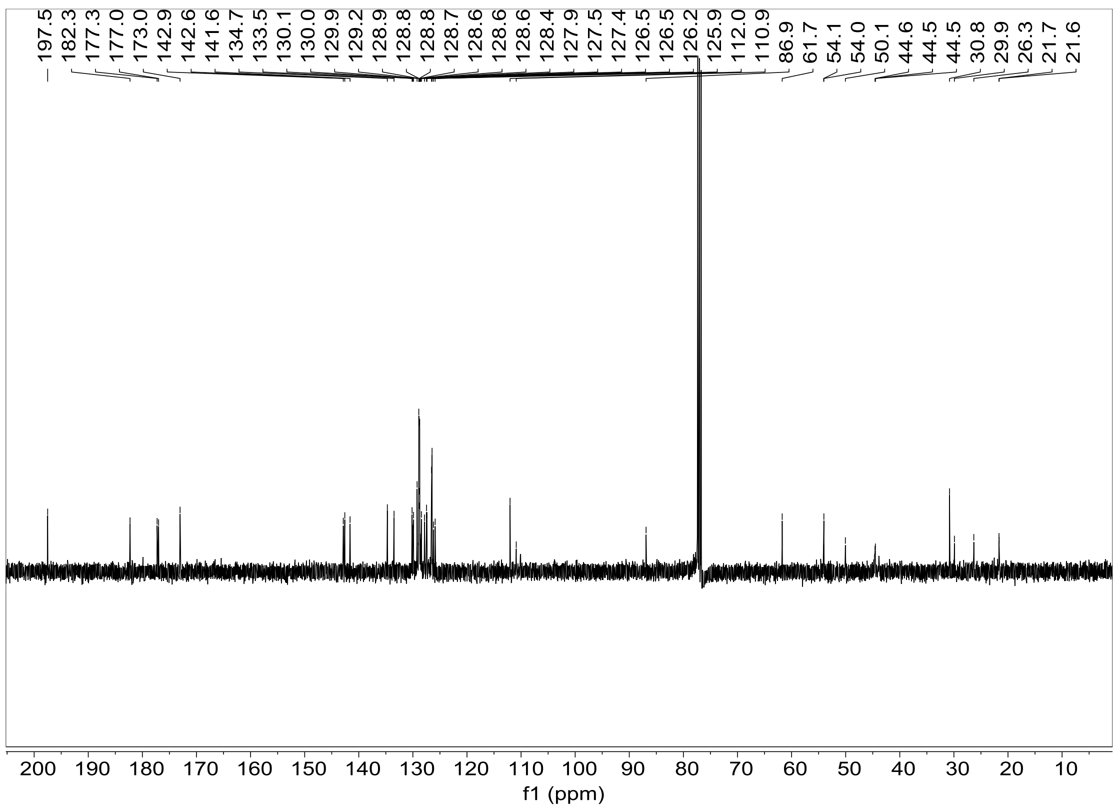
197.5
182.3
177.3
177.0
173.0
142.9
142.6
141.6
134.7
133.5
130.1
130.0
129.9
129.2
128.9
128.8
128.8
128.7
128.6
128.6
128.6
128.4
127.9
127.5
127.4
126.5
126.5
126.2
125.9
112.0
110.9
86.9
61.7
54.1
54.0
50.1
44.6
44.5
44.5
30.8
29.9
26.3
21.7
21.6
f1 (ppm)

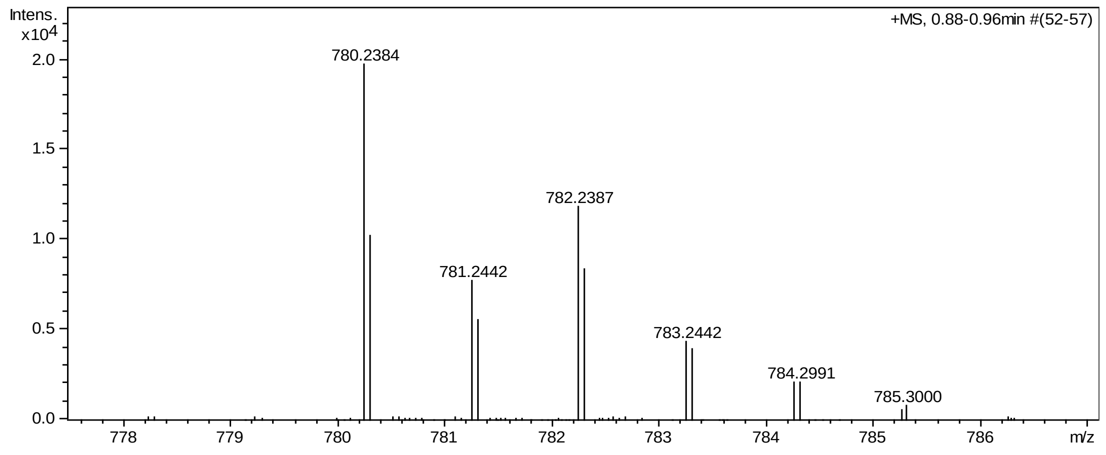
Intens.
x10^4
+MS, 0.88-0.96min #(52-57)
780.2384
782.2387
781.2442
783.2442
784.2991
785.3000
m/z

***rel*-(3*R*,3'*S*,4'*R*)-1,1''-Dibenzyl-5''-chloro-4'-(2-hydroxy-4,4-dimethyl-6-oxocyclohex-1-en-1-yl)-5'-(*p*-tolyl)-4'*H*-dispiro[indoline-3,2'-pyrrole-3',3''-indoline]-2,2''-dione (4c):** White solid, 75%, m.p. 251-252ºC; ^{1}H NMR (400 MHz,$CDCl_3$) δ: 10.75 (s, 1H, OH), 7.79 (d, *J* = 7.6 Hz, 2H, ArH), 7.69 (d, *J* = 6.8 Hz, 1H, ArH), 7.22 - 7.16 (m, 5H, ArH), 7.12 - 7.07 (m, 6H, ArH), 6.98 - 6.97 (m, 2H, ArH), 6.74 (d, *J* = 7.2 Hz, 2H, ArH), 6.67 (d, *J* = 6.8 Hz, 2H, ArH), 6.57 (d, *J* = 7.2 Hz, 1H, ArH), 6.26 (d, *J* = 8.4 Hz, 1H, ArH), 5.67 (s, 1H, CH), 5.14 (d, *J* = 16.0 Hz, 1H, CH_2), 5.08 (d, *J* = 16.4, 1H, CH_2), 4.48 (d, *J* = 17.2 Hz, 2H, CH_2), 2.44 (d, *J* = 5.2 Hz, 1H, CH_2), 2.40 (s, 3H, CH_3), 2.21 (d, *J* = 18.8 Hz, 1H, CH_2), 2.07 (d, *J* = 16.0 Hz, 1H, CH_2), 1.87 (d, *J* = 16.0 Hz, 1H, CH_2), 1.00 (s, 3H, CH_3), 0.99 (s, 3H, CH_3) ppm; ^{13}C NMR (400 MHz, $CDCl_3$) δ: 1 197.5, 181.7, 177.6, 177.1, 173.0, 143.1, 142.6, 142.3, 134.6, 133.8, 130.0, 130.0, 129.1, 128.7, 128.6, 128.4, 127.6, 127.3, 127.1, 127.1, 126.4, 126.3, 126.1, 112.1, 110.0, 87.2, 61.8, 53.7, 50.0, 44.4, 43.6, 30.8, 29.9, 26.3, 21.6 ppm; IR (KBr) υ: 3778, 3042, 3012, 2985, 2862, 1862, 1792, 1723, 1625, 1425, 1142, 1021, 956, 843 cm^{-1}; HRMS (ESI-TOF) Calcd. for $C_{47}H_{41}ClN_3O_4$($[M+H]^+$): 746.2780, Found: 746.2776.

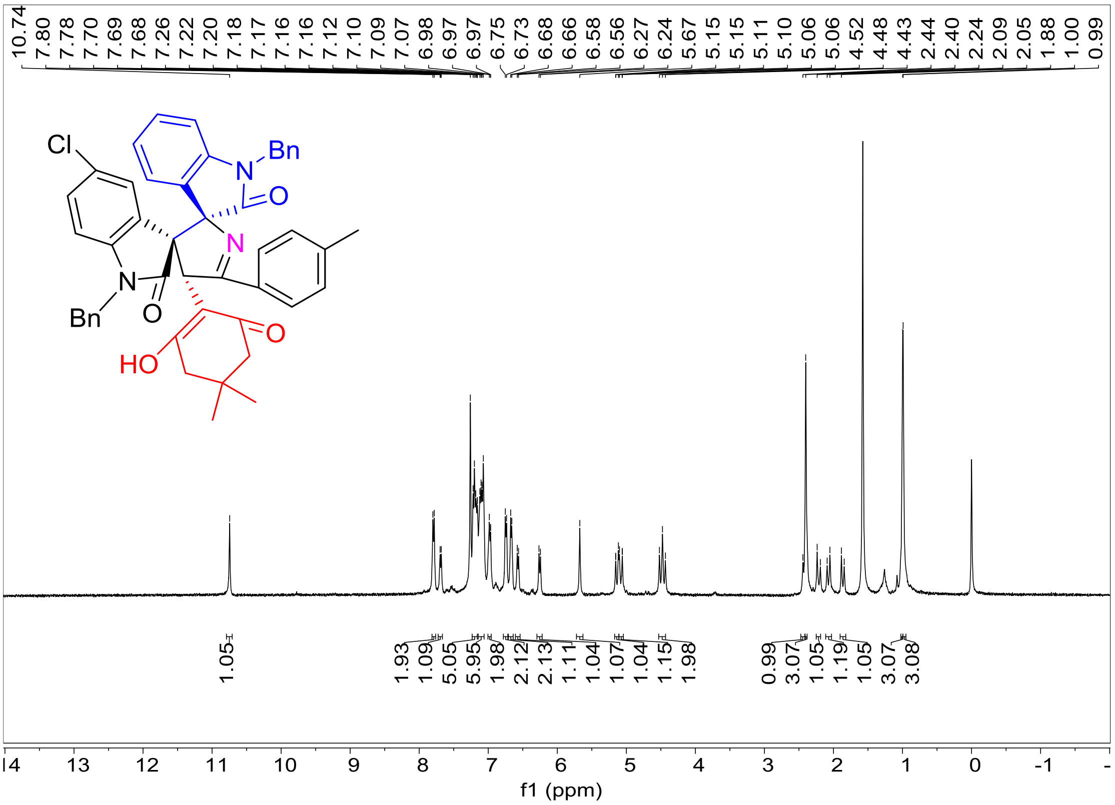

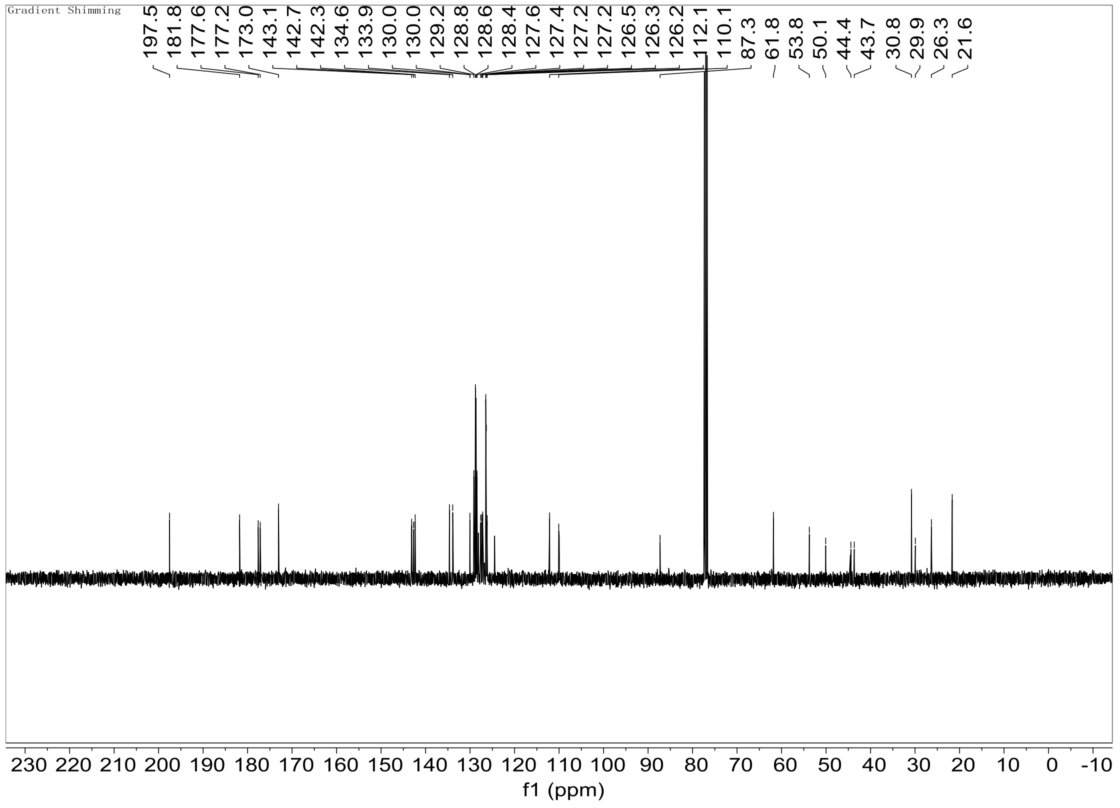
Gradient Shimming
197.5
181.8
177.6
177.2
173.0
143.1
142.7
142.3
134.6
133.9
130.0
130.0
129.2
128.8
128.6
128.4
127.6
127.4
127.2
127.2
126.5
126.3
126.2
112.1
110.1
87.3
61.8
53.8
50.1
44.4
43.7
30.8
29.9
26.3
21.6
230 220 210 200 190 180 170 160 150 140 130 120 110 100 90 80 70 60 50 40 30 20 10 0 -10
f1 (ppm)

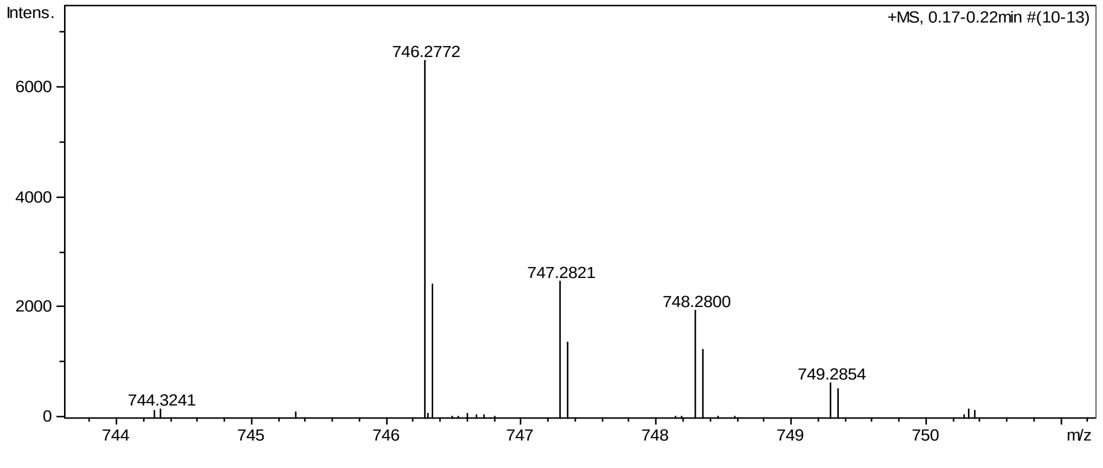
Intens.
+MS, 0.17-0.22min #(10-13)
746.2772
747.2821
748.2800
749.2854
744.3241
6000
4000
2000
0
744
745
746
747
748
749
750
m/z

***rel*-(3*R*,3'*S*,4'*R*)-1''-Benzyl-1-butyl-5''-chloro-4'-(2-hydroxy-4,4-dimethyl-6-oxocyclohex-1-en-1-yl)-5-methyl-5'-(*p*-tolyl)-4'*H*-dispiro[indoline-3,2'-pyrrole-3',3''-indoline]-2,2''-dione (4d):** White solid, 72%, m.p. 233-234°C; ^{1}H NMR (400 MHz,$CDCl_3$) δ: 11.04 (s, 1H, OH), 7.77 (d, *J* = 8.4 Hz, 2H, ArH), 7.50 (s, 1H, ArH), 7.19 (d, *J* = 7.6 Hz, 2H, ArH), 7.17 (d, *J* = 7.2 Hz, 1H, ArH), 7.14 - 7.09 (m, 3H, ArH), 7.07 (d, *J* = 2.0 Hz, 1H, ArH), 6.96 - 6.93 (d, *J* = 2.0 Hz, 1H, ArH), 6.68 - 6.64 (m, 3H, ArH), 6.23 (d, *J* = 8.4 Hz, 1H, ArH), 5.59 (s, 1H, CH), 5.18 (d, *J* = 16.8 Hz, 1H, CH_2), 4.42 (d, *J* = 16.0, 1H, CH_2), 3.79 - 3.72 (m, 1H, CH_2), 3.33 - 3.25 (m, 1H, CH_2),4.43 (d, *J* = 17.6 Hz, 1H, CH_2), 2.39 (s, 3H, CH_3), 2.22 (d, *J* = 18.8 Hz, 1H, CH_2), 2.14 (s, 1H, CH_3), 2.06 (d, *J* = 16.0 Hz, 1H, CH_2), 1.86 (d, *J* = 15.6 Hz, 1H, CH_2), 1.39 - 1.33 (m, 2H, CH_2), 1.16 - 1.07 (m, 2H, CH_2), 1.01 (s, 3H, CH_3), 0.99 (s, 3H, CH_3) ppm; ^{13}C NMR (400 MHz, $CDCl_3$) δ: 234.3, 197.5, 181.5, 177.2, 177.0, 173.1, 142.7, 142.1, 140.9, 134.9, 134.0, 130.1, 129.0, 128.8, 128.5, 128.4, 128.3, 127.2, 127.1, 126.9, 126.1, 126.0, 125.9, 112.1, 109.7, 109.7, 108.6, 86.8, 61.8, 53.7, 50.1, 44.6, 43.6, 43.6, 40.6, 30.8, 29.9, 29.4, 26.3, 21.6, 20.9, 20.1, 13.6 ppm; IR (KBr) υ: 3785, 3062, 3002, 2981, 2863, 1872, 1786, 1712, 1625, 1429, 1129, 1052, 956, 821 cm^{-1}; HRMS (ESI-TOF) Calcd. for $C_{45}H_{45}ClN_3O_4$($[M+H]^+$): 726.3093, Found: 726.3082.

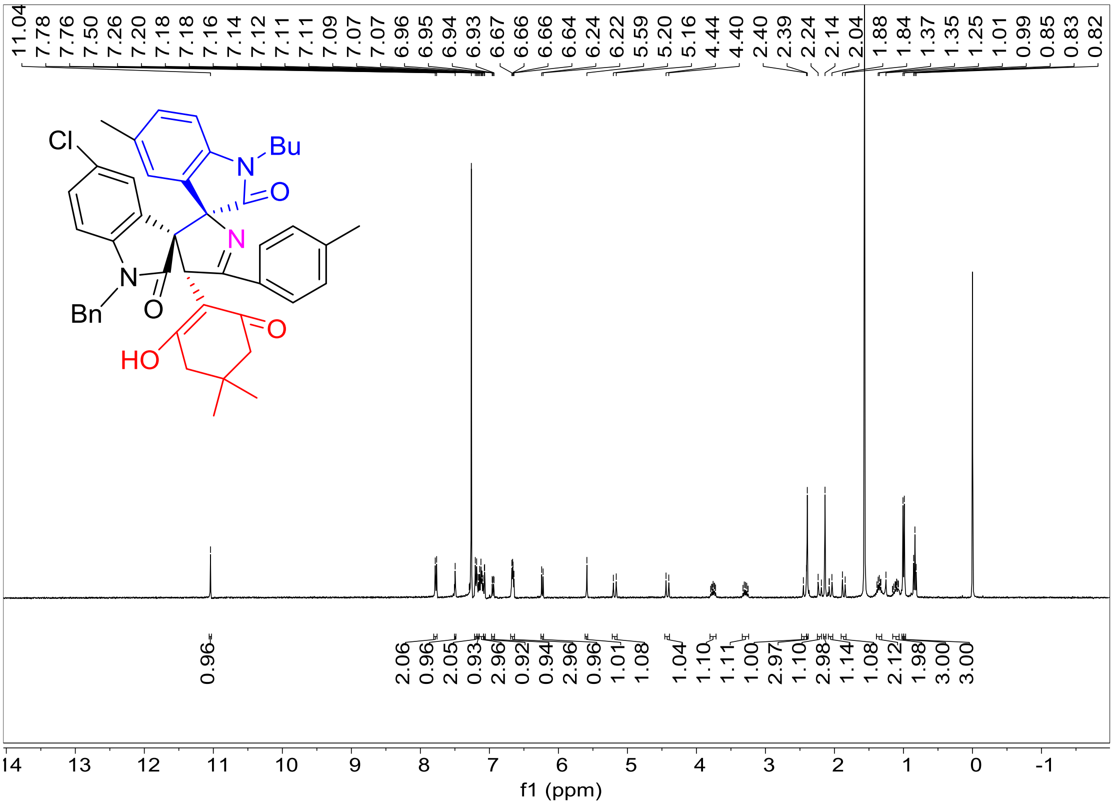

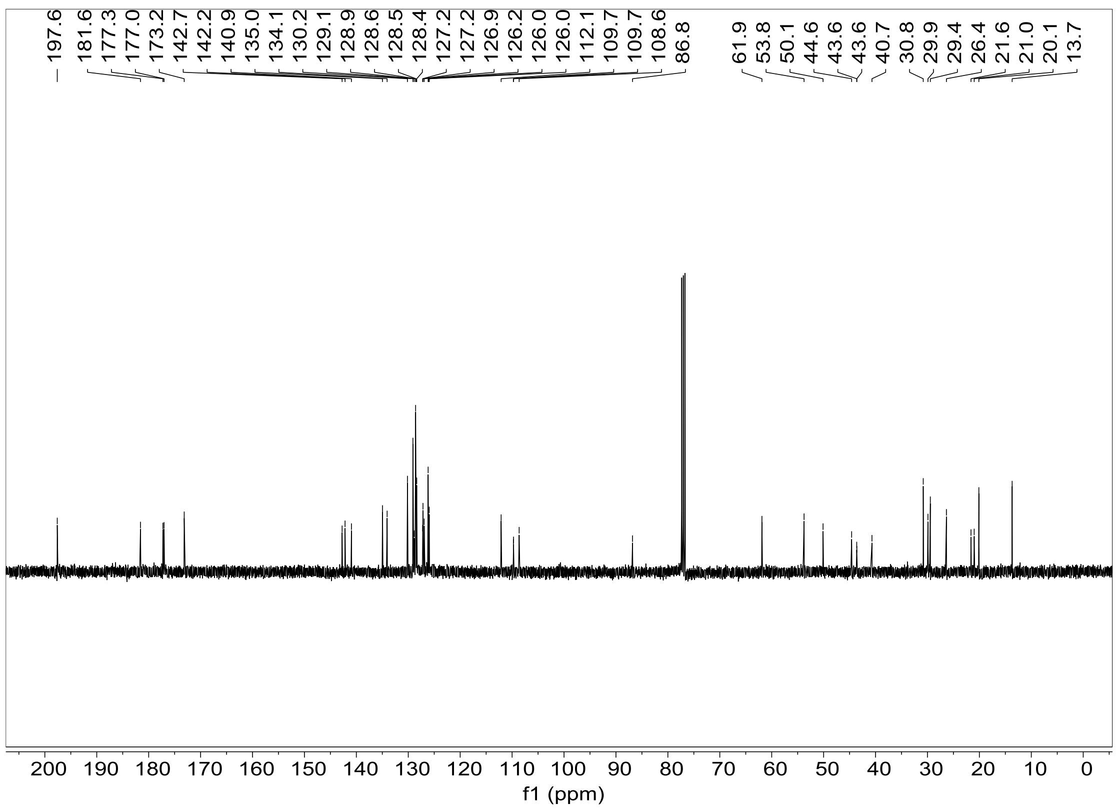
197.6
181.6
177.3
177.0
173.2
142.7
142.2
140.9
135.0
134.1
130.2
129.1
128.9
128.6
128.5
128.4
127.2
127.2
126.9
126.2
126.0
126.0
112.1
109.7
109.7
108.6
86.8
61.9
53.8
50.1
44.6
43.6
43.6
40.7
30.8
29.9
29.4
26.4
21.6
21.0
20.1
13.7
200 190 180 170 160 150 140 130 120 110 100 90 80 70 60 50 40 30 20 10 0
f1 (ppm)

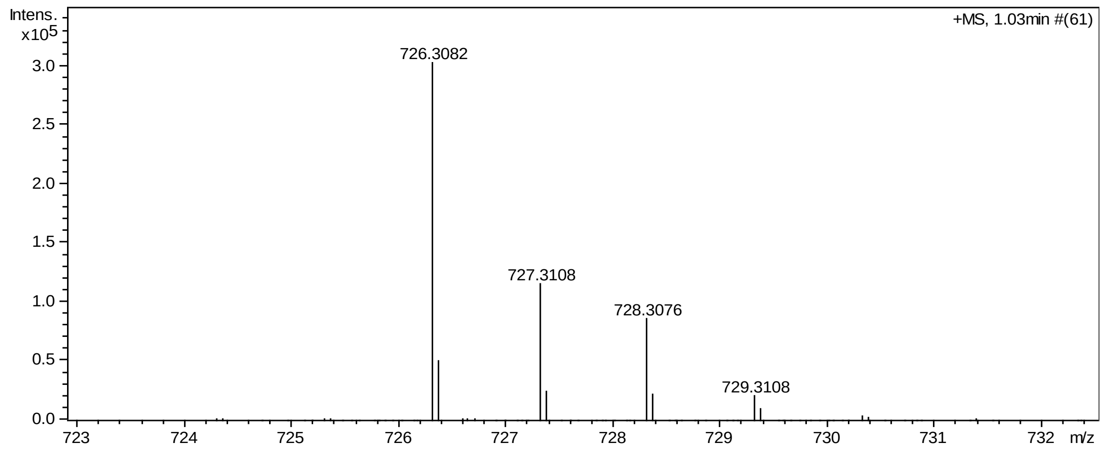
Intens.
x10^5
+MS, 1.03min #(61)
726.3082
727.3108
728.3076
729.3108
3.0
2.5
2.0
1.5
1.0
0.5
0.0
723 724 725 726 727 728 729 730 731 732 m/z

***rel*-(3*R*,3'*S*,4'*R*)-1,1''-Dibenzyl-5''-chloro-5'-(4-chlorophenyl)-4'-(2-hydroxy-4,4-dimethyl-6-oxocyclohex-1-en-1-yl)-5-methyl-4'*H*-dispiro[indoline-3,2'-pyrrole-3',3''-indoline]-2,2''-dione (4e):** White solid, 63%, m.p. 264-265°C; ^{1}H NMR (400 MHz, $CDCl_3$) δ: 10.90 (s, 1H, OH), 7.84 (d, *J* = 8.0 Hz, 2H, ArH), 7.50 (s, 1H, ArH), 7.39 (d, *J* = 8.4 Hz, 2H, ArH), 7.20 - 7.16 (m, 2H, ArH), 7.13 (d, *J* = 6.4 Hz, 2H, ArH), 7.11 - 7.09 (m, 1H, ArH), 7.00 - 6.99 (m, 2H, ArH), 6.77 (d, *J* = 7.2 Hz, 2H, ArH), 6.66 (d, *J* = 7.2 Hz, 2H, ArH), 6.48 (d, *J* = 7.6 Hz, 1H, ArH), 6.29 (d, *J* = 8.8 Hz, 1H, ArH), 5.63 (s, 1H, CH), 5.16 (d, *J* = 16.4 Hz, 1H, CH_2), 5.08 (d, *J* = 16.4, 1H, CH_2), 4.49 (d, *J* = 6.8 Hz, 1H, CH_2), 4.49 (d, *J* = 6.8 Hz, 1H, CH_2), 4.45 (d, *J* = 7.2 Hz, 1H, CH_2), 2.42 (d, *J* = 18.4 Hz, 1H, CH_2), 2.22 (d, *J* = 18.0 Hz, 1H, CH_2), 2.11 (s, 3H, CH_3), 2.07 (d, *J* = 16.4 Hz, 1H, CH_2),1.87 (d, *J* = 16.4 Hz, 1H, CH_2), 1.01 (s, 3H, CH_3), 0.98 (s, 3H, CH_3) ppm; ^{13}C NMR (400 MHz, $CDCl_3$) δ: 197.5, 180.8, 177.1, 173.6, 173.3, 142.8, 140.6, 138.0, 134.7, 134.5, 133.9, 130.4, 129.7, 129.1, 129.0, 129.0, 128.8, 128.8, 128.7, 128.6, 128.5, 127.6, 127.4, 127.2, 126.9, 126.8, 126.5, 126.4, 126.1, 125.9, 111.8, 109.9, 61.8, 53.8, 50.0, 44.5, 43.6, 29.9, 29.8, 26.4, 21.0, 21.0 ppm; IR (KBr) υ: 3728, 3026, 3001, 2985, 2846, 1785, 1723, 1526, 1426, 1401, 1362, 1123, 1023, 926, 845 cm $^{-1}$; HRMS (ESI-TOF) Calcd. for $C_{47}H_{40}Cl_2N_3O_4$ ([M+H]+): 780.2390, Found: 780.2395.

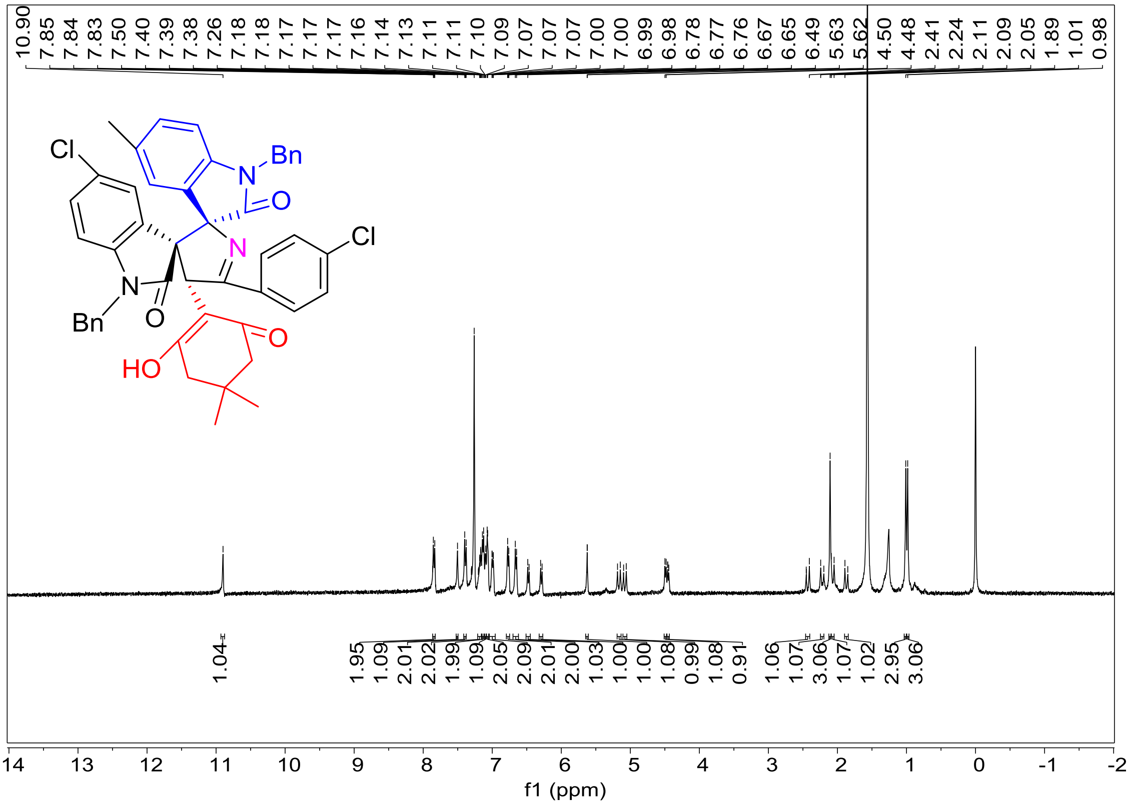

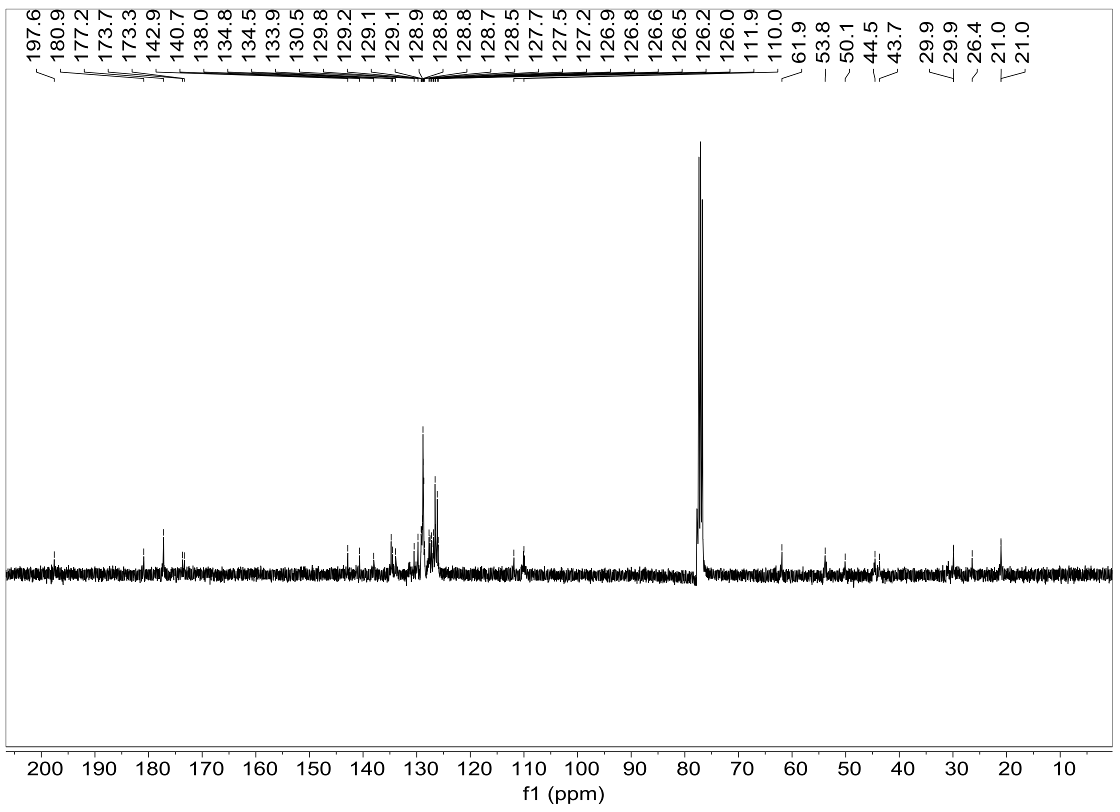
197.6
180.9
177.2
173.7
173.3
142.9
140.7
138.0
134.8
134.5
133.9
130.5
129.8
129.2
129.1
129.1
128.9
128.8
128.8
128.7
128.5
127.7
127.5
127.2
126.9
126.8
126.6
126.5
126.2
126.0
111.9
110.0
61.9
53.8
50.1
44.5
43.7
29.9
29.9
26.4
21.0
21.0
200 190 180 170 160 150 140 130 120 110 100 90 80 70 60 50 40 30 20 10
f1 (ppm)

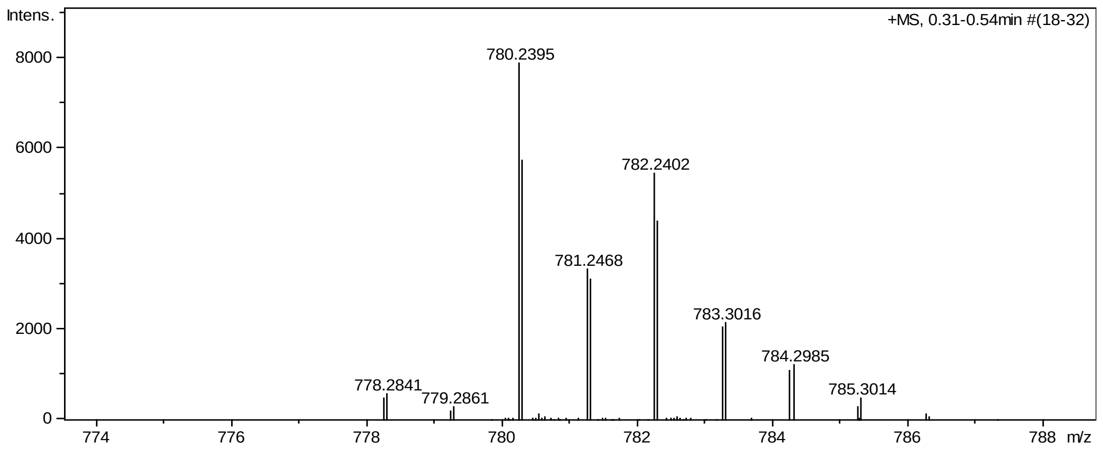
Intens.
+MS, 0.31-0.54min #(18-32)
8000
6000
4000
2000
0
780.2395
782.2402
781.2468
783.3016
784.2985
778.2841
779.2861
785.3014
774 776 778 780 782 784 786 788 m/z

***rel*-(3*R*,3'*S*,4'*R*)-1,1''-Dibenzyl-5'-(4-chlorophenyl)-4'-(2-hydroxy-4,4-dimethyl-6-oxocyclohex-1-en-1-yl)-5,5''-dimethyl-4'*H*-dispiro[indoline-3,2'-pyrrole-3',3''-indoline]-2,2''-dione (4f):** White solid, 51%, m.p. 265-266ºC; ^{1}H NMR (400 MHz,$CDCl_3$) δ: 11.02 (s, 1H, OH), 7.85 (d, *J* = 8.0Hz, 2H, ArH), 7.52 (s, 1H, ArH), 7.38 (d, *J* = 8.0 Hz, 2H, ArH), 7.16 - 7.11 (m, 2H, ArH), 7.07 - 7.02 (m, 4H, ArH), 6.95 (d, *J* = 8.8 Hz, 1H, ArH), 6.85 (s, 1H, ArH), 6.82 (d, *J* = 8.4 Hz, 1H, ArH), 6.66 (d, *J* = 7.6 Hz, 1H, ArH), 6.58 (d, *J* = 7.6 Hz, 2H, ArH), 6.38 (d, *J* = 8.0 Hz, 1H, ArH), 6.27 (d, *J* = 7.6 Hz, 1H, ArH), 5.68 (s, 1H, CH), 5.16 (d, *J* = 4.8 Hz, 1H, CH_2), 5.12 (d, *J* = 4.4, 1H, CH_2), 4.47 (d, *J* = 16.8 Hz, 1H, CH_2), 4.40 (d, *J* = 16.4 Hz, 1H, CH_2), 2.35 (d, *J* = 18.8 Hz, 1H, CH_2), 2.22 (d, *J* = 18.4 Hz, 1H, CH_2), 2.08 (s, 3H, CH_3), 2.04 (s, 1H, CH_2), 1.97 (s, 3H, CH_3),1.82 (d, *J* = 16.4 Hz, 1H, CH_2), 0.97 (s, 6H, CH_3) ppm; ^{13}C NMR (400 MHz, $CDCl_3$) δ: 197.2, 181.1, 177.5, 177.4, 172.7, 141.8, 140.6, 137.7, 135.2, 134.3, 133.9, 131.4, 131.3, 130.2, 129.7, 129.1, 128.7, 128.6, 128.4, 127.3, 127.0, 126.9, 126.6, 126.1, 124.0, 112.0, 109.6, 108.9, 87.6, 61.9, 53.2, 50.0, 44.6, 44.1, 43.5, 30.8, 29.7, 26.5, 21.0, 20.9 ppm; IR (KBr) υ: 3712, 3026, 2972, 2866, 1842, 1729, 1617, 1527, 1321, 1293, 1151, 1028, 962, 811 cm^{-1}; HRMS (ESI-TOF) Calcd. for $C_{48}H_{43}ClN_3O_4$ ($[M+H]^+$): 760.2937, Found: 760.2930.

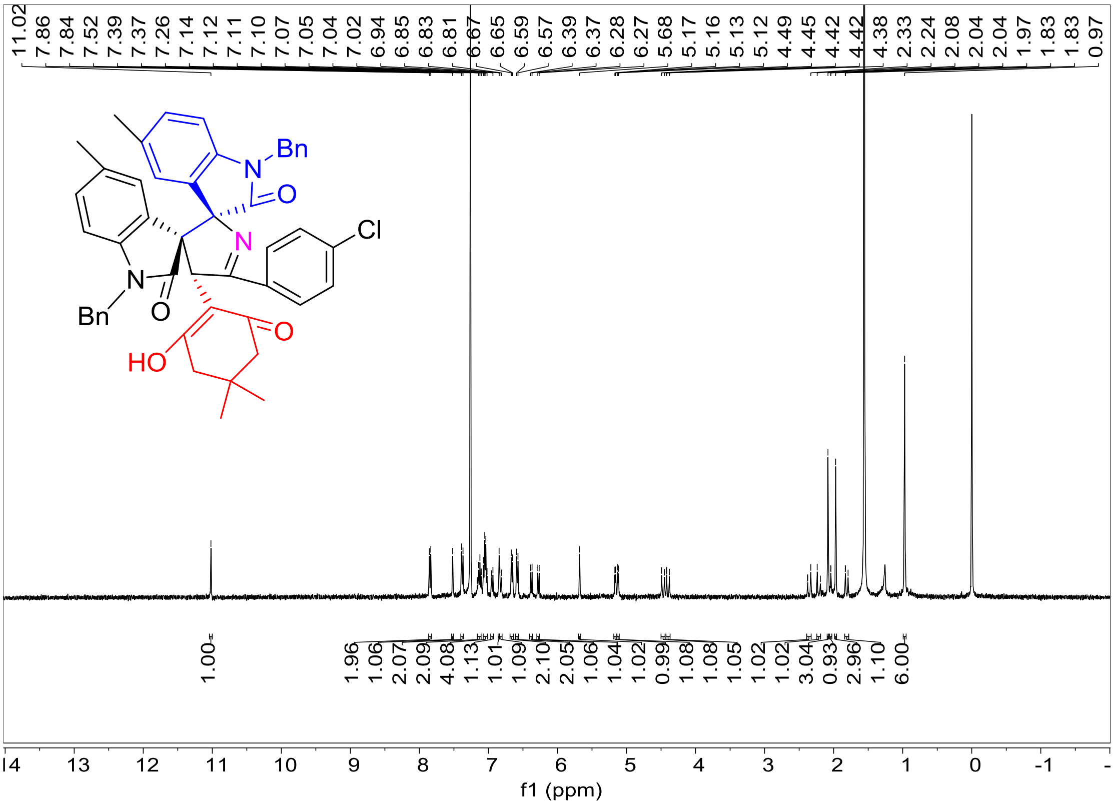

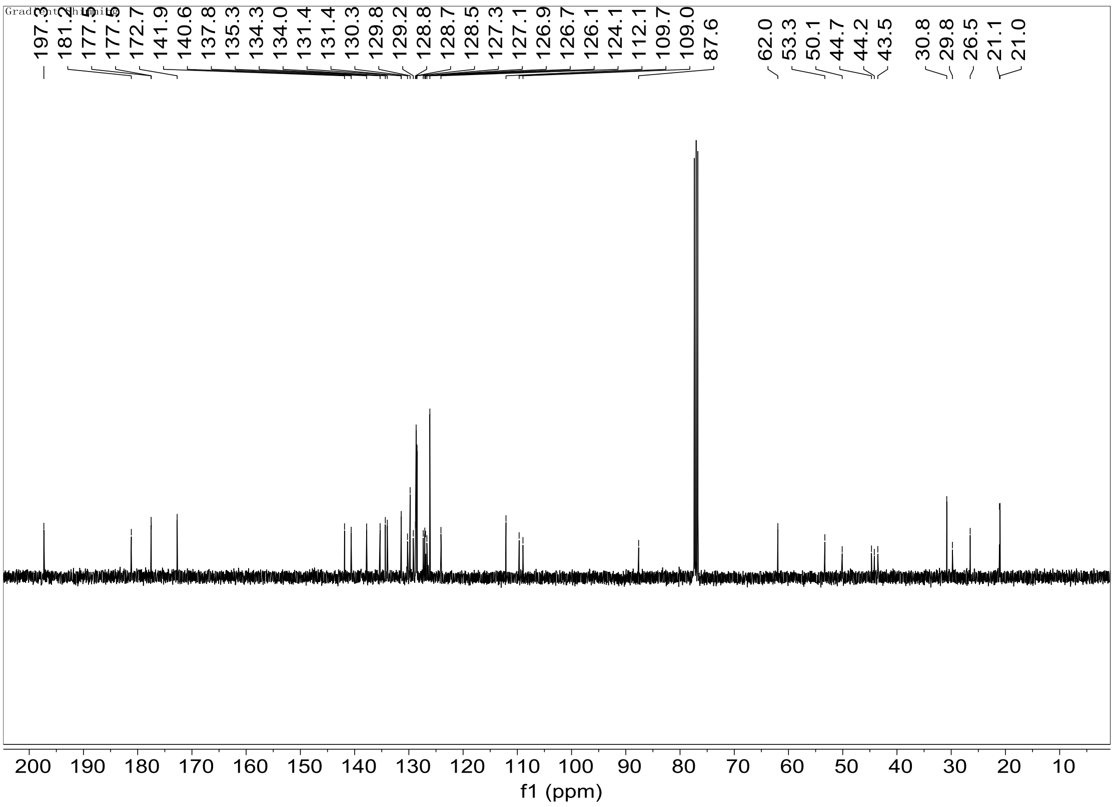
197.3
181.2
177.5
177.5
172.7
141.9
140.6
137.8
135.3
134.3
134.0
131.4
131.4
130.3
129.8
129.2
128.8
128.7
128.5
127.3
127.1
126.9
126.7
126.1
124.1
112.1
109.7
109.0
87.6
62.0
53.3
50.1
44.7
44.2
43.5
30.8
29.8
26.5
21.1
21.0
200 190 180 170 160 150 140 130 120 110 100 90 80 70 60 50 40 30 20 10
f1 (ppm)

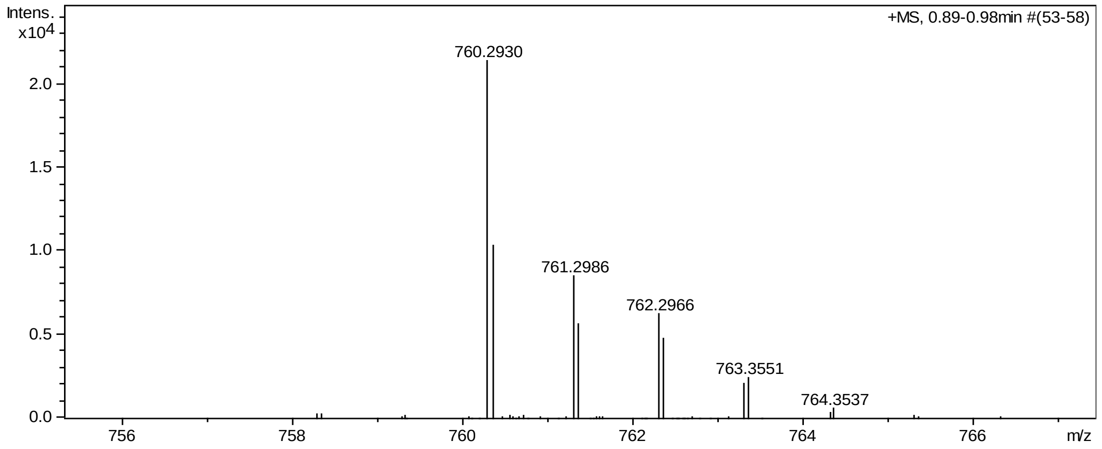
Intens.
x10^4
+MS, 0.89-0.98min #(53-58)
760.2930
761.2986
762.2966
763.3551
764.3537
2.0
1.5
1.0
0.5
0.0
756
758
760
762
764
766
m/z

***rel*-(3*R*,3'*S*,4'*R*)-1''-Benzyl-5''-chloro-4'-(2-hydroxy-4,4-dimethyl-6-oxocyclohex-1-en-1-yl)-5'-(*p*-tolyl)-4'*H*-dispiro[indoline-3,2'-pyrrole-3',3''-indoline]-2,2''-dione (4g):** White solid, 34%, m.p. 279-280°C; ^{1}H NMR (400 MHz, $CDCl_3$) δ: 10.48 (s, 1H, OH), 7.77 (d, *J* = 8.4 Hz, 2H, ArH),7.67 (d, *J* = 7.6 Hz, 1H, ArH), 7.42 (s, 1H, NH), 7.29 (s, 2H, ArH), 7.20 (d, *J* = 8.0 Hz, 2H, ArH), 7.16 - 7.11 (m, 3H, ArH), 7.04 (s, 1H, ArH), 7.01 - 6.99 (m, 1H, ArH), 6.78 (d, *J* =8.0 Hz, 1H, ArH), 6.66 (d, *J* = 7.6 Hz, 2H, ArH), 6.24 (d, *J* = 8.4, 1H, ArH), 5.61 (s, 1H, CH), 5.14 (d, *J* = 16.0 Hz, 1H, CH_2), 4.44 (d, *J* = 16.0 Hz, 1H, CH_2), 2.40 (d, *J* = 22.4, 1H, CH_2), 2.40 (s, 3H, CH_3),2.19 (d, *J* = 18.4 Hz, 1H, CH_2), 2.05 (d, *J* = 16.0 Hz, 1H, CH_2), 1.84 (d, *J* = 15.6 Hz, 1H, CH_2), 1.00 (s, 3H, CH_3), 0.98 (s, 3H, CH_3) ppm; ^{13}C NMR (101 MHz, $CDCl_3$) δ: 189.4, 183.4, 169.2, 161.1, 151.1, 141.4, 138.3, 137.8, 135.5, 135.3, 134.2, 133.1, 130.6, 128.7, 128.6, 128.4, 128.1, 127.8, 127.8, 127.5, 127.4, 127.2, 125.5, 124.5, 122.9, 115.0, 114.2, 113.7, 109.5, 90.3, 52.6, 43.6, 39.0, 33.9, 31.8, 28.6, 28.1, 21.5 ppm; IR (KBr) υ: 3726, 3023, 2982, 2847, 1856, 1745, 1687, 1521, 1329, 1025, 925, 829 cm^{-1}; HRMS (ESI-TOF) Calcd. for $C_{40}H_{35}ClN_3O_4$ ([M +H]$^+$): 656.2303, Found: 656.2305.

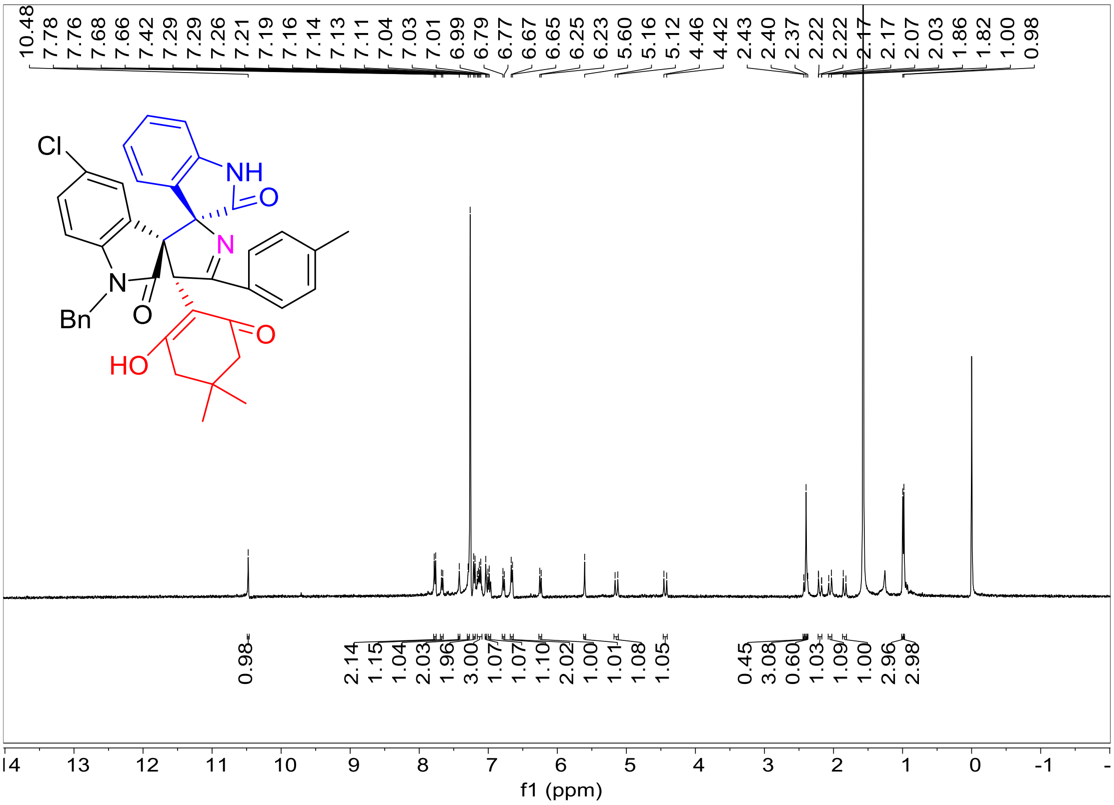

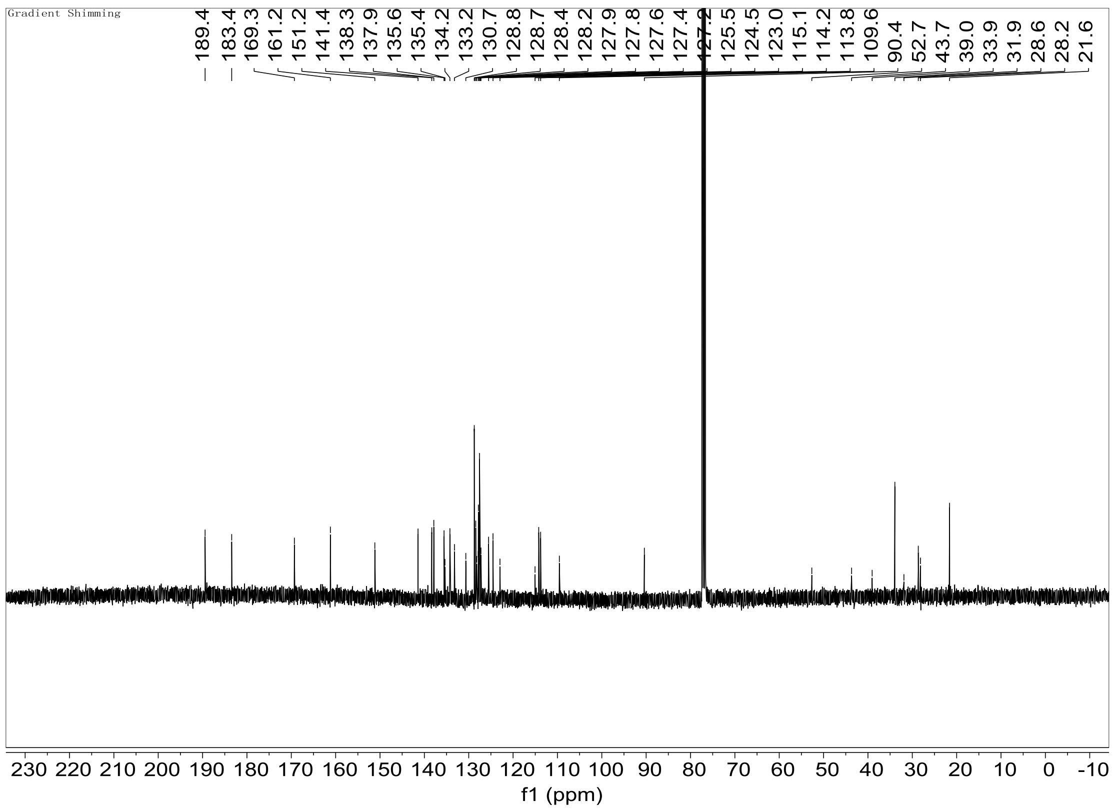
Gradient Shimming
189.4
183.4
169.3
161.2
151.2
141.4
138.3
137.9
135.6
135.4
134.2
133.2
130.7
128.8
128.7
128.4
128.2
127.9
127.8
127.6
127.4
127.2
125.5
124.5
123.0
115.1
114.2
113.8
109.6
90.4
52.7
43.7
39.0
33.9
31.9
28.6
28.2
21.6
230 220 210 200 190 180 170 160 150 140 130 120 110 100 90 80 70 60 50 40 30 20 10 0 -10
f1 (ppm)

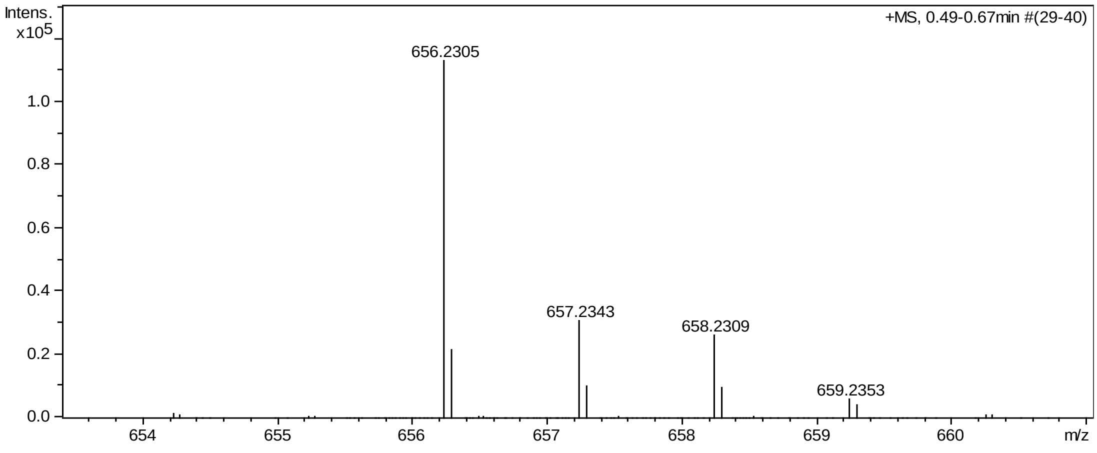
Intens.
x10^5
+MS, 0.49-0.67min #(29-40)
656.2305
657.2343
658.2309
659.2353
1.0
0.8
0.6
0.4
0.2
0.0
654
655
656
657
658
659
660
m/z

***rel*-(3*R*,3'*S*,4'*R*)-1-Benzyl-1''-butyl-5''-chloro-4'-(2-hydroxy-4,4-dimethyl-6-oxocyclohex-1-en-1-yl)-5'-(4-methoxyphenyl)-5-methyl-4'*H* -dispiro[indoline-3,2'-pyrrole-3',3''-indoline]-2,2''-dione (4h):** White solid, 65%, m.p. 228-230°C; ^{1}H NMR (400 MHz,$CDCl_3$) δ: 10.87 (s, 1H, OH), 7.83 (d, *J* = 6.8 Hz, 2H, ArH), 7.51 (s, 1H, ArH), 7.20 - 7.12 (m, 4H, ArH), 7.06 (s, 1H, ArH), 6.92 - 6.88 (m, 3H, ArH), 6.80 - 6.79 (m, 2H, ArH), 6.54 (d, *J* = 8.0 Hz, 1H, ArH), 6.40 (d, *J* = 8.8, 1H, ArH), 5.53 (s, 1H, CH), 5.09 (d, *J* = 16.8, 1H, CH_2), 4.41 (d, *J* = 17.2 Hz, 1H, CH_2), 3.85 (s, 3H, OCH_3), 3.72 (s, 1H, CH_2), 3.32 (s, 1H, CH_2), 2.43 (d, *J* = 20.4 Hz, 1H, CH_2), 2.25 (s, 3H, CH_3), 2.19 (s, 1H, CH_2), 2.05 (d, *J* = 16.0 Hz, 1H, CH_2), 1.87 (d, *J* = 16.4 Hz, 1H, CH_2), 1.36 (s, 2H, CH_2), 1.09 (s, 2H, CH_2), 1.00 (s, 3H, CH_3), 0.99 (s, 3H, CH_3), 0.81 (t, *J* = 6.4 Hz, 3H, CH_3) ppm; ^{13}C NMR (400 MHz, $CDCl_3$) δ: 197.6, 180.9, 177.5, 177.0, 173.0, 162.4, 143.3, 140.5, 134.1, 133.6, 130.2, 130.1, 128.7, 128.4, 127.6, 126.9, 126.9, 126.6, 126.4, 126.1, 125.6, 113.7, 112.1, 108.8, 87.0, 61.3, 55.4, 55.3, 53.5, 53.4, 50.1, 44.5, 40.0, 30.7, 29.9, 29.3, 26.3, 21.1, 19.9, 13.7 ppm; IR (KBr) υ: 3689, 3068, 3019, 2956, 2847, 1872, 1742, 1652, 1526, 1126, 1059, 971, 863 cm^{-1}; HRMS (ESI-TOF) Calcd. for $C_{45}H_{45}ClN_3O_5$ ($[M+H]^+$): 742.3042, Found: 742.3027.

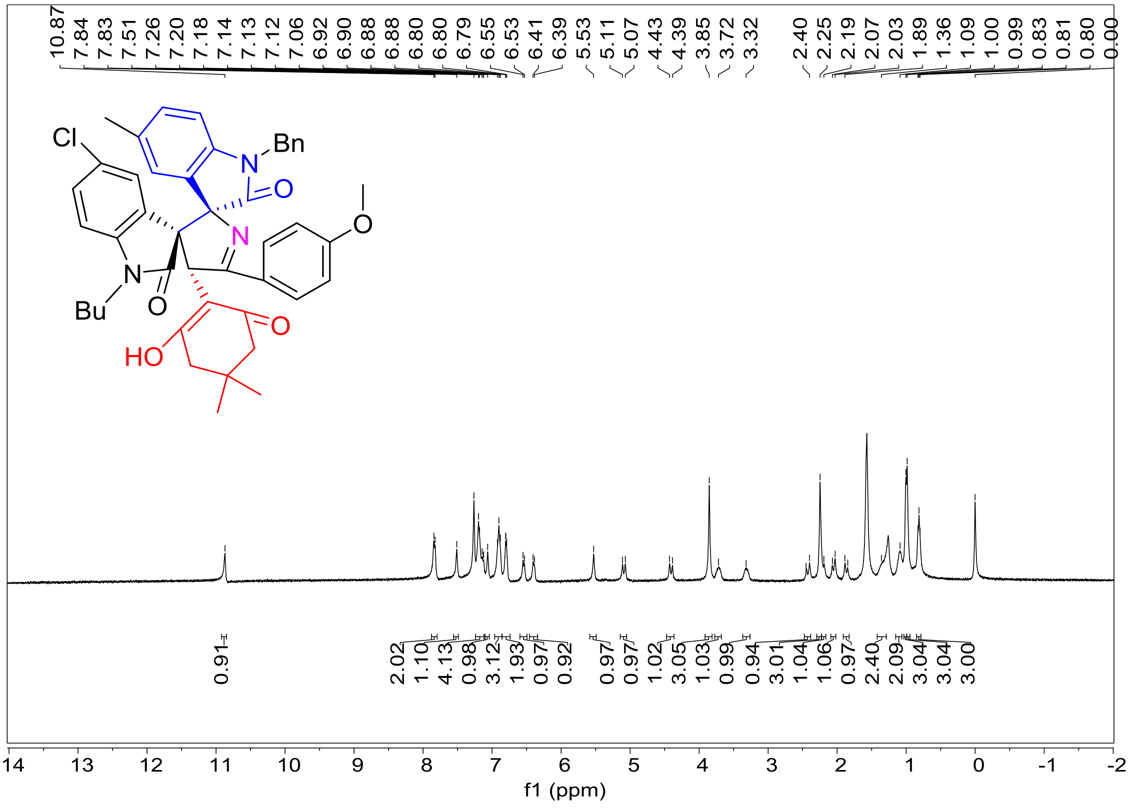

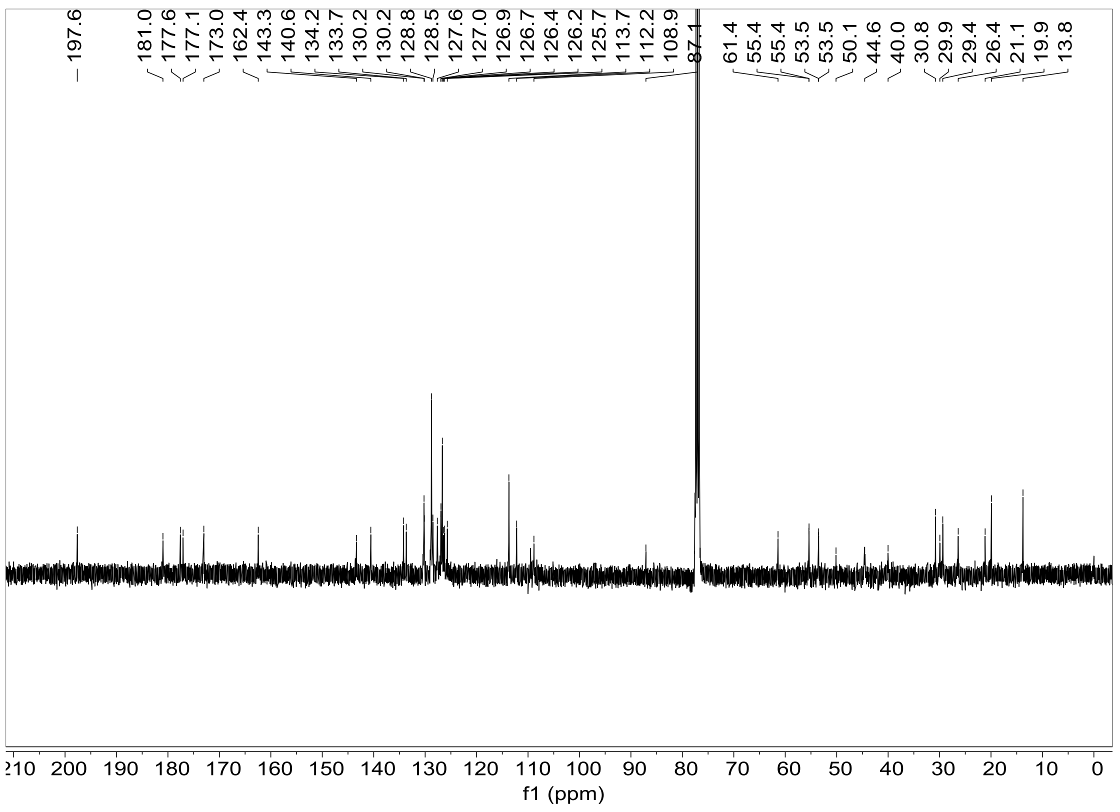
197.6
181.0
177.6
177.1
173.0
162.4
143.3
140.6
134.2
133.7
130.2
130.2
128.8
128.5
127.6
127.0
126.9
126.7
126.4
126.2
125.7
113.7
112.2
108.9
87.1
61.4
55.4
55.4
53.5
53.5
50.1
44.6
40.0
30.8
29.9
29.4
26.4
21.1
19.9
13.8
210 200 190 180 170 160 150 140 130 120 110 100 90 80 70 60 50 40 30 20 10 0
f1 (ppm)

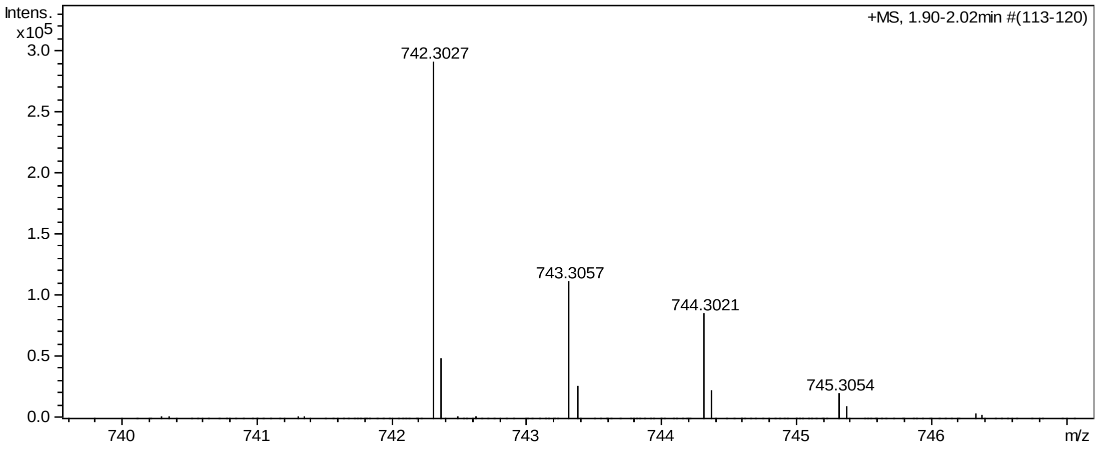
Intens.
x10^5
+MS, 1.90-2.02min #(113-120)
742.3027
743.3057
744.3021
745.3054
m/z

***rel*-(3*R*,3'*S*,4'*R*)-1-Benzyl-5''-chloro-4'-(2-hydroxy-4,4-dimethyl-6-oxocyclohex-1-en-1-yl)-5'-(4-methoxyphenyl)-5-methyl-4'*H*-dispiro[indoline-3,2'-pyrrole-3',3''-indoline]-2,2''-dione (4i):** White solid, 62%, m.p. 226-225°C; ^{1}H NMR (400 MHz,$CDCl_3$) δ: 10.86 (s, 1H, OH), 7.85 - 7.83 (m, 2H, ArH), 7.52 (s, 1H, NH), 7.20 - 7.16 (m, 3H, ArH), 7.11 - 7.06 (m, 2H, ArH), 6.96 - 6.88 (m, 3H, ArH), 6.78 - 6.77 (m, 2H, ArH), 6.58 - 6.56 (m, 1H, ArH), 6.45 - 6.42 (m, 1H, ArH), 5.60 (s, 1H, CH), 5.15 (d, *J* = 16.0 Hz, 1H, CH_2), 4.39 (d, *J* = 16.0, 1H, CH_2), 4.39 (d, *J* = 16.0 Hz, 1H, CH_2), 4.39 (d, *J* = 16.0 Hz, 1H, CH_2), 3.86 (s, 3H, OCH_3), 2.42 (d, *J* = 17.2 Hz, 1H, CH_2), 2.21 - 2.20 (m, 1H, CH_2), 2.10 (s, 1H, CH_3), 2.05 (s, 1H, CH_2), 1.87 (d, *J* = 16.0 Hz, 1H, CH_2), 2.23(s, 3H, CH_3), 2.06 (d, *J* = 16.0 Hz, 1H, CH_2), 1.87 (d, *J* = 16.4 Hz, 1H, CH_2), 1.00 (s, 6H, CH_3) ppm; ^{13}C NMR (101 MHz, $CDCl_3$) δ: 197.7, 178.9, 177.2, 173.3, 162.4, 140.7, 140.5, 134.0, 134.0, 130.3, 130.1, 128.7, 128.5, 128.4, 127.6, 127.1, 126.9, 126.7, 126.5, 126.5, 125.5, 113.7, 112.0, 110.7, 109.6, 61.9, 55.3, 53.7, 50.0, 45.8, 44.5, 44.4, 30.8, 29.9, 26.3, 21.1 ppm; IR (KBr) υ: 3765, 3521, 3056, 3002, 2963, 2815, 1864, 1749, 1682, 1537, 1352, 1145, 1039, 921, 882 cm^{-1}; HRMS (ESI-TOF) Calcd. for $C_{41}H_{37}ClN_3O_5$ ($[M+H]^+$): 686.2416, Found: 686.2413.

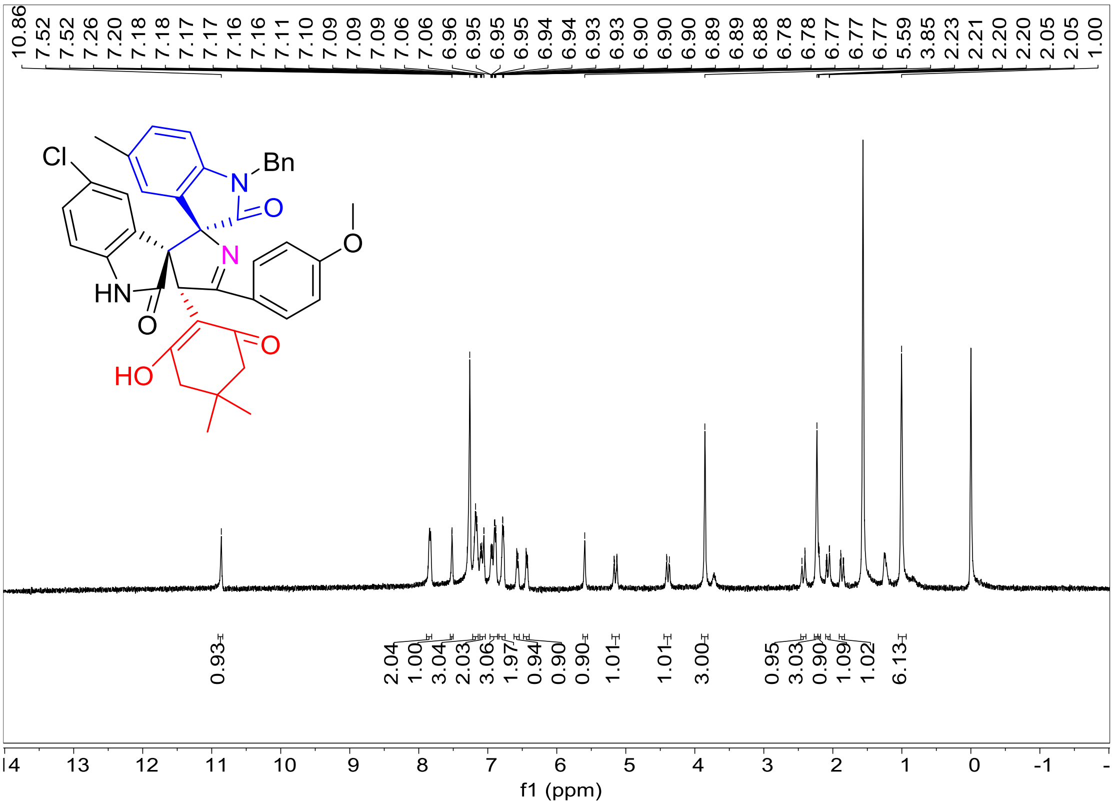

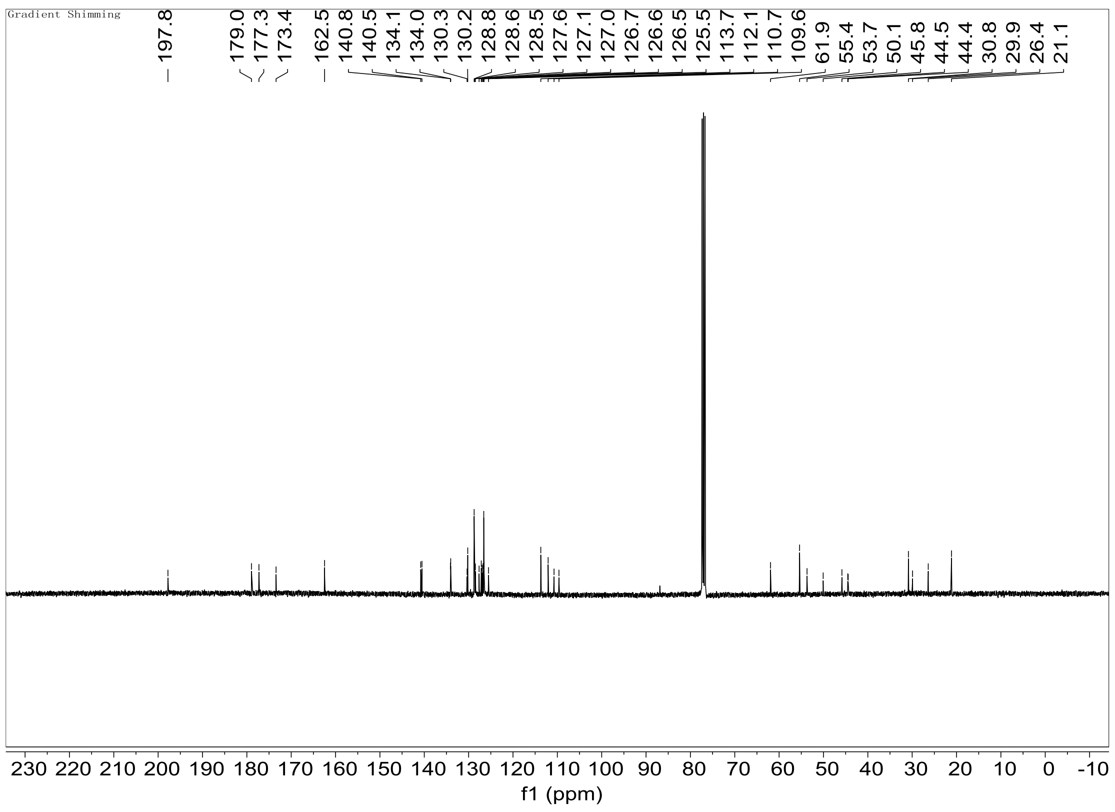
Gradient Shimming
197.8
179.0
177.3
173.4
162.5
140.8
140.5
134.1
134.0
130.3
130.2
128.8
128.6
128.5
127.6
127.1
127.0
126.7
126.6
126.5
125.5
113.7
112.1
110.7
109.6
61.9
55.4
53.7
50.1
45.8
44.5
44.4
30.8
29.9
26.4
21.1
230 220 210 200 190 180 170 160 150 140 130 120 110 100 90 80 70 60 50 40 30 20 10 0 -10
f1 (ppm)

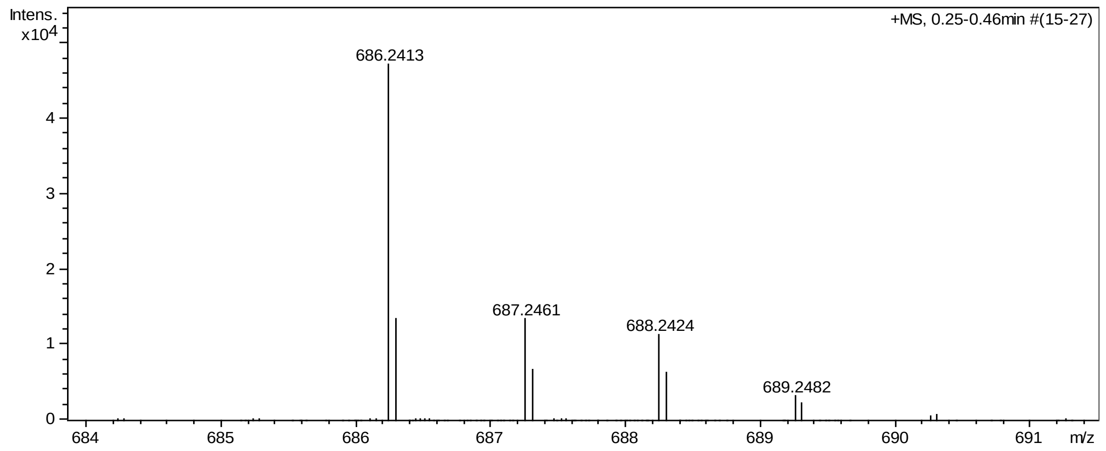
Intens.
x10^4
+MS, 0.25-0.46min #(15-27)
686.2413
687.2461
688.2424
689.2482
0
1
2
3
4
684
685
686
687
688
689
690
691
m/z

***rel*-(3*R*,3'*S*,4'*R*)-1,1''-dibenzyl-5''-chloro-4'-(2-hydroxy-6-oxocyclohex-1-en-1-yl)-5-methyl-5'-(*p*-tolyl)-4'*H*-dispiro[indoline-3,2'-pyrrole-3',3''-indoline]-2,2''-dione (4j):** White solid, 71%, m.p. 235-236ºC; ^{1}H NMR (400 MHz,$CDCl_3$) δ: 10.90 (s, 1H, OH), 7.82 (d, *J* = 8.0 Hz, 2H, ArH),7.21 (d, *J* = 8.0 Hz, 2H, ArH), 7.17 (d, *J* = 7.2 Hz, 1H, ArH), 7.14 - 7.11 (m, 2H, ArH), 7.09 - 7.05 (m, 3H, ArH), 7.00 - 6.97 (m, 2H, ArH), 6.76 (d, *J* = 7.2 Hz, 2H, ArH), 6.65 (d, *J* = 7.2 Hz, 2H, ArH), 6.46 (d, *J* = 8.0 Hz, 1H, ArH), 6.28 (d, *J* = 8.4 Hz, 1H, ArH), 5.58 (s, 1H, CH), 5.20 (d, *J* = 16.4 Hz, 1H, CH_2), 5.08 (d, *J* = 16.0 Hz, 1H, CH_2), 4.47 (d, *J* = 6.8 Hz, 1H, CH_2), 4.43 (d, *J* = 8.2 Hz, 1H, CH_2), 2.57 - 2.49 (m, 1H, CH_2), 2.40 (d, *J* = 18.0 Hz, 1H, CH_2), 2.41 (s, 3H, CH_3), 2.27 (d, *J* = 16.0 Hz,, 1H, CH_2), 2.10 (s, 3H, CH_3), 1.94 - 1.77 (m, 3H, CH_2) ppm; ^{13}C NMR (400 MHz, $CDCl_3$) δ: 197.8, 181.5, 177.4, 177.2, 174.9, 142.8, 142.2, 140.6, 134.8, 134.3, 134.0, 130.2, 130.0, 129.1, 128.8, 128.7, 128.7, 128.5, 128.3, 127.5, 127.2, 127.1, 126.5, 126.2, 126.1, 125.9, 113.5, 109.8, 109.8, 109.7, 109.7, 87.1, 61.9, 54.0, 44.4, 43.6, 43.6, 36.4, 31.0, 21.6, 21.6, 21.0, 20.9, 19.9 ppm; IR (KBr) υ: 3692, 3029, 2993, 2862, 1872, 1739, 1668, 1552, 1421, 1192, 1029, 952, 833, 724 cm^{-1}; HRMS (ESI-TOF) Calcd. for $C_{46}H_{38}ClN_3O_4$ ($[M+H]^+$): 732.2624, Found: 732.2612.

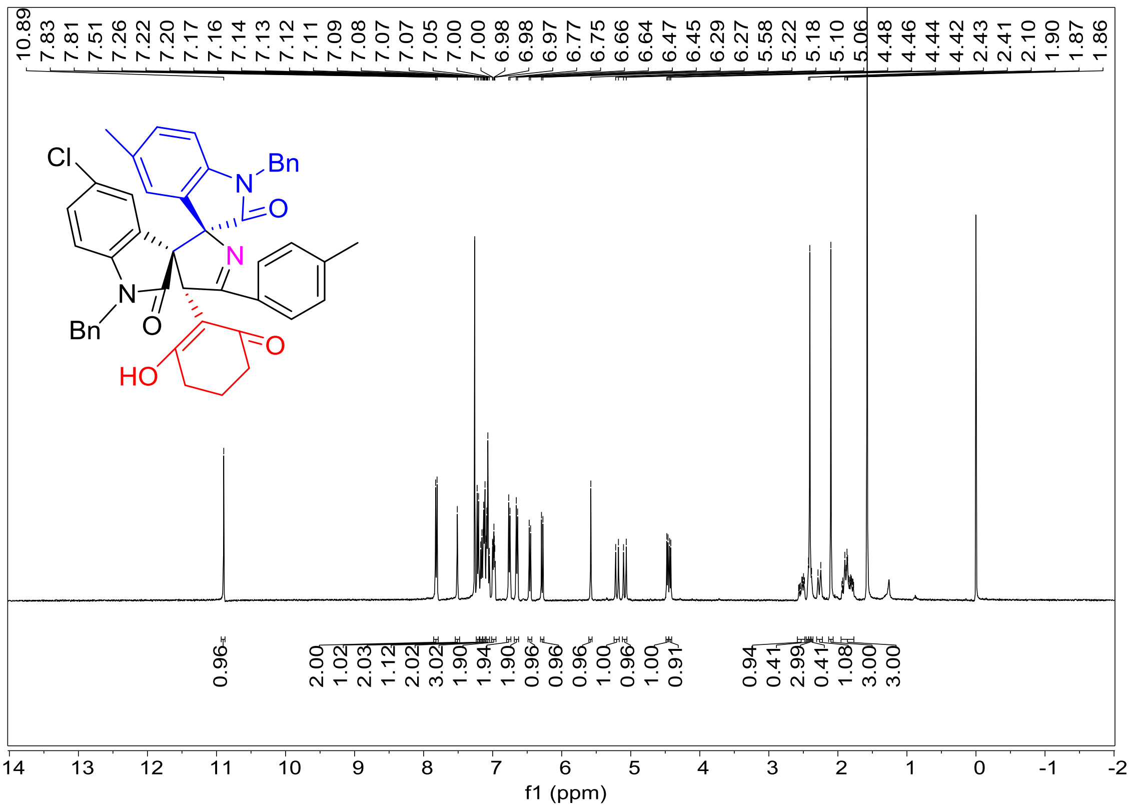

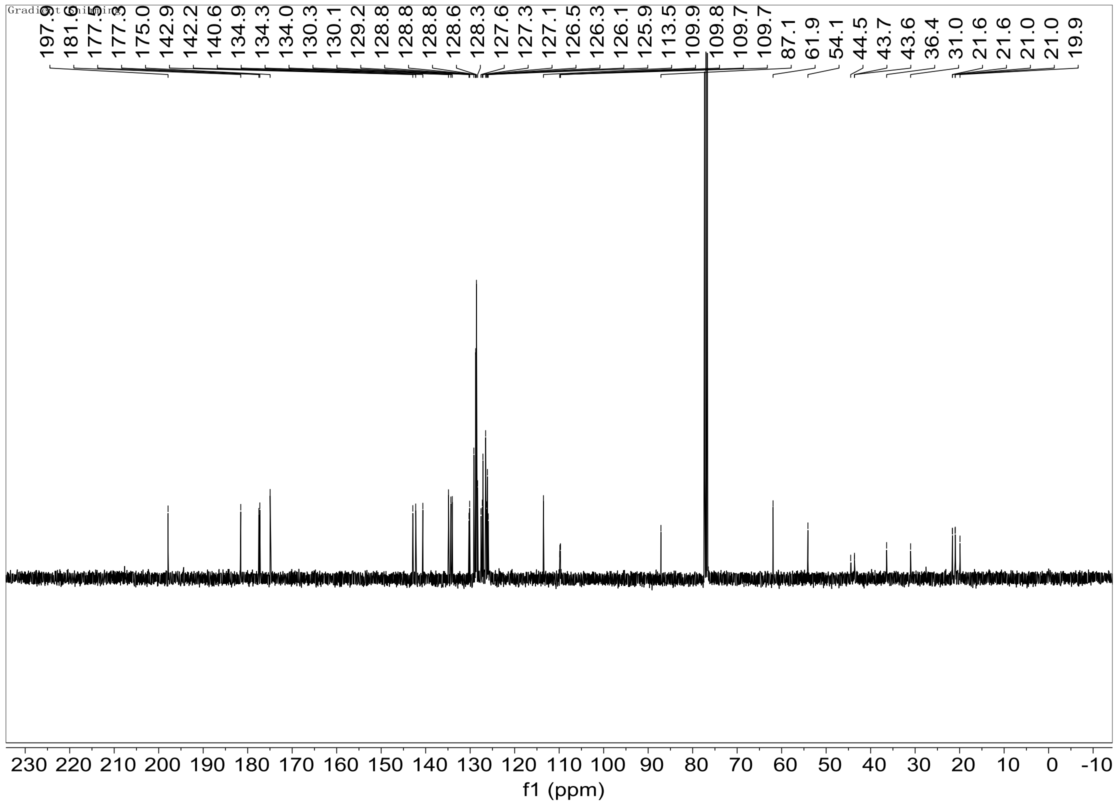
197.9
181.6
177.5
177.3
175.0
142.9
142.2
140.6
134.9
134.3
134.0
130.3
130.1
129.2
128.8
128.8
128.8
128.6
128.3
127.6
127.3
127.1
126.5
126.3
126.1
125.9
113.5
109.9
109.8
109.7
109.7
87.1
61.9
54.1
44.5
43.7
43.6
36.4
31.0
21.6
21.6
21.0
21.0
19.9
230 220 210 200 190 180 170 160 150 140 130 120 110 100 90 80 70 60 50 40 30 20 10 0 -10
f1 (ppm)

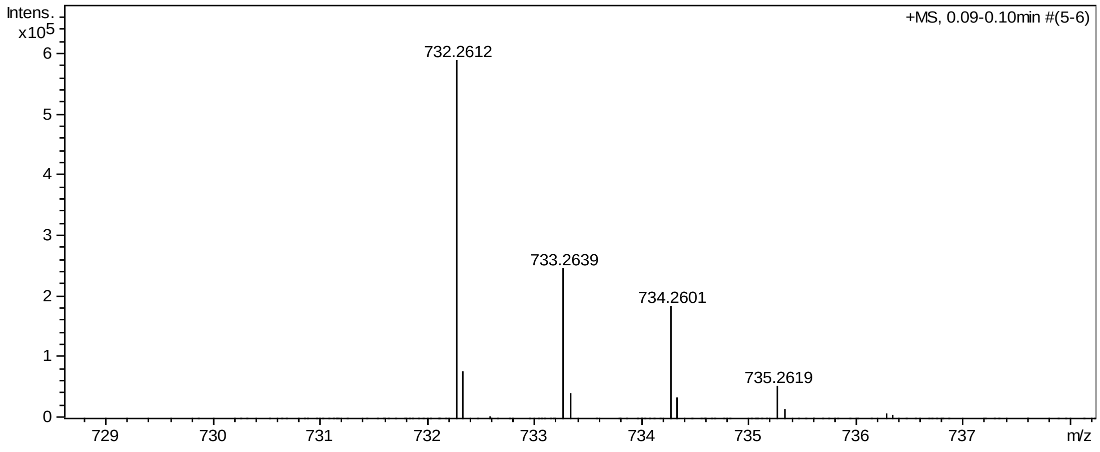
Intens.
x10^5
+MS, 0.09-0.10min #(5-6)
732.2612
733.2639
734.2601
735.2619
729 730 731 732 733 734 735 736 737 m/z

***rel*-(3*R*,3'*S*,4'*R*)-1,1''-Dibenzyl-5,5''-dichloro-4'-(2-hydroxy-6-oxocyclohex-1-en-1-yl)-5'-(*p*-tolyl)-4'*H*-dispiro[indoline-3,2'-pyrrole-3',3''-indoline]-2,2''-dione (4k):** White solid, 68%, m.p. 221-222°C; ^{1}H NMR (400 MHz,$CDCl_3$) δ: 10.60 (s, 1H, OH), 7.80 (d, *J* = 8.4 Hz, 2H, ArH), 7.73 (d, *J* = 2.0 Hz, 1H, ArH), 7.22 (d, *J* = 8.0 Hz, 2H, ArH), 7.19 - 7.11 (m, 7H, ArH), 7.06 - 7.01 (m, 2H, ArH), 6.84 - 6.82 (m, 2H, ArH), 6.75 (d, *J* = 7.2 Hz, 2H, ArH), 6.48 (d, *J* = 8.4, 1H, ArH), 6.37 (d, *J* = 8.0, 1H, ArH), 5.58 (s, 1H, CH), 5.12 (t, *J* = 15.6 Hz, 2H, CH_2), 4.50 (d, *J* = 16.0, 1H, CH_2), 4.44 (d, *J* = 16.0 Hz, 1H, CH_2), 2.57 - 2.49 (m, 1H, CH_2), 2.44 - 2.38 (m, 1H, CH_2), 2.41 (s, 3H, CH_3), 2.30 - 2.25 (m, 1H, CH_2), 1.93 - 1.82 (m, 3H, CH_2) ppm; ^{13}C NMR (600 MHz, $CDCl_3$) δ: 197.7, 192.0, 177.3, 174.8, 142.9, 142.5, 141.6, 134.7, 133.5, 130.0, 129.2, 128.9, 128.8, 128.7, 128.6, 128.3, 127.8, 127.4, 126.5, 126.4, 125.9, 113.4, 110.9, 110.0, 86.7, 61.8, 54.3, 44.6, 43.9, 36.4, 31.0, 21.6, 19.9 ppm; IR (KBr) υ: 3682, 3041, 2953, 2851, 1834, 1774, 1689, 1541, 1328, 1128, 1057, 952, 894, 832 cm^{-1}; HRMS (ESI-TOF) Calcd. for $C_{45}H_{36}Cl_2N_3O_4$ ($[M+H]^+$): 752.2077, Found: 752.2060.

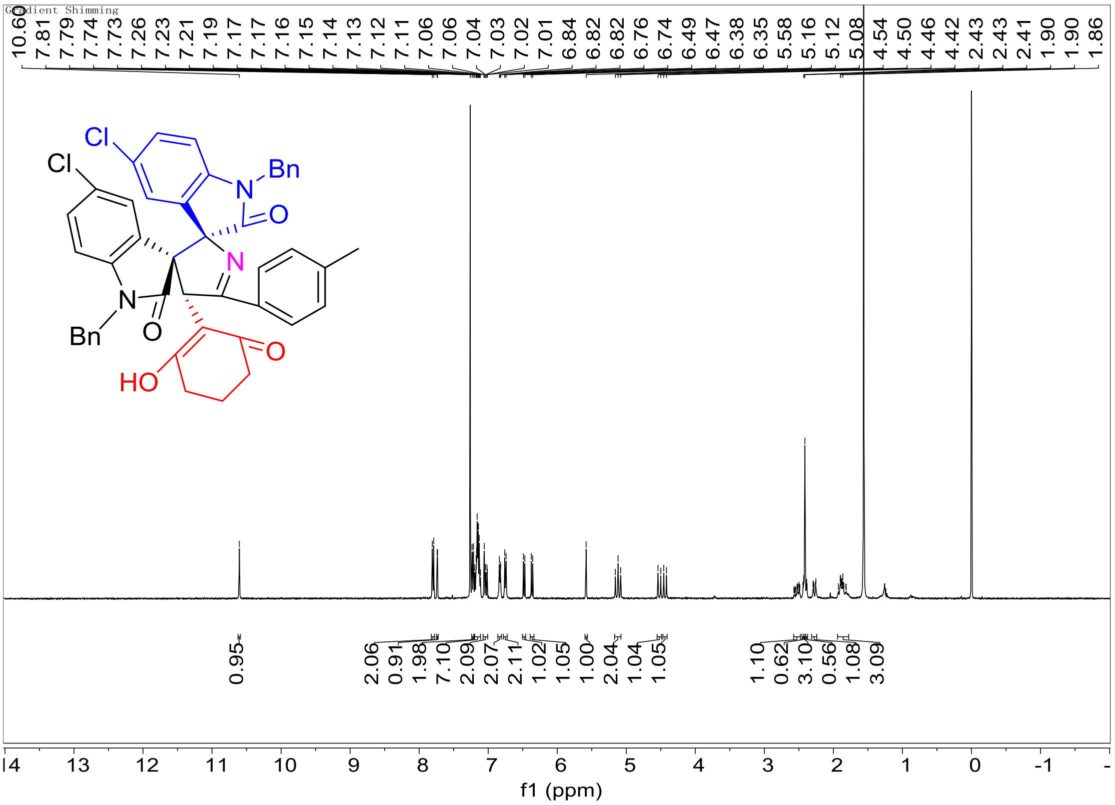

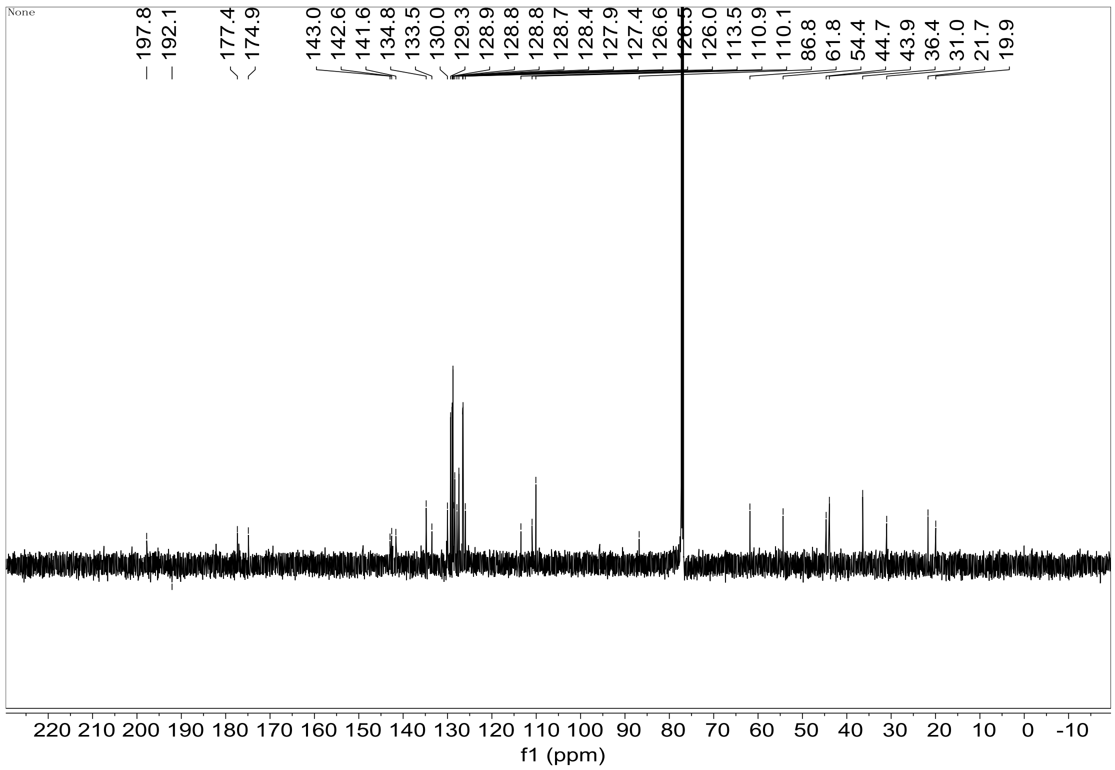
None
197.8
192.1
177.4
174.9
143.0
142.6
141.6
134.8
133.5
130.0
129.3
128.9
128.8
128.8
128.7
128.4
127.9
127.4
126.6
126.5
126.0
113.5
110.9
110.1
86.8
61.8
54.4
44.7
43.9
36.4
31.0
21.7
19.9
220 210 200 190 180 170 160 150 140 130 120 110 100 90 80 70 60 50 40 30 20 10 0 -10
f1 (ppm)

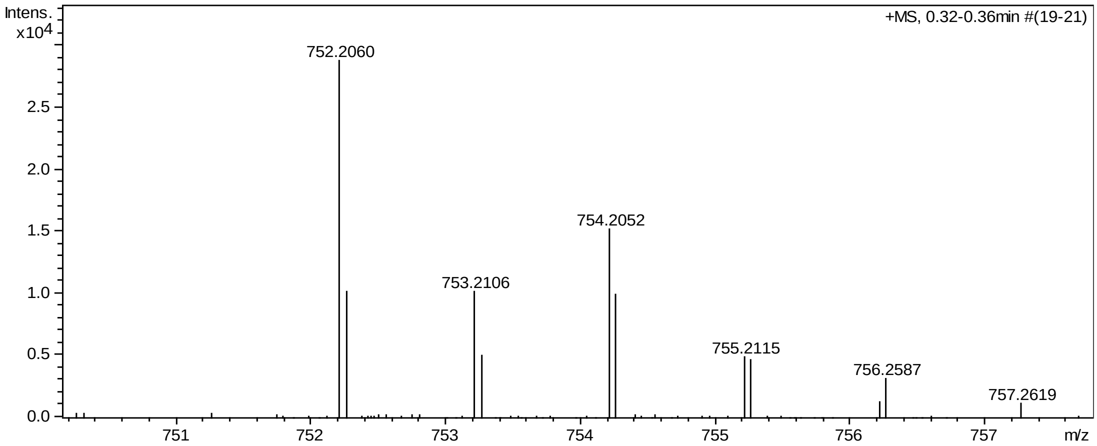
Intens.
x10^4
+MS, 0.32-0.36min #(19-21)
752.2060
754.2052
753.2106
755.2115
756.2587
757.2619
2.5
2.0
1.5
1.0
0.5
0.0
751
752
753
754
755
756
757
m/z

***rel*-(3*R*,3'*S*,4'*R*)-1,1''-Dibenzyl-5''-chloro-4'-(2-hydroxy-6-oxocyclohex-1-en-1-yl)-5'-(*p*-tolyl)-4'*H*-dispiro[indoline-3,2'-pyrrole-3',3''-indoline]-2,2''-dione (4l):** White solid, 64%, m.p. 263-264°C; ^{1}H NMR (400 MHz,$CDCl_3$) δ: 10.79 (s, 1H, OH), 7.81 (d, *J* = 7.6 Hz, 2H, ArH), 7.69 (d, *J* = 7.2 Hz, 1H, ArH), 7.22 (d, *J* = 8.4 Hz, 2H, ArH), 7.19 - 7.15 (m, 2H, ArH), 7.13 - 7.07 (m, 6H, ArH), 6.98 (d, *J* = 5.6 Hz, 2H, ArH), 6.74 (d, *J* = 7.2 Hz, 2H, ArH), 6.66 (d, *J* = 7.2, 2H, ArH), 6.57 (d, *J* = 8.0 Hz, 1H, ArH), 6.26 (d, *J* = 8.4 Hz, 1H, ArH), 5.60 (s, 1H, CH), 5.14 (d, *J* = 16.4 Hz, 1H, CH_2), 5.10 (d, *J* = 16.8 Hz, 1H, CH_2), 4.47 (t, *J* = 16.0 Hz, 2H, CH_2), 2.57 - 2.49 (m, 1H, CH_2), 2.41 (s, 3H, CH_3), 2.38 (s, 1H, CH_2), 2.27 (d, *J* = 16.0 Hz, 1H, CH_2), 1.94 - 1.81 (m, 3H, CH_2) ppm; ^{13}C NMR (600 MHz, $CDCl_3$) δ: 197.7, 181.6, 177.6, 177.2, 174.8, 143.1, 142.7, 142.3, 134.7, 133.9, 130.0, 130.0, 129.2, 128.8, 128.6, 128.3, 128.3, 128.1, 127.6, 127.6, 127.3, 127.2, 127.1, 126.4, 126.3, 126.2, 126.0, 124.4, 113.5, 110.0, 87.1, 61.9, 54.1, 44.4, 43.7, 36.4, 31.0, 21.6, 19.9, 19.9 ppm; IR (KBr) υ: 3745, 3062, 3002, 2994, 2953, 1863, 1742, 1702, 1685, 1563, 1375, 1169, 1056, 961, 842 cm^{-1}; HRMS (ESI-TOF) Calcd. for $C_{45}H_{37}ClN_3O_4$ ($[M+H]^+$): 718.2467, Found: 718.2452.

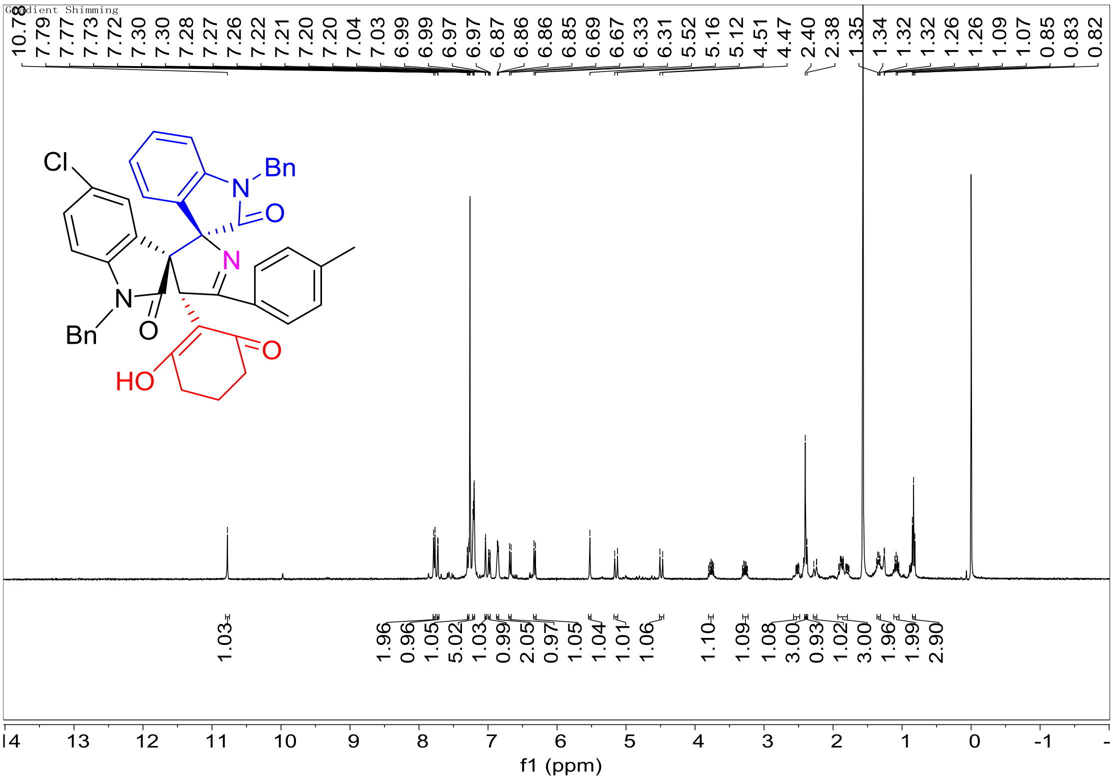

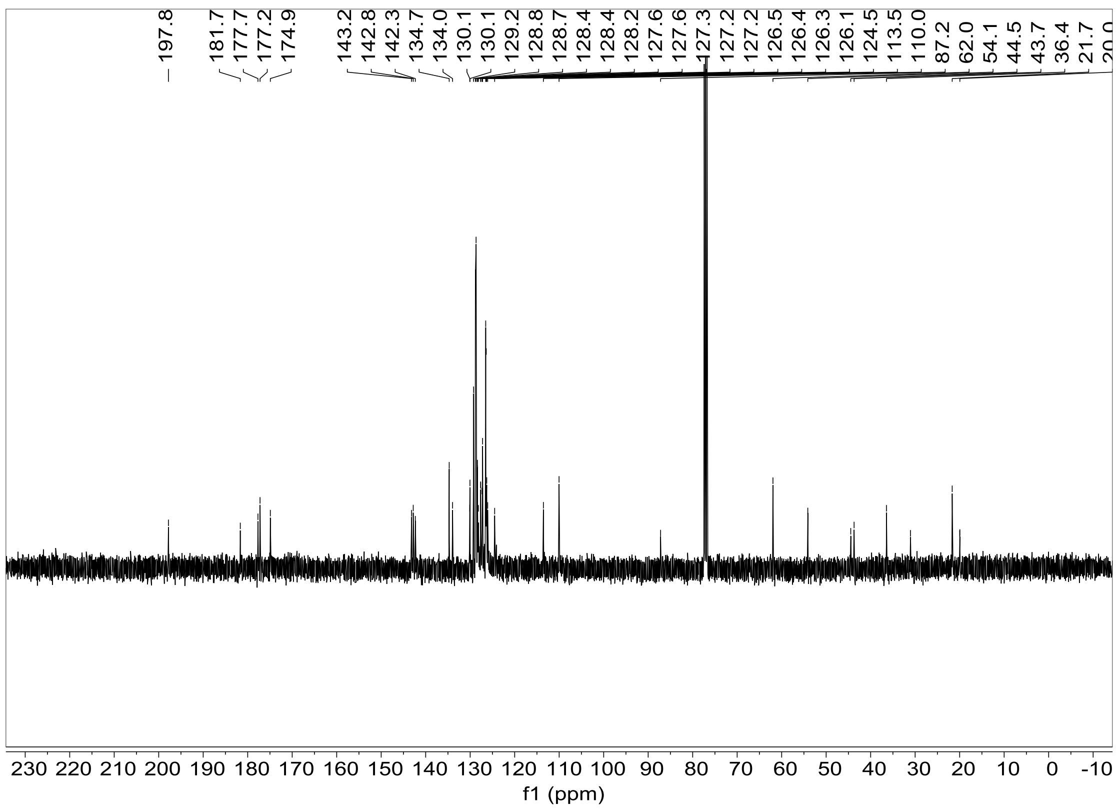

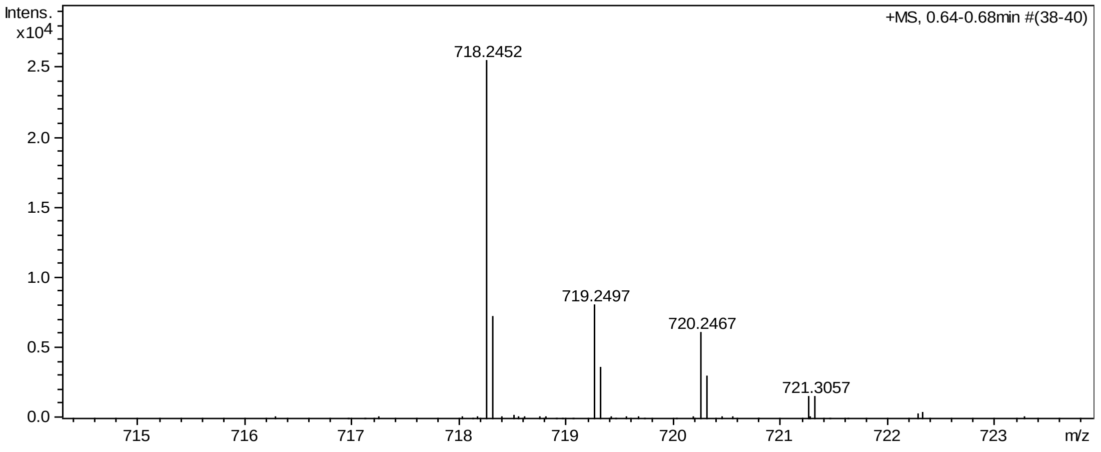

***rel*-(3*R*,3'*S*,4'*R*)-1''-Benzyl-1-butyl-5,5''-dichloro-4'-(2-hydroxy-6-oxocyclohex-1-en-1-yl)-5'-(*p*-tolyl)-4'*H*-dispiro[indoline-3,2'-pyrrole-3',3''-indoline]-2,2''-dione (4m):** White solid, 65%, m.p. 238-239ºC; ^{1}H NMR (400 MHz,$CDCl_3$) δ: 10.78 (s, 1H, OH), 7.77 (d, *J* = 8.4 Hz, 2H, ArH), 7.73 (d, *J* = 2.0 Hz, 1H, ArH), 7.30 - 7.28 (m, 1H, ArH), 7.22 - 7.20 (m, 5H, ArH), 7.04 (d, *J* = 2.0 Hz, 1H, ArH), 6.99 - 6.97 (m, 1H, ArH), 6.87 - 6.85 (m, 2H, ArH), 6.68 (d, *J* = 8.4 Hz, 1H, ArH), 6.32 (d, *J* = 8.4 Hz, 1H, ArH), 5.52 (s, 1H, CH), 5.14 (d, *J* = 16.4 Hz, 1H, CH_2), 4.49 (d, *J* = 16.0 Hz, 1H, CH_2), 3.80 - 3.73 (m, 1H, CH_2), 3.31 (m, 1H, CH_2), 2.54 - 2.50 (m, 1H, CH_2), 2.40 (s, 3H, CH_3), 2.38 (s, 1H, CH_2), 2.38 - 2.24 (m, 1H, CH_2), 1.92 - 1.77 (m, 3H, CH_2), 1.37 - 1.32 (m, 2H, CH_2),1.11 - 1.05 (m, 2H, CH_2), 0.83 (t, *J* = 16.0 Hz, 3H, CH_3) ppm; ^{13}C NMR (400 MHz, $CDCl_3$) δ: 181.9, 176.9, 176.9, 174.8, 142.7, 142.4, 141.9, 134.8, 129.8, 129.8, 129.2, 128.9, 128.8, 128.7, 128.6, 128.6, 128.5, 128.2, 127.4, 126.9, 126.6, 126.5, 125.7, 113.3, 109.9, 109.8, 109.8, 109.7, 61.8, 54.2, 54.2, 43.8, 40.8, 36.3, 29.3, 21.6, 21.6, 20.0, 13.6 ppm; IR (KBr) υ: 3754, 3056, 2983, 2863, 2855, 1863, 1745, 1682, 1632, 1526,1386, 1266, 1186, 1063, 963, 873 cm^{-1}; HRMS (ESI-TOF) Calcd. for $C_{42}H_{38}ClN_3O_4$ ($[M+H]^+$): 718.2234, Found: 718.2225.

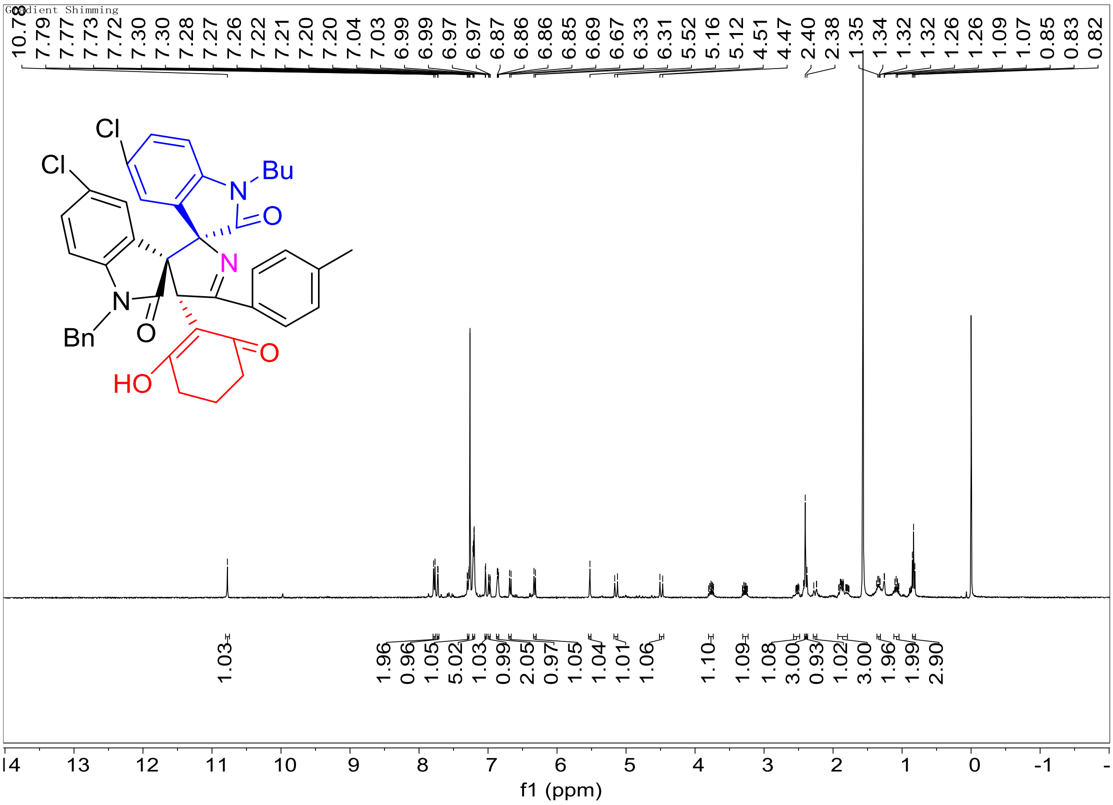

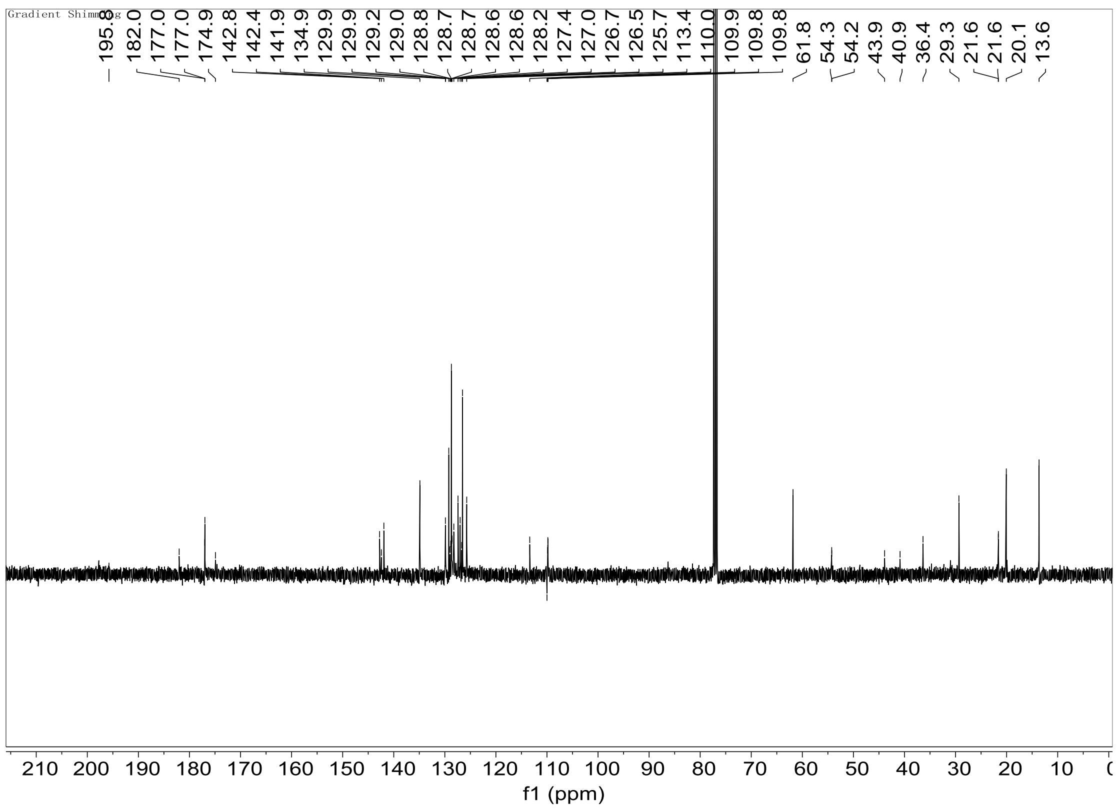
Gradient Shimming
195.8
182.0
177.0
177.0
174.9
142.8
142.4
141.9
134.9
129.9
129.9
129.2
129.0
128.8
128.7
128.7
128.6
128.6
128.2
127.4
127.0
126.7
126.5
125.7
113.4
110.0
109.9
109.8
109.8
61.8
54.3
54.2
43.9
40.9
36.4
29.3
21.6
21.6
20.1
13.6
210 200 190 180 170 160 150 140 130 120 110 100 90 80 70 60 50 40 30 20 10 0
f1 (ppm)

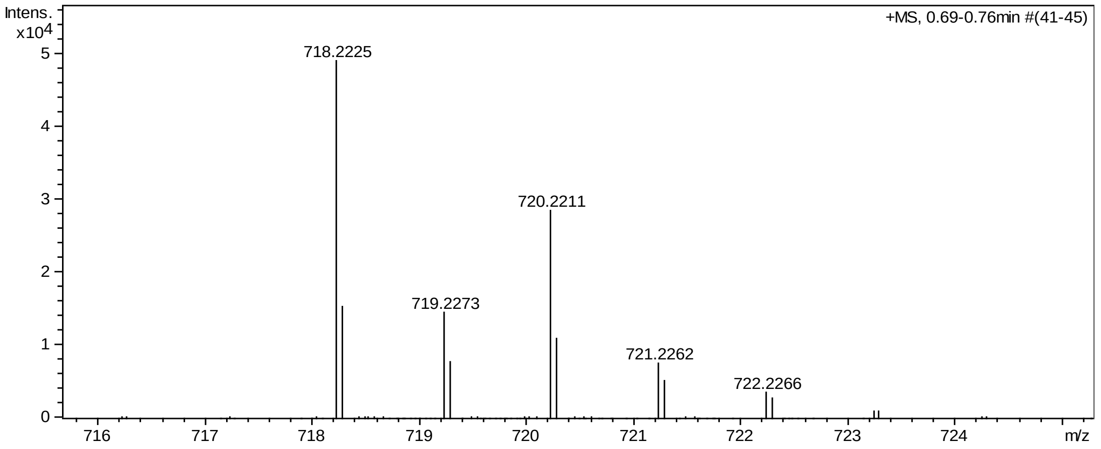
Intens.
x10^4
+MS, 0.69-0.76min #(41-45)
718.2225
719.2273
720.2211
721.2262
722.2266
716 717 718 719 720 721 722 723 724 m/z

***rel*-(3*R*,3'*S*,4'*R*)-1''-Benzyl-5''-chloro-4'-(2-hydroxy-6-oxocyclohex-1-en-1-yl)-5-methyl-5'-(*p*-tolyl)-4'*H*-dispiro[indoline-3,2'-pyrrole-3',3''-indoline]-2,2''-dione (4n):** White solid, 52%, m.p. 242-245°C; ^{1}H NMR (400 MHz,$CDCl_3$) δ: 10.61 (s, 1H, OH), 7.79 (d, *J* = 8.0 Hz, 2H, ArH), 7.49 (s, 1H, ArH), 7.38 (s, 1H, NH), 7.20 (d, *J* = 8.4 Hz, 2H, ArH), 7.15 (d, *J* = 7.2 Hz, 1H, ArH), 7.12 - 7.06 (m, 3H, ArH), 7.04 (d, *J* = 2.0 Hz, 1H, ArH), 7.00 - 6.97 (m, 1H, ArH), 6.67 (d, *J* = 8.0Hz, 1H, ArH), 6.64 (d, *J* = 7.2 Hz, 2H, ArH), 6.27 (d, *J* = 8.4 Hz, 1H, ArH), 5.52 (s, 1H, CH), 5.25 (d, *J* = 16.4 Hz, 1H, CH_2), 4.38 (d, *J* = 16.4, 1H, CH_2), 2.55 - 2.48 (m, 1H, CH_2), 2.43 - 2.36 (m, 1H, CH_2), 2.40 (s, 3H, CH_3), 2.27 - 2.22 (m, 1H, CH_2), 2.13 (s, 3H, CH_3), 1.91 - 1.79 (m, 3H, CH_2) ppm; ^{13}C NMR (400 MHz, $CDCl_3$) δ: 180.4, 179.8, 177.8, 176.0, 173.8, 141.2, 136.9, 133.9, 129.2, 128.1, 127.6, 127.5, 127.2, 126.1, 126.0, 125.1, 125.1, 124.5, 112.4, 112.4, 109.1, 108.8, 60.9, 59.4, 53.1, 42.5, 35.3, 30.0, 30.0, 21.6, 20.6, 20.0, 20.0, 18.9, 13.2 ppm; IR (KBr) υ: 3763, 3562, 3061, 3010, 2936, 2846, 1876, 1742, 1692, 1600, 1326, 1185, 1023, 953, 842 cm^{-1}; HRMS (ESI-TOF) Calcd. for $C_{39}H_{33}ClN_3O_4$ ($[M+H]^+$): 642.2154, Found: 642.2149.

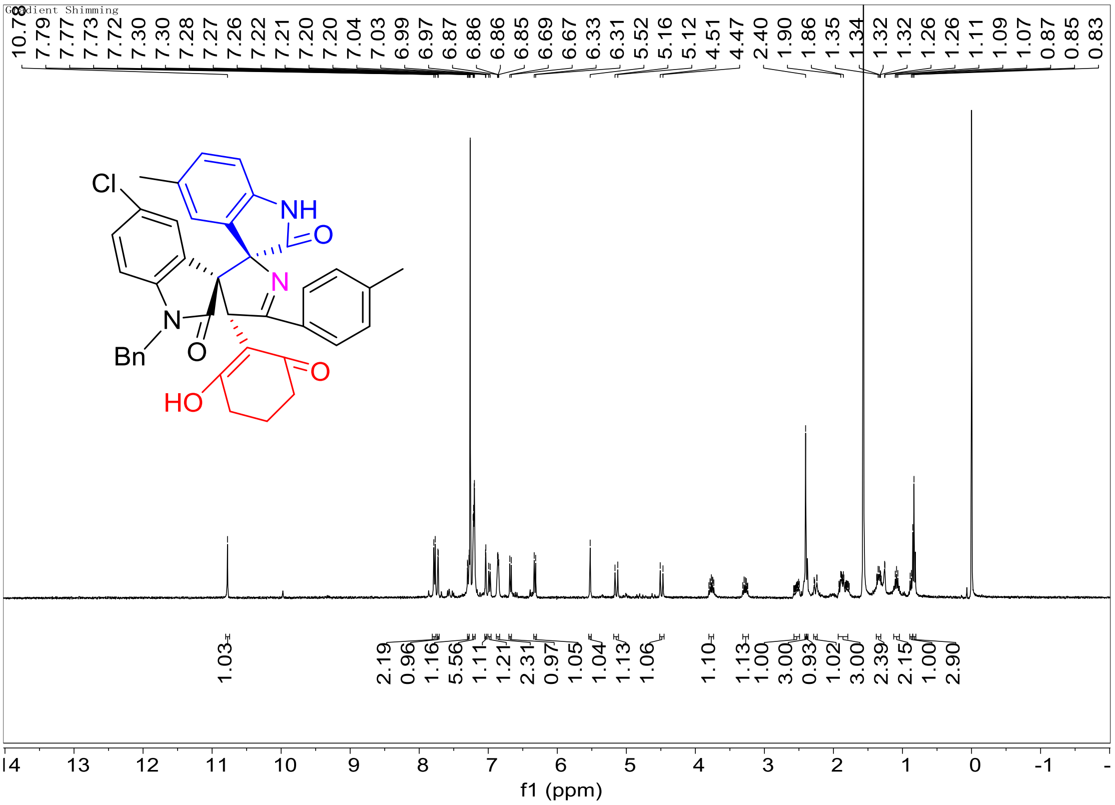

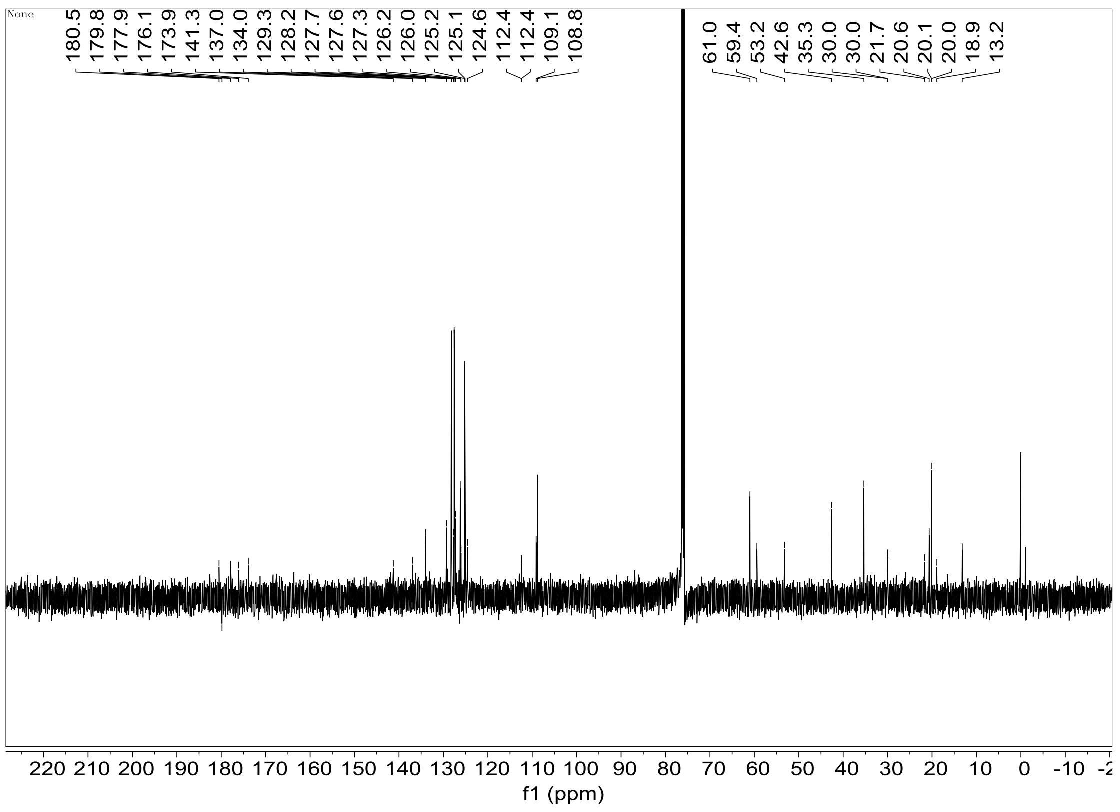
None
180.5
179.8
177.9
176.1
173.9
141.3
137.0
134.0
129.3
128.2
127.7
127.6
127.3
126.2
126.0
125.2
125.1
124.6
112.4
112.4
109.1
108.8
61.0
59.4
53.2
42.6
35.3
30.0
30.0
21.7
20.6
20.1
20.0
18.9
13.2
220 210 200 190 180 170 160 150 140 130 120 110 100 90 80 70 60 50 40 30 20 10 0 -10
f1 (ppm)

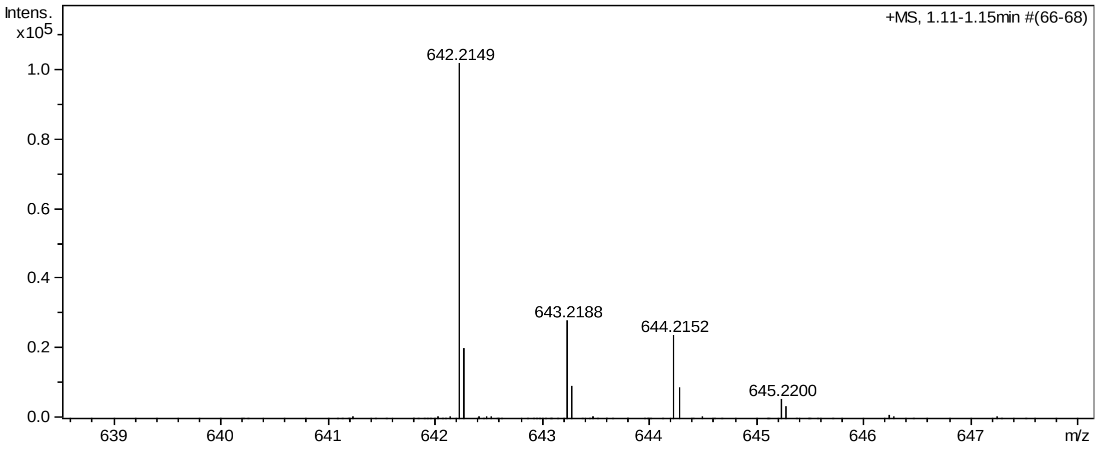
Intens.
x10^5
+MS, 1.11-1.15min #(66-68)
642.2149
643.2188
644.2152
645.2200
1.0
0.8
0.6
0.4
0.2
0.0
639
640
641
642
643
644
645
646
647
m/z

***rel*-(3*R*,3'*S*,4'*R*)-1''-Benzyl-1-butyl-5''-chloro-4'-(2-hydroxy-6-oxocyclohex-1-en-1-yl)-5-methyl-5'-(*p*-tolyl)-4'*H*-dispiro[indoline-3,2'-pyrrole-3',3''-indoline]-2,2''-dione (4o):** White solid, 63%, m.p. 132-133ºC; ^{1}H NMR (400 MHz,$CDCl_3$) δ: 11.08 (s, 1H, OH), 7.79 (d, *J* = 8.4 Hz, 2H, ArH), 7.49 (s, 1H, ArH), 7.20 (d, *J* = 8.0 Hz, 2H, ArH), 7.17 - 7.09 (m, 4H, ArH), 7.07 (d, *J* = 2.0 Hz, 1H, ArH), 6.69 - 6.94 (m, 1H, ArH), 6.68 - 6.64 (m, 3H, ArH), 6.24 (d, *J* = 8.4 Hz, 1H, ArH), 5.52 (s, 1H, CH), 5.19 (d, *J* = 16.8 Hz, 1H, CH_2), 4.41 (d, *J* = 16.4, 1H, CH_2), 3.77 -3.70 (m, 2H, CH_2), 3.31 - 3.25 (m, 1H, CH_2), 2.58 - 2.50 (m, 1H, CH_2), 2.48 - 2.37 (m, 1H, CH_2), 2.38 (s, 3H, CH_3), 2.28 - 2.23 (m, 1H, CH_2), 2.14 (s, 3H, CH_3), 1.93 - 1.78 (m, 3H, CH_2), 1.38 - 1.33 (m, 2H, CH_2), 1.14 - 1.06 (m, 2H, CH_2), 0.83 (t, *J* = 7.2 Hz, 3H, CH_3) ppm; ^{13}C NMR (600 MHz, $CDCl_3$) δ: 197.9, 181.4, 177.2, 177.1, 174.9, 142.8, 142.1, 140.9, 135.0, 134.1, 130.2, 130.1, 129.1, 128.8, 128.6, 128.5, 128.3, 127.1, 126.8, 126.2, 126.1, 125.7, 113.5, 109.7, 108.6, 61.9, 54.0, 43.6, 40.7, 36.4, 31.0, 29.4, 21.6, 21.0, 20.1, 19.9, 13.7; IR (KBr) υ: 3741, 3056, 3021, 2993, 2845, 2813, 1883, 1772, 1687, 1632, 1274, 1123, 1045, 956, 852, 831, 741 cm^{-1}; HRMS (ESI-TOF) Calcd. for $C_{43}H_{41}ClN_3O_4$ ($[M+H]^+$): 698.2780, Found: 698.2770.

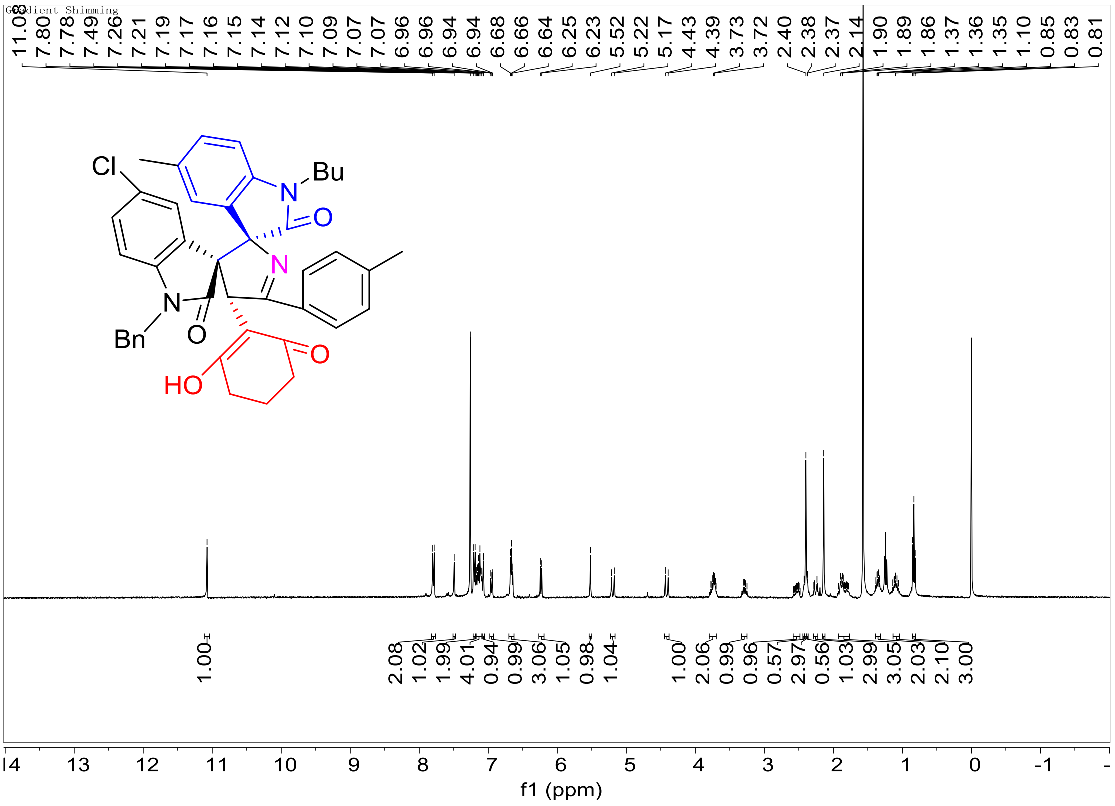

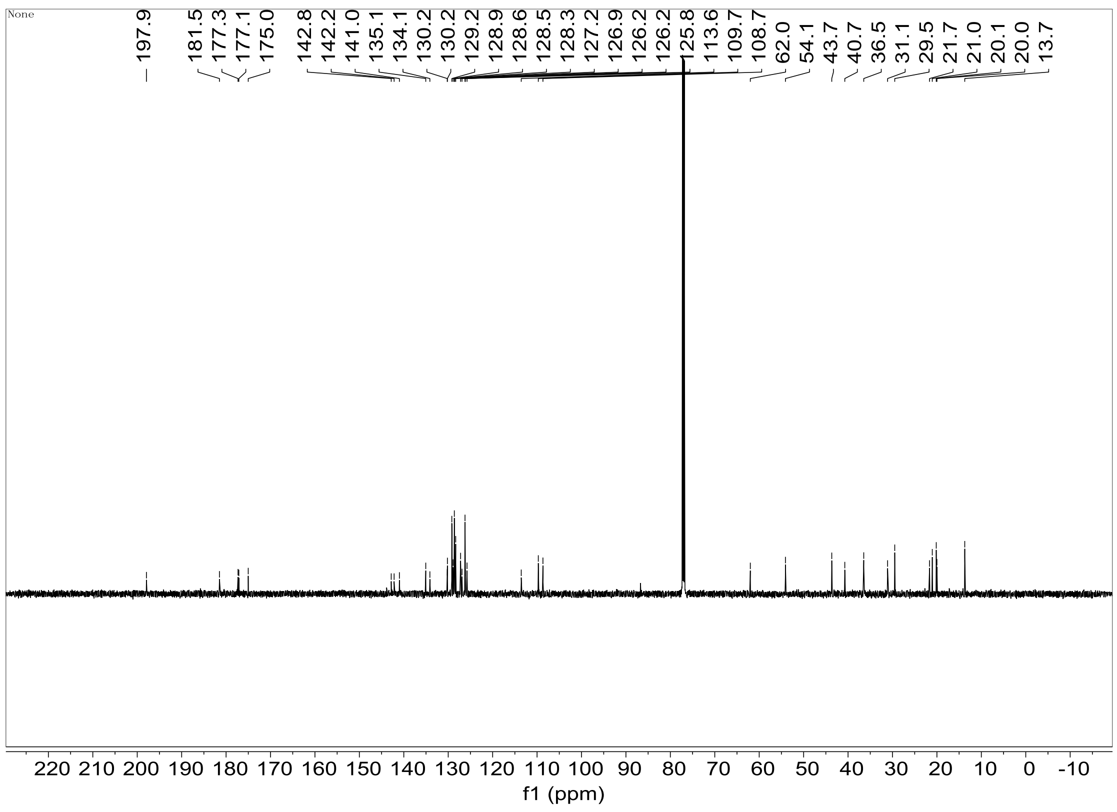
None
197.9
181.5
177.3
177.1
175.0
142.8
142.2
141.0
135.1
134.1
130.2
130.2
129.2
128.9
128.6
128.5
128.3
127.2
126.9
126.2
126.2
125.8
113.6
109.7
108.7
62.0
54.1
43.7
40.7
36.5
31.1
29.5
21.7
21.0
20.1
20.0
13.7
220 210 200 190 180 170 160 150 140 130 120 110 100 90 80 70 60 50 40 30 20 10 0 -10
f1 (ppm)

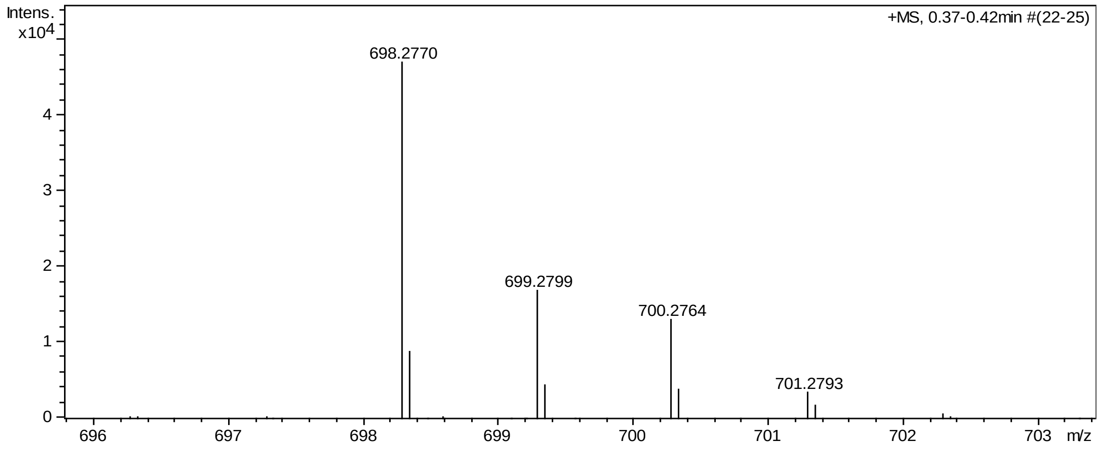
Intens.
x10^4
+MS, 0.37-0.42min #(22-25)
698.2770
699.2799
700.2764
701.2793
0
1
2
3
4
696
697
698
699
700
701
702
703
m/z

***rel*-(3*R*,3'*S*,4'*R*)-1,1''-Dibenzyl-5''-chloro-5'-(4-chlorophenyl)-4'-(2-hydroxy-6-oxocyclohex-1-en-1-yl)-5-methyl-4'*H*-dispiro[indoline-3,2'-pyrrole-3',3''-indoline]-2,2''-dione (4p):** White solid, 73%, m.p. 255-256°C; ^{1}H NMR (400 MHz,$CDCl_3$) δ: 10.93 (s, 1H, OH), 7.86 (d, *J* = 8.4 Hz, 2H, ArH), 7.50 (s, 1H, ArH), 7.39 (d, *J* = 8.4 Hz, 2H, ArH), 7.21 - 7.16 (m, 2H, ArH), 7.13 (d, *J* = 8.0 Hz, 2H, ArH), 7.09 (s, 1H, ArH), 7.07 - 7.06 (m, 2H, ArH), 7.01 - 6.99 (m, 2H, ArH), 6.77 (d, *J* = 7.2 Hz, 2H, ArH), 6.66 (d, *J* = 7.6 Hz, 2H, ArH), 6.48 (d, *J* = 7.6 Hz, 1H, ArH), 5.55 (s, 1H, CH), 5.18 (d, *J* = 16.8 Hz, 1H, CH_2), 5.07 (d, *J* = 15.6 Hz, 1H, CH_2), 4.48 (d, *J* = 8.8 Hz, 1H, CH_2), 4.44 (d, *J* = 8.8 Hz, 1H, CH_2), 2.51 (d, *J* = 7.2 Hz, 1H, CH_2), 2.44 - 2.39 (m, 1H, CH_2), 2.27 (d, *J* = 16.0 Hz, 1H, CH_2), 2.11 (s, 3H, CH_3), 1.93 - 1.77 (m, 3H, CH_2) ppm; ^{13}C NMR (600 MHz, $CDCl_3$) δ: 197.7, 180.6, 177.1, 177.1, 175.0, 142.8, 140.5, 137.9, 134.7, 134.4, 133.8, 131.2, 130.4, 129.6, 128.7, 128.6, 127.6, 127.3, 127.1, 126.8, 126.5, 126.1, 126.0, 125.8, 113.2, 109.9, 109.9, 109.8, 109.8, 109.8, 87.2, 61.9, 54.0, 36.3, 31.0, 21.0, 20.9, 19.8 ppm; IR (KBr) υ: 3692, 3052, 3026, 2995, 2864, 2841, 1872, 1741, 1654, 1611, 1321, 1185, 1024, 951, 852, 768 cm^{-1}; HRMS (ESI-TOF) Calcd. for $C_{45}H_{36}Cl_2N_3O_4$ ([M+H]$^+$): 752.2077, Found: 752.2070.

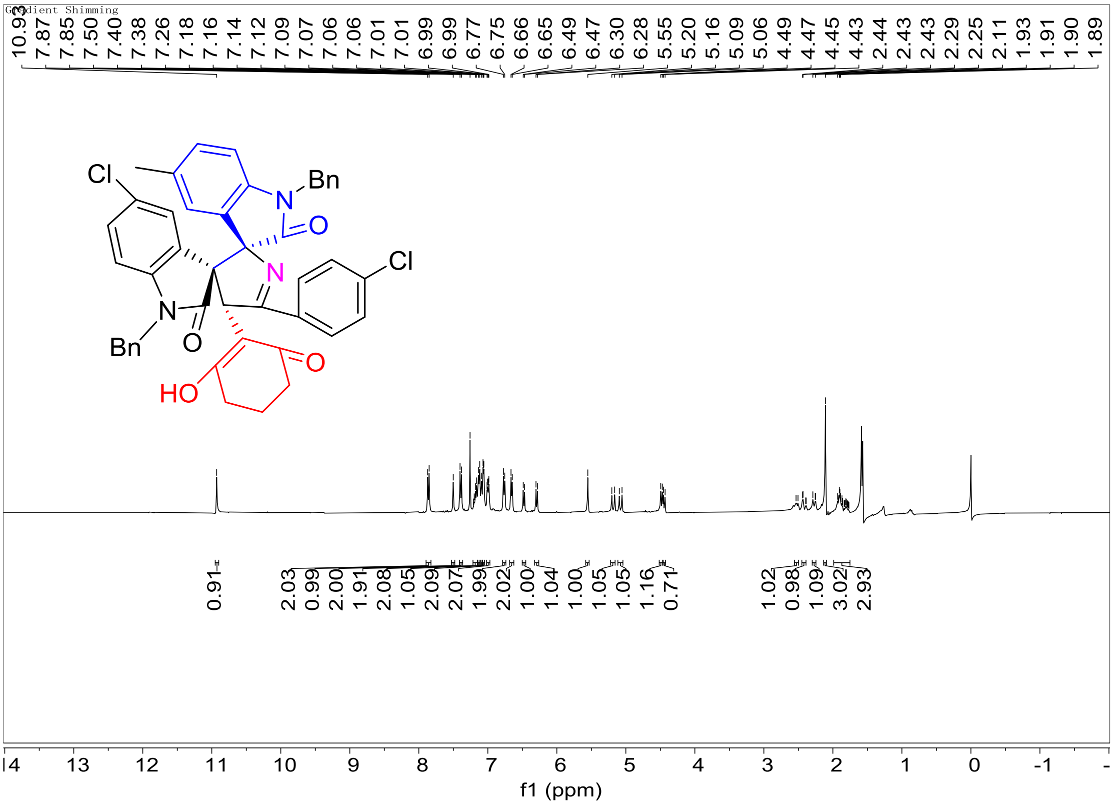

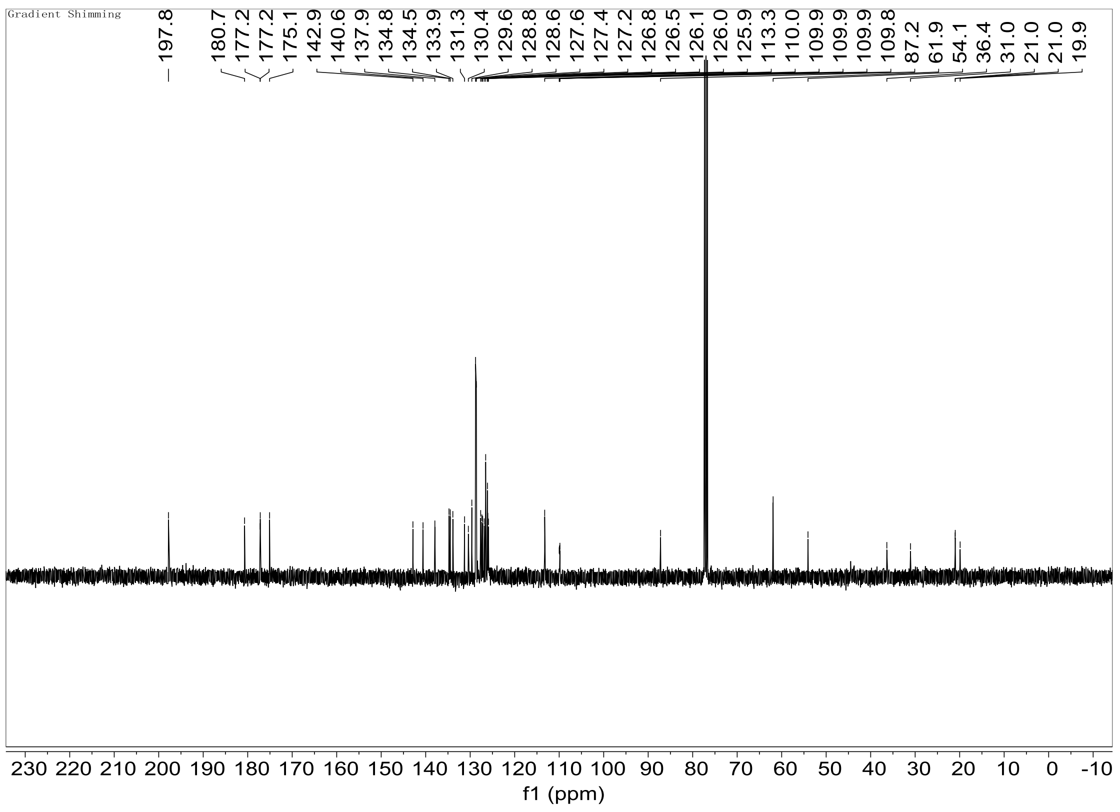
Gradient Shimming
197.8
180.7
177.2
177.2
175.1
142.9
140.6
137.9
134.8
134.5
133.9
131.3
130.4
129.6
128.8
128.6
127.6
127.4
127.2
126.8
126.5
126.1
126.0
125.9
113.3
110.0
109.9
109.9
109.9
109.8
87.2
61.9
54.1
36.4
31.0
21.0
21.0
19.9
230 220 210 200 190 180 170 160 150 140 130 120 110 100 90 80 70 60 50 40 30 20 10 0 -10
f1 (ppm)

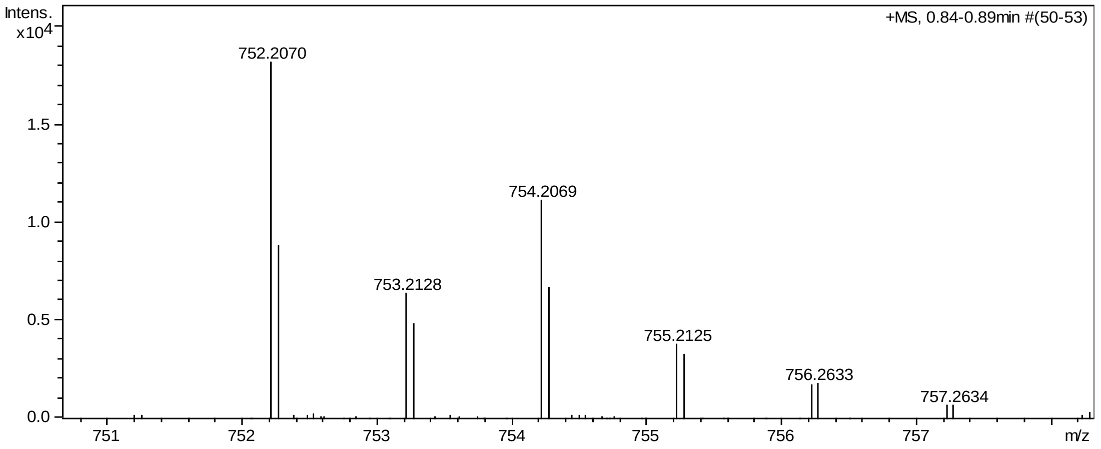
Intens.
x10^4
+MS, 0.84-0.89min #(50-53)
752.2070
754.2069
753.2128
755.2125
756.2633
757.2634
1.5
1.0
0.5
0.0
751
752
753
754
755
756
757
m/z
